# Biophysical and anthropogenic influences on the status of Tonga’s coral reefs and reef fish fishery

**DOI:** 10.1371/journal.pone.0241146

**Published:** 2020-11-17

**Authors:** Patrick Smallhorn-West, Sophie Gordon, Karen Stone, Daniela Ceccarelli, Siola’a Malimali, Tu’ikolongahau Halafihi, Mathew Wyatt, Tom Bridge, Robert Pressey, Geoffrey Jones

**Affiliations:** 1 Marine Biology and Aquaculture, College of Science and Engineering, James Cook University, Townsville, QLD, Australia; 2 Australian Research Council Centre of Excellence for Coral Reef Studies, James Cook University, Townsville, QLD, Australia; 3 WorldFish, Jalan Batu Maung, Bayan Lepas, Penang, Malaysia; 4 Faculty of Science, Health, Education and Engineering, University of the Sunshine Coast, Sippy Downs, QLD, Australia; 5 Vava’u Environmental Protection Association (VEPA), Neiafu, Vava’u, Tonga; 6 Ministry of Fisheries, Nuku’alofa, Tongatapu, Tonga; 7 Australian Institute of Marine Science, Cape Cleveland, QLD, Australia; 8 Biodiversity and Geosciences Program, Museum of Tropical Queensland, Queensland Museum Network Townsville, QLD, Australia; Leibniz Centre for Tropical Marine Research, GERMANY

## Abstract

Despite increasing threats to Tonga’s coral reefs from stressors that are both local (e.g. overfishing and pollution) and global (e.g. climate change), there is yet to be a systematic assessment of the status of the country’s coral reef ecosystem and reef fish fishery stocks. Here, we provide a national ecological assessment of Tonga’s coral reefs and reef fish fishery using ecological survey data from 375 sites throughout Tonga’s three main island groups (Ha’apai, Tongatapu and Vava’u), represented by seven key metrics of reef health and fish resource status. Boosted regression tree analysis was used to assess and describe the relative importance of 11 socio-environmental variables associated with these key metrics of reef condition. Mean live coral cover across Tonga was 18%, and showed a strong increase from north to south correlated with declining sea surface temperature, as well as with increasing distance from each provincial capital. Tongatapu, the southernmost island group, had 2.5 times greater coral cover than the northernmost group, Vava’u (24.9% and 10.4% respectively). Reef fish species richness and density were comparable throughout Tongatapu and the middle island group, Ha’apai (~35 species/transect and ~2500 fish/km^2^), but were significantly lower in Vava’u (~24 species/transect and ~1700 fish/km^2^). Spatial patterns in the reef fish assemblage were primarily influenced by habitat-associated variables (slope, structural complexity, and hard coral cover). The biomass of target reef fish was greatest in Ha’apai (~820 kg/ha) and lowest in Vava’u (~340 kg/ha), and was negatively associated with higher human influence and fishing activity. Overall mean reef fish biomass values suggest that Tonga’s reef fish fishery can be classified as moderately to heavily exploited, with 64% of sites having less than 500 kg/ha. This study provides critical baseline ecological information for Tonga’s coral reefs that will: (1) facilitate ongoing management and research; and (2) enable accurate reporting on conservation targets locally and internationally.

## Introduction

Coral reefs are increasingly threatened by cumulative human-induced disturbances [1, 2]. These range from large-scale global impacts such as climate-driven coral bleaching [3, 4], to more local stressors such as overfishing, destructive fishing practices, and pollution [5]. Furthermore, many of these impacts are increasing both in frequency and severity [6]. Despite the widespread acknowledgment of large-scale coral reef decline, many reef ecosystems remain poorly studied, with little data available to accurately quantify current ecosystem condition [7]. In addition, the specific ecological and socioeconomic factors associated with key metrics of reef health are often unknown (but see [8–10]). Managing the multiple threats facing coral reef ecosystems requires accurate data on both the status of the ecosystems and the dominant influences on reef condition.

Assessing the ecological status of a country’s coral reefs and associated fishery resources requires a comprehensive assessment of both benthic habitat structure, reef fish communities, and exploited species at large spatial scales [11, 12]. Within this context, a number of metrics are currently considered particularly important. Hard corals are the dominant ecosystem engineers on coral reefs, providing both food and three-dimensional structure for reef-associated fish and fisheries [13–15]. The proportional cover of live hard corals on a reef is therefore one of the key variables used to measure reef health because high coral cover is a generally accepted desirable state for coral reef benthic communities [16–18]. In addition, the proportional coverage of other benthic categories, such as soft corals, crustose coralline algae (CCA) and turfing algae, are also considered important for understanding overall reef condition [19]. Given the importance placed on biodiversity conservation, reef fish species richness is also commonly used as a metric of reef status, under the assumption that areas with higher species richness are more likely to contribute to both biodiversity targets and ecosystem function [20]. The overall density of reef fish is also a common metric that is used as a proxy indicator of reef condition and to quantify differences between sites [21]. In addition to ecosystem condition, characterizing the status of multi-species fisheries, which are typical for coral reef environments, often creates challenges due to the many life-history traits within a single fishery [22]. To handle this complexity, the biomass of target reef fish species has been demonstrated as a key proxy for the status of reef fish fisheries, with predictable declines in ecosystem condition as biomass diminishes [23–25].

Coral reef community structure and condition is likely to be determined by complex interactions between socioeconomic and environmental variables [26–28]. Managing reef ecosystems therefore relies not only on quantifying current reef community structure, but also on understanding the patterns and processes responsible for observed conditions [18]. While investigating influences on reef community structure has been underway for decades, more recent advances in our ability to measure and analyse many socio-environmental variables concurrently has enabled simultaneous examination of the relationships and interactions between a broad range of variables [18, 26]. However, in developing countries, few resources are available to monitor national reef status, so management and governing authorities are often required to make wide-reaching decisions for both people and ecosystems based on limited information. When records are unavailable, reporting on both national and international commitments can rely on data of questionable quality or limited scope, resulting in false impressions of progress [29]. Good quality data at the correct spatial scale are therefore critical to maintain government accountability and understand the efficacy of management strategies.

As with many other South Pacific nations, coral reefs in Tonga are increasingly threatened [7]. In the past decade alone, five severe tropical cyclones (Category 4–5) have affected Tonga (Wilma 2011, Evan 2012, Ian 2014, Wintson 2015 and Gita 2018), and coral bleaching events were reported in 2012, 2014 and 2016 (personal communication, Vava’u Environmental Protection Association (VEPA)). Concerns about overfishing and destructive fishing practices have also been raised for decades, with multiple management strategies employed with varying degrees of success [30]. Land-based pollution from agricultural runoff and illegal dumping of both rubbish and sewage are also a concern, particularly around lagoonal areas in the island groups of Tongatapu and Vava’u [31]. However, few data are available to determine the consequences of these pressures on reef communities or food security [32].

While several local-scale projects and reports exist (Table 1), there is yet to be a systematic assessment of Tonga’s coral reef ecosystems and reef fish fishery at the national level. The fifth national report to the Convention on Biological Diversity (32) described marine biodiversity trends as “not clearly defined” (page 62) and “unknown”, and while “the lack of resource assessment is the key issue for [Tonga’s] marine ecosystem, only few select fisheries are known” (page 59). Likewise, the status of coral reefs in the Pacific for 2011 [7] described the status, health and resilience of Tonga’s coral reefs as “data deficient” or “not considered”, and that “the available data are insufficient to describe the health and resilience of these reefs (and) there has been little scientific monitoring and assessment of most reef areas and many have not been mapped or surveyed” (page 199). In more recent years several expeditions have conducted ecological surveys, primarily in the Vava’u group and northern Ha’apai (summarized by [5]). The most notable two studies were Atherton et al. [33] and Purkis et al. [34]. Purkis et al. [34] surveyed coral reefs at 60 sites in Vava’u and northern Ha’apai as part of the 2013 global Living Oceans Foundation expedition. Atherton et al. [33] conducted a rapid biodiversity assessment (BioRap) on coral and reef fish communities at twenty-seven sites in the Vava’u archipelago.

**Table 1:** Literature available on the status of Tonga’s coral reef ecosystem.

Publication	Location	Additional information
Adjeroud et al., 2013 [35]	Tongatapu	Examined spatial distribution of coral assemblages across ten sites in the lagoon of Tongatapu.
Aholahi et al., 2017 [31]	Tongatapu	Detailed current status of the Fanga’uta lagoon in Tongatapu, including benthic assemblages and water quality. Earlier reports are also available.
Atherton et al., 2015 [33]	Vava’u	BioRap rapid assessment of biodiversity surveys conducted throughout the Vava’u archipelago including reef fish, invertebrates and benthic composition.
Bruckner, 2014 [36]	Ha’apai, Niuatoputapu and Vava’u	Initial report of reef fish, invertebrates and benthic assemblages surveyed across 59 sites as part of the global Khaled bin Sultan Living Ocean Foundation reef expedition. See Purkis et al. (2020) below.
Buckley et al., 2017 [37]	Vava’u	Eleven sites established in the Vava’u archipelago as permanent benthic monitoring sites.
Chin et al., 2011 [7]	National	Synthesis as of 2011 of the current known status of Tonga’s coral reef ecosystems. Conclusions about status varied between data deficient, not considered or low confidence
Ceccarelli, 2016 [38]	Vava’u	Baseline ecological surveys across 36 sites for seven Special Management Area (SMA) communities. Included benthic composition, invertebrates and reef fish. Data from these surveys are also included in this report.
Friedman et al., 2008 [39]	Two villages in each of Ha’apai and Tongatapu	Part of the PROCFish/C program to provide baseline information on the status of reef fisheries. Reef fish, benthic and invertebrate surveys were conducted around two villages in both Ha’apai and Tongatapu.
Government of Tonga, 2014 [32]	National	National report by the Tongan government to the Convention on Biological Diversity on the current status of Tonga’s environment, including coral reefs. Coral reef ecosystems were classified as primarily data deficient or unknown.
Holthus, 1996 [40]	Vava’u	Coral assemblages across thirty-six sites in the Vava’u archipelago were surveyed in 1990 to determine their suitability for coral harvesting.
Kronen, 2004 [41]	Around two villages in each of Ha’apai, Tongatapu and Vava’u	Underwater visual census of target reef fish and total reef fish size, density and diversity were conducted around several villages in each island group.
Lovell & Palaki, 2000 [42]	National	Ecological surveys conducted of benthic assemblages and reef fish, although extent is unclear.
Malimali, 2013 [43]	Five communities across Ha’apai, Tongatapu and Vava’u and associated comparison sites	Reef fish, invertebrates and benthic composition were compared between managed and open areas for five communities as part of PhD thesis.
Mayfield et al., 2017 [44]	Ha’apai, Niuatoputapu and Vava’u	Part of the Khaled bin Sultan Living Oceans Foundation surveys of 59 reefs in Tonga. *Pocillopora damicornis* and *Pocillopora acuta* colonies were sampled to determine whether they differed physiologically although being difficult to distinguish *in-situ*.
Pakoa et al., 2008 [45]	Tongatapu lagoon	Extensive ecological surveys conducted of invertebrates and benthic composition around the Tongatapu lagoon, with an emphasis on their relevance for the Trochus fishery.
Purkis et al., 2020 [34]	Ha’apai, Niuatoputapu and Vava’u	Final report of reef fish, invertebrates and benthic assemblages surveyed across 59 sites as part of the global Khaled bin Sultan Living Ocean Foundation reef expedition. See Bruckner (2014) above.
Richardson, 2010 [46]	Five SMA communities across Ha’apai, Tongatapu and Vava’u	Ecological surveys of benthic community composition around five SMA communities and comparison sites.
Smallhorn-West et al. 2019a [47]	Vava’u	Publication as part of this project and data therefore included in this analysis. Predicted the potential recovery of target species biomass under various protected area configurations based on data from 129 sites in Vava’u.
Smallhorn-West et al. 2019b [48]	Southern Ha’apai	Specific surveys of six coral reef sites around the newly erupted Hunga-Tonga Hunga-Ha’apai volcano near southern Ha’apau. Data were not included in this analysis.
Smallhorn-West et al. 2020a [49]	Same data as this manuscript	Impact evaluation of seven Special Management Areas (SMA) in Tonga.
Smallhorn-West et al. 2020b [50]	Same data as this manuscript	Public report on baseline reef condition throughout Tonga.
Stone et al., 2017 [51]	Ha’apai and Vava’u	Reef fish, invertebrate and benthic community composition across 56 sites as part of the WAITT Institute Vava’u Ocean Initiative. Data from these surveys are included in this report.
Stone et al., 2019 [5]	National	Reviews the current known status of coral reefs in Tonga prior to the surveys used in this report. Conclusions derived mainly from Atherton et al. (2015).
Vieux et al., 2005 [52]	National	Discusses monitoring in the South Pacific, including Tonga. Concludes that while “efforts are now under way to conduct baseline and monitoring studies … there are considerable constraints due to poor capacity for monitoring, surveillance and enforcement”.
Vieux, 2005 [53]	Two villages in Vava’u	Reef fish, invertebrate and benthic community composition at two villages in Vava’u.

This list includes only publications and reports that present ecological data on metrics of reef health or reef fish fisheries. It does not include publications or reports that describe only livelihoods, fishing activities, or management.

A clear gap exists in the information available to support a national-level assessment of Tonga’s coral reefs and reef fish fishery for government, managers, and other stakeholders. The aim of this study was therefore to compile and analyse the first national dataset on the current ecological status of Tonga’s coral reefs (375 sites) and provide baseline ecological information to facilitate ongoing management and research. In addition, we used boosted regression trees to test the relative association of eleven socio-environmental variables with seven key metrics of reef health. Specifically, we ask: i) What are the differences and similarities in benthic cover (hard coral, soft coral, CCA and turf algae), fish diversity and abundance, and target fish biomass among the main island groups of Tonga? and ii) What are the most influential variables associated with reef status across Tonga’s coral reef ecosystem?

## Methods

### Survey design

**Fig 1:**
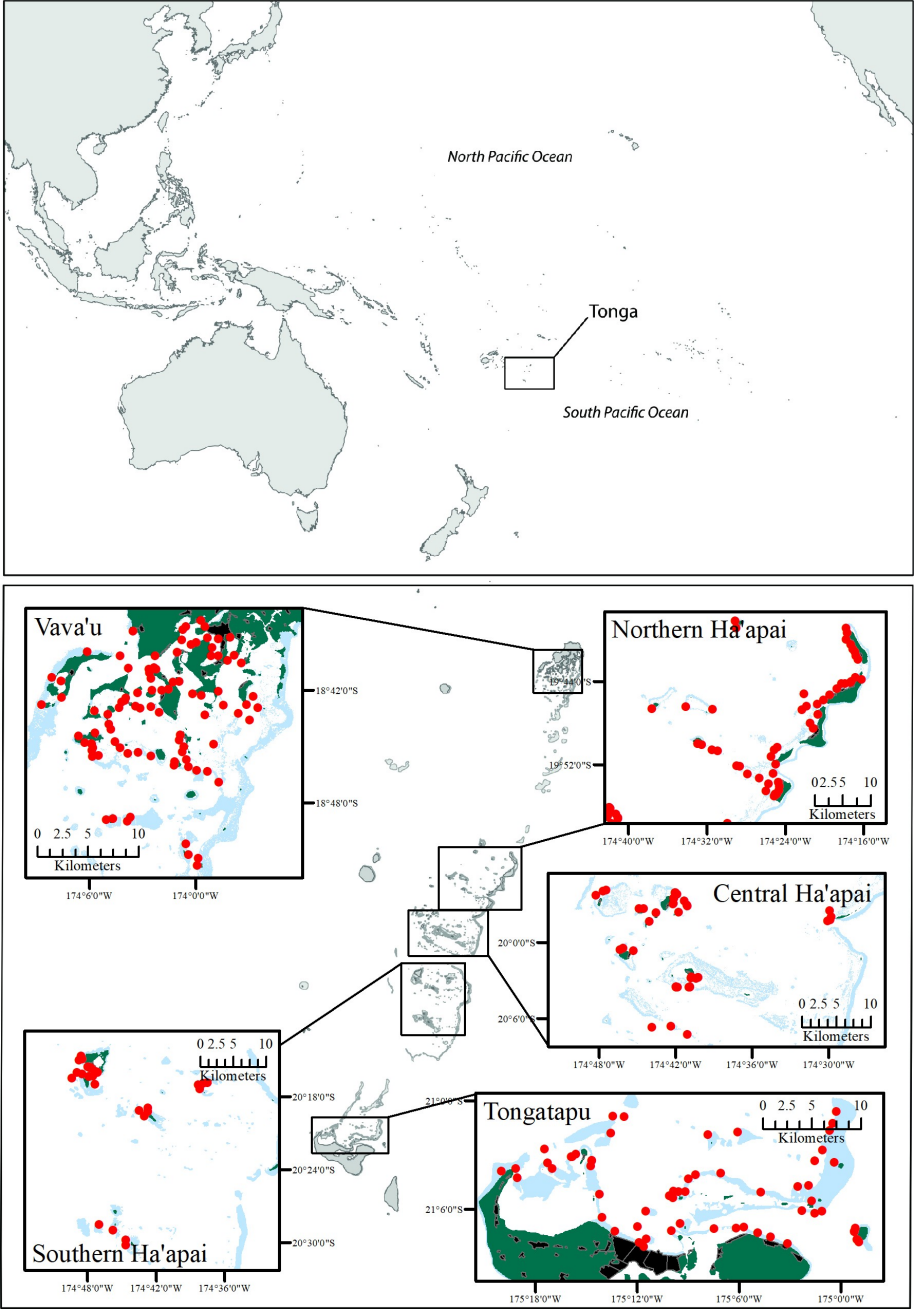
Map of Tonga showing the locations of ecological survey sites in red. Green represents land, black with grey outlines indicate villages, and blue shows coral reefs. Number of sites in Tongatapu = 60, Ha’apai = 143 and Vava’u = 172.

From 2016 to 2018, 375 sites were surveyed across Tonga as part of four separate projects but using a standardized methodology (for individual reports see Ceccarelli [38] and Stone et al. [51]) (Fig 1, Table 2). Underwater visual census was used to survey fish and benthic community composition around the three main island groups of Tonga: Tongatapu, Ha’apai and Vava’u. Due to the large latitudinal gradient across Ha’apai, this island group was further divided into southern, central and northern Ha’apai. All research activities were conducted in accordance with James Cook University Animal Ethic Guidelines (permit approval A2454) and approved by the Tongan Prime Minister’s Office and Tongan Ministry of Fisheries.

**Table 2:** Summary of fish survey data sets available to the project.

Project	Department	Funding	Island group	Number of sites	Year
James Cook University National monitoring project	Ministry of Fisheries	James Cook University	Tongatapu	60	2018
		ARC CoE CRS	Ha'apai	125	2018
		McIntyre Adventure/ Halaevalu Mata’aho Marine Discovery Centre	Vava'u	93	2017
		National Geographic Society			
ADB Vava'u Special Management Areas baseline surveys [38]	Ministry of Fisheries	ADB	Vava'u	36	2016
	Department of Environment				
	VEPA				
VEPA Special Management Areas baseline surveys	VEPA	VEPA	Vava'u	4	2017
WAITT Institute field surveys [52]	Department of Environment	WAITT Institute	Ha'apai	18	2017
	VEPA		Vava'u	39	2017

ARC CoE CRS = Australian Research Council Centre of Excellence for Coral Reef Studies. ADB = Asian Development Bank. VEPA = Vava’u Environmental Protection Association.

At each site, three to six 30 m transects were deployed parallel to the depth contour in depths ranging from two to twelve meters, depending on the reef slope, depth and topography at each site. The abundance and size of all large mobile fish were recorded to the species level within a five-metre belt along each transect. All small, site-attached reef fish species were recorded along a two-metre belt width. The length and abundance of reef fish were converted to biomass following published length-weight relationships for each species [54]. All data were summarized to the site level using mean values.

*In situ* estimates of habitat complexity (rugosity) and reef slope were also collected for each site on a five-point scale from low and sparse relief (score = 1) to exceptionally complex with numerous caves and overhangs (score = 5), and from < 10^o^ (score = 1) to 90^o^ (score = 5), respectively [55].

### Benthic composition

Benthic community composition was estimated using image analysis of ten 1 x 1 m benthic photoquadrats per transect with 15 points randomly overlaid across each image (total 150 points per transect). Given the large number of images and points required for annotation (images = 11020; points = 165300), we used the machine learning software BenthoBox (www.benthobox.com) to assist with the benthic annotations. BenthoBox automatically classifies points into benthic substrate categories from images based on training provided by a human annotator. The aim of the automated annotation method is to learn from human annotations and automatically analyze the remaining images to within an acceptable margin of error [56, 57]. While automation typically captures similar trends but with higher variability than among human annotators [56, 58], the impact of this error on interpretation depends on the relative abundance of organisms, taxonomic resolution, and ecological relevance of the variables in question. Typically, the noise around automated annotations can lead to misinterpretations of rare categories (<5% total cover) for which the average abundance is similar to the error in quantification. However, the impact of error in automated analysis on more dominant benthic groups (>5% total cover) is less pronounced, and usually has marginal effects on derived cover estimates [58]. For the purposes of this study, we therefore included four common benthic categories each with mean cover greater than five percent: hard coral, soft coral, CCA and turf algae. Details of the automated annotation process are available in the S1 File (S1-S4 Figs in S1 File).

A small subset of benthic data was annotated using the point intercept method when photographic equipment was not available. When this was the case, a single benthic point sample was recorded every 50 cm (60 samples per transect; n = 320 transects from 61 sites). In addition, no benthic data were collected at 32 sites surveyed in the Vava’u island group. For these sites, only fish-related analysis was conducted.

### Metrics of reef status and socio-environmental predictor variables

Seven metrics of reef status were used to assess the current ecological condition of Tonga’s coral reef ecosystem and reef fish fishery. Based on acceptable error rates in benthic annotations, benthic response variables were hard coral, soft coral, CCA and turf algae. For the status of coral reef fishes and Tonga’s reef fish fishery, we included total reef fish species richness (n/transect) and density (n/km^2^) and the biomass of target species (kg/ha) larger than 20 cm. We selected this size cut off for biomass because it represents the fishable biomass of target reef fish species that is likely to be targeted by fishers.

Eleven socio-environmental variables known to affect coral reef community structure across Tonga were selected as potential explanatory variables (Table 3). Three of these were collected *in situ* (depth, rugosity and slope) and seven were spatially continuous across Tonga’s coral reefs (cyclone occurrence, distance from provincial capital, fishing pressure, land area, reef density, sea surface temperature and wave energy). Details of how these variables were calculated are available in the S1 File. To control for potential differences in sampling protocols, project (ADB, JCU or WAITT) was also included as an explanatory variable. Due to the small sample size, the VEPA surveys were combined with the WAITT surveys, which happened at a similar time and with the same personnel. Finally, for reef fish metrics, total hard coral cover was also included as an additional explanatory variable.

**Table 3:** Eleven socio-environmental variables included as potential influences on reef condition in Tonga.

Variable	Description
Cyclone occurrence in the past 18 months	Occurrence of sustained wind speeds above 50 knots (category 2 cyclone) within the past 18 months.
Depth	Depth (m), collected *in situ*.
Distance from provincial capital	Distance (km) from the nearest provincial capital town (Tongatapu–Nuku’alofa, Ha’apai–Pangai and Vava’u–Neifau). The provincial capitals are both the main population centres for each island group and the locations of the main fish markets (S6 Fig in S1 File).
Fishing pressure	Normalized (0–100) abundance of commercial and subsistence fishers (adjusted for catch) extrapolated across the coral reefs of Tonga. It constitutes a unit-less value of relative long-term fishing effort throughout the region. This fishing pressure metric also accounts for differences in fishing pressure due to management within marine protected areas (S1 Table in S1 File) (S7 and S8 Figs in S1 File).
Habitat rugosity	Estimate of habitat complexity collected *in situ* on a five-point scale from low and sparse relief (score = 1) to exceptionally complex with numerous caves and overhangs (score = 5).
Hard coral cover	Percent total live hard coral cover. Only included for reef fish variables.
Land area within 5 km	Terrestrial influence calculated as the amount of land (km^2^) within a 5 km radius of each 10m^2^ reef pixel (S9 Fig in S1 File).
Reef density within 5 km	Calculated as the amount of reef habitat (km^2^) within a 5 km radius of each 10m^2^ reef pixel (S5 Fig in S1 File).
Slope	Estimate of reef slope collected *in situ* on a five-point scale from < 10^o^ (score = 1) to 90^o^ (score = 5).
Sea surface temperature (SST)	Mean annual sea surface temperature from 2002–2010 (degrees Celsius) (S10 Fig in S1 File).
Wave energy	Average daily wave energy (joules per m^2^) (S11 Fig in S1 File).

Details of their development are available in the S1 File.

### Data analysis

The seven variables of reef condition were compared between island groups using generalized linear models with Tukey’s post hoc comparisons. Models were fitted with either raw data, log, or log(x+1) transformations and model performance assessed by i) comparing AIC scores ii) visual inspections of qqplots and plotted fitted vs. residuals and iii) calculating goodness of fit and overdispersion. Analysis outputs are available in S1 File (S2 and S3 Tables in S1 File). Patterns in the socio-environmental variables across island groups were explored using principal component ordination (PCO) of normalized data based on Euclidean distance with Primer-e Version 6.

Variables potentially influencing reef condition in Tonga were then explored using boosted regression tree (BRT) models [59, 60]. All BRT models were fitted using the *gbm*.*step* routine in the *dismo* package [60] and the *ggBRT* package [10] within the R statistical and graphical environment [61]. BRTs fit a large succession of simple regression trees that each learn only a small fraction of the data, but with each successive tree focusing on the remaining most prominent patterns. By shrinking the contributions of many trees, BRTs are generally able to make accurate predictions from complex data sets [18]. Overfitting can be countered through cross-validation, which strikes a balance between predictive performance and model fit [62]. BRTs are useful for exploring the relative impacts of a large number of predictors since, unlike linear models, they are not reduced to low-level approximations of system complexity. While BRT predictions are robust to multi-colinearity and non-linearity [63], the relative influence of highly correlated variables (>0.6) can be pooled into one of the variables. Therefore, a correlation matrix was used to determine whether any combination of predictor variables was highly correlated (S12 Fig in S1 File).

Optimal model parameters (bag fraction, tree complexity and learning rate) for each BRT were determined by running all iterations and selecting the one with the greatest explained deviance and a minimum of 1000 trees (S4 and S5 Tables in S1 File) [64]. Based on histograms, BRTs for hard coral, soft coral, CCA, reef fish density, and target biomass were analysed using a Poisson distribution, while turf algae and reef fish species richness were analysed using a Gaussian distribution. Model performance was assessed by 10-fold cross-validation, which tests the model against withheld portions of the data. Following Jouffray et al. [10], cross-validated percent explained deviance was calculated as [1-(cross-validated deviance/mean total deviance)]. Spatial autocorrelation was assessed by estimating Moran’s I from the model residuals (S6 Table in S1 File).

The relative importance of each predictor variable was calculated as the frequency of splits involving each variable weighted by the associated square improvement in the model, averaged over all trees and scaled out of 100 such that larger values signify stronger influence [63]. Since BRTs do not provide significance tests, but only variables’ relative contribution to the model’s predictive power, those that were disproportionately represented in the trees (i.e. above the threshold of 100%/n variables) were considered highly influential [10, 18]. Partial dependency plots with 95% confidence intervals obtained from 1000 bootstrap replicates were used to examine the relationships between the response and the most influential predictor variables, while keeping all other predictors at their mean [10, 65]. The presence of interactions between influential variables was also examined and plotted following Elith et al. [59].

## Results

### Spatial variability in reef status and structure

**Fig 2:**
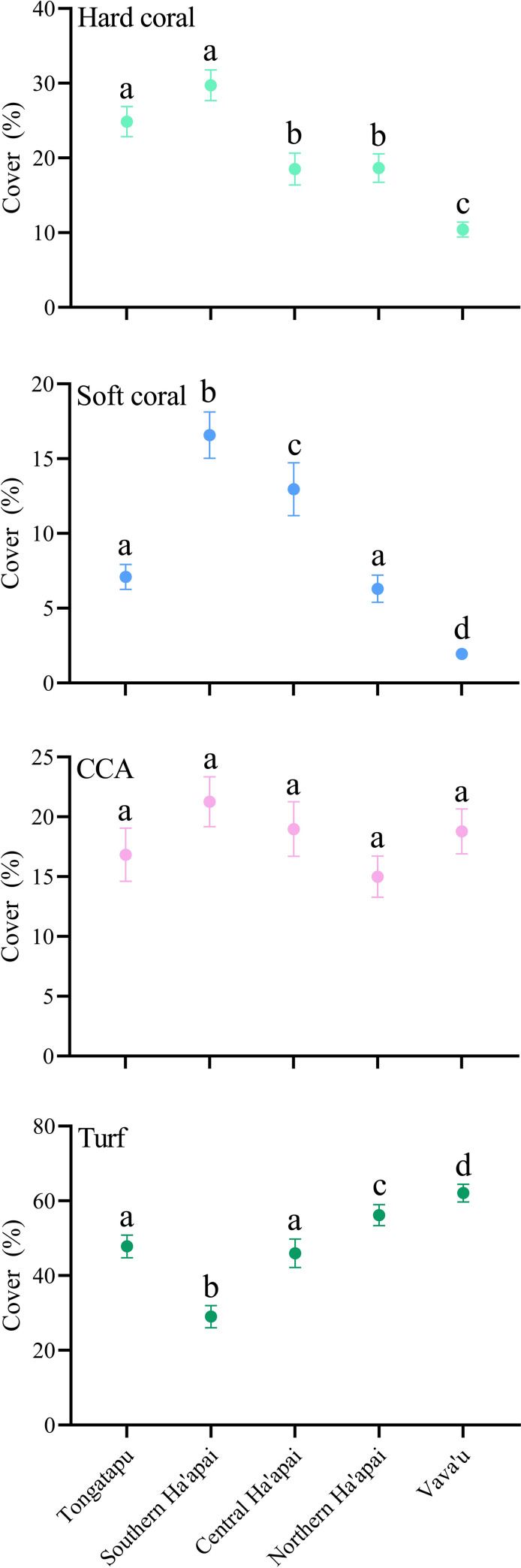
Patterns in benthic cover across the three main island groups of Tonga, arranged from south to north. The Ha’apai group was split into southern, central and northern Ha’apai due to high latitudinal variation within the group. Values represent mean ± 95% confidence intervals. Letters denote significant groupings based on Tukey’s post hoc comparisons.

Overall, mean hard coral cover across the 343 sites in Tonga for which benthic data were available was 18% (+/- .625 SE). However, mean hard coral cover in Vava’u was less than half that of the other island groups (Fig 2; S2 Table in S1 File), and this pattern was also similar for soft coral. Many sites in Vava’u had 0% live coral cover, particularly around the inner islands where turfing algae and bare matrix were dominant, and coral cover generally increased towards the outer islands. While a few sheltered, inner island sites in Vava’u had hard coral cover >30%, this was dominated by two species from a single genus (*Porites rus* and *Porites cylindrica*). Coral reef communities around many of the shallow, fringing reefs in the inner, enclosed areas of Vava’u appeared to be characterized by little or no hard or soft coral cover. Sites near the mouths of the two large estuarine lagoons in Vava’u often had 0% live coral cover and large numbers of *Diadema sp*. sea urchins, which appeared to be destroying the reef matrix. Coral cover in the Ha’apai island group increased gradually from north to south, with exposed sites in the southern islands (e.g. Nomuka, Mango and Fonoi) having the greatest cover of the sites assessed. Likewise, sites along the outer western islands (e.g. Ofalanga, Mounga’one, Kotu and Muitoa) generally had greater coral cover than the sheltered sites along the margins of the ribbon islands in north eastern Ha’apai (e.g. Foa and Lifuka). There was widespread evidence of damage from multiple cyclones and bleaching events along the western, sheltered edges of the north-east ribbon islands (Ha’ano, Foa, Lifuka and Uoleva). With the exception of southern Ha’apai, live coral cover in Tongatapu and near the capital Nuku’alofa was consistently greater than elsewhere in Tonga. Most sites within the central bay, and even fringing reefs adjacent to the city centre, had moderate coral cover. As in Ha’apai, there was evidence of bleaching damage along back reefs of the north-eastern ribbons from Tao to Nuku island. As in Vava’u, near the mouth of the Fanga’uta lagoon, Tongatapu, there were large numbers of *Diadema sp*. sea urchins and very low (often 0%) live coral cover.

Patterns of CCA cover did not vary significantly between island groups in Tonga. Conversely, there were substantial differences in the mean cover of turfing algae throughout Tonga. These patterns were largely the inverse of live coral cover, with the greatest cover in Vava’u and lowest in Southern Ha’apai.

**Fig 3:**
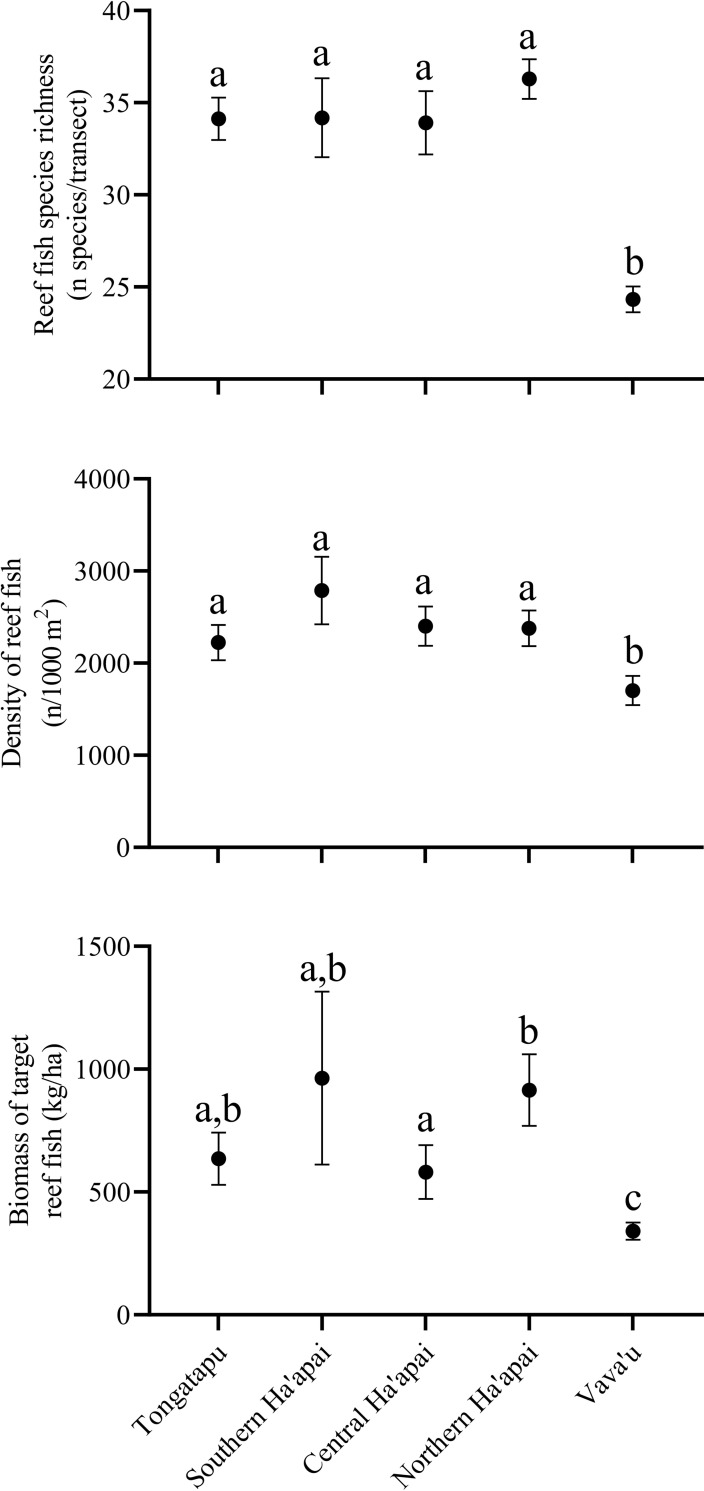
Patterns of reef fish species richness, density and target biomass across Tonga’s three main island groups arranged from south to north. The Ha’apai group was split into southern, central and northern Ha’apai due to high latitudinal variation within the group. Values represent mean ± 95% confidence intervals. Letters denote significant groupings based on Tukey’s post-hoc comparisons.

A total of 510 individual reef fish species (S7 Table in S1 File) were identified throughout the surveys, and both species richness and density varied significantly between island groups (Fig 3; S3 Table in S1 File). However, post hoc analysis revealed that only the Vava’u island group clustered separately for both species richness and density and that there was little variation between the other island groups. The overall mean biomass of target species also varied between regions, with Vava’u having the lowest standing biomass of the sites assessed. However, there was also high variability in biomass within the other island groups, with southern and northern Ha’apai having the greatest standing biomass (963± 183 SE kg/ha and 914 ± 73 SE kg/ha respectively).

**Fig 4:**
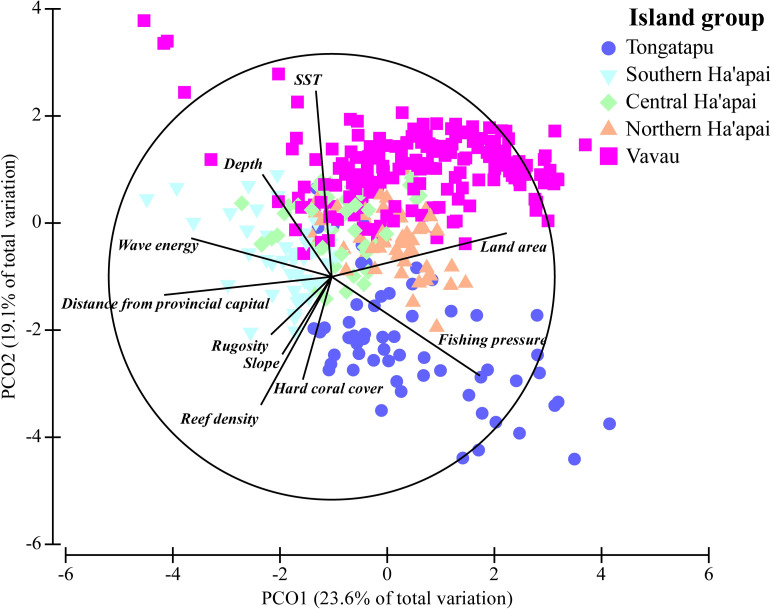
Principal component ordination of the distribution of socio-environmental variables across Tonga’s island groups. PCO was run on normalized data using Euclidean distances.

Principal component ordination demonstrated clustering of island groups for the socio-environmental predictor variables, although there was also substantial overlap between groups and variability within groups (Fig 4). Fishing pressure was substantially greater in Tongatapu than elsewhere. Both reef density and hard coral cover were greatest in Southern Ha’apai and Tongatapu. Sites in Southern Ha’apai were also the most remote as measured by distance from the provincial capital, and had the greatest wave energy. The largest differences between Vava’u and elsewhere in Tonga were the warmer SST (by 2°C) and the low density of reef habitat.

### Predicting reef condition

BRT models performed well for all seven models, with explained deviance between 29.55% and 68.35%. Spatial autocorrelation was also low, with a maximum Moran’s I values of 0.07. For each of the seven variables, figures are included that describe: i) the spatial distribution of observed values across all surveyed sites; ii) the relative influence of each predictor variable; iii) the relationships of the most influential predictors; and, iv) significant interactions between influential variables.

#### Benthic variables

**Fig 5:**
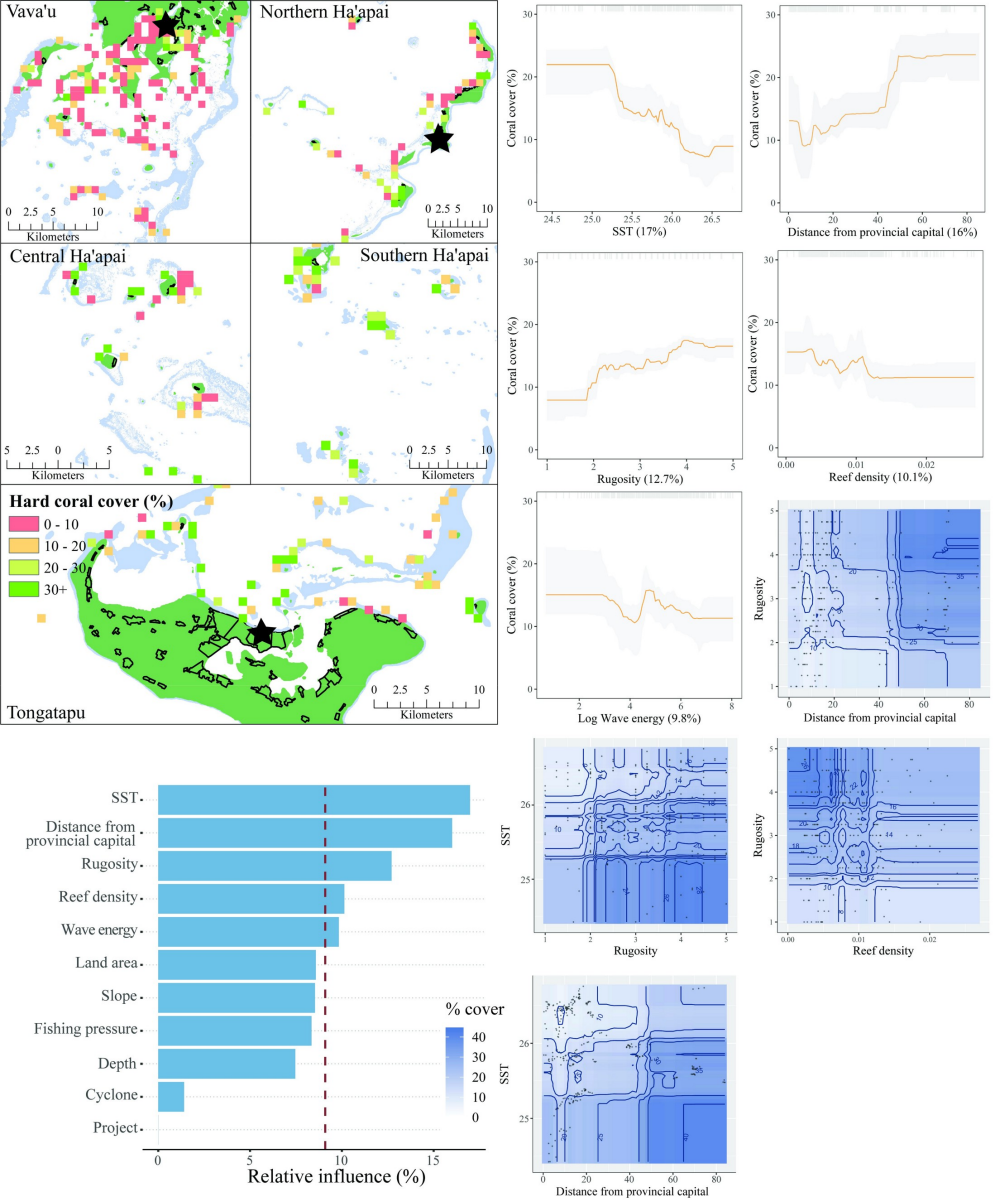
Hard coral cover. **Top left:** Map of hard coral cover at sites sampled across Tonga. Light blue represents reef, green land, and black outlines villages. Each provincial capital is marked by a black star. **Bottom left:** Relative influence of the 11 predictor variables included in the Boosted Regression Tree. The dashed vertical line represents a reference point of relative influence that would be expected if all predictors were equally influential. **Top right:** Partial dependency plots with 95% confidence intervals for the most influential variables predicting hard coral cover. The plots show the effect of each predictor on the repsonse while all other variables were at their mean values. Relative influence of each predictor is reported in parentheses. Grey tick marks across the top of each plot indicate observed data points. **Bottom right:** Plots of the strongest pairwise interactions between influential variables. Contour lines indicate model predictions and points represent observed data. Units are as follows: SST−^O^Celcius, distance from provincial capital–km, rugosity– 1–5, reef density–km^2^, log wave energy–Joules per m^2^.

The five most influential predictors of hard coral cover in Tonga were SST, distance from the provincial capital, habitat rugosity, reef density, and wave energy (Fig 5). Negative relationships were observed between hard coral cover and each of SST, reef density, and wave energy. Positive relationships occurred between live hard coral cover and both distance from the provincial capital and habitat rugosity. Four interactions between influential variables were present. Taken together, the partial plots and interactions predicted that hard coral cover was greatest in areas far from the provincial capitals, with high rugosity, lower SST, and low reef density. The model explained 37% of the cross-validated deviance.

**Fig 6:**
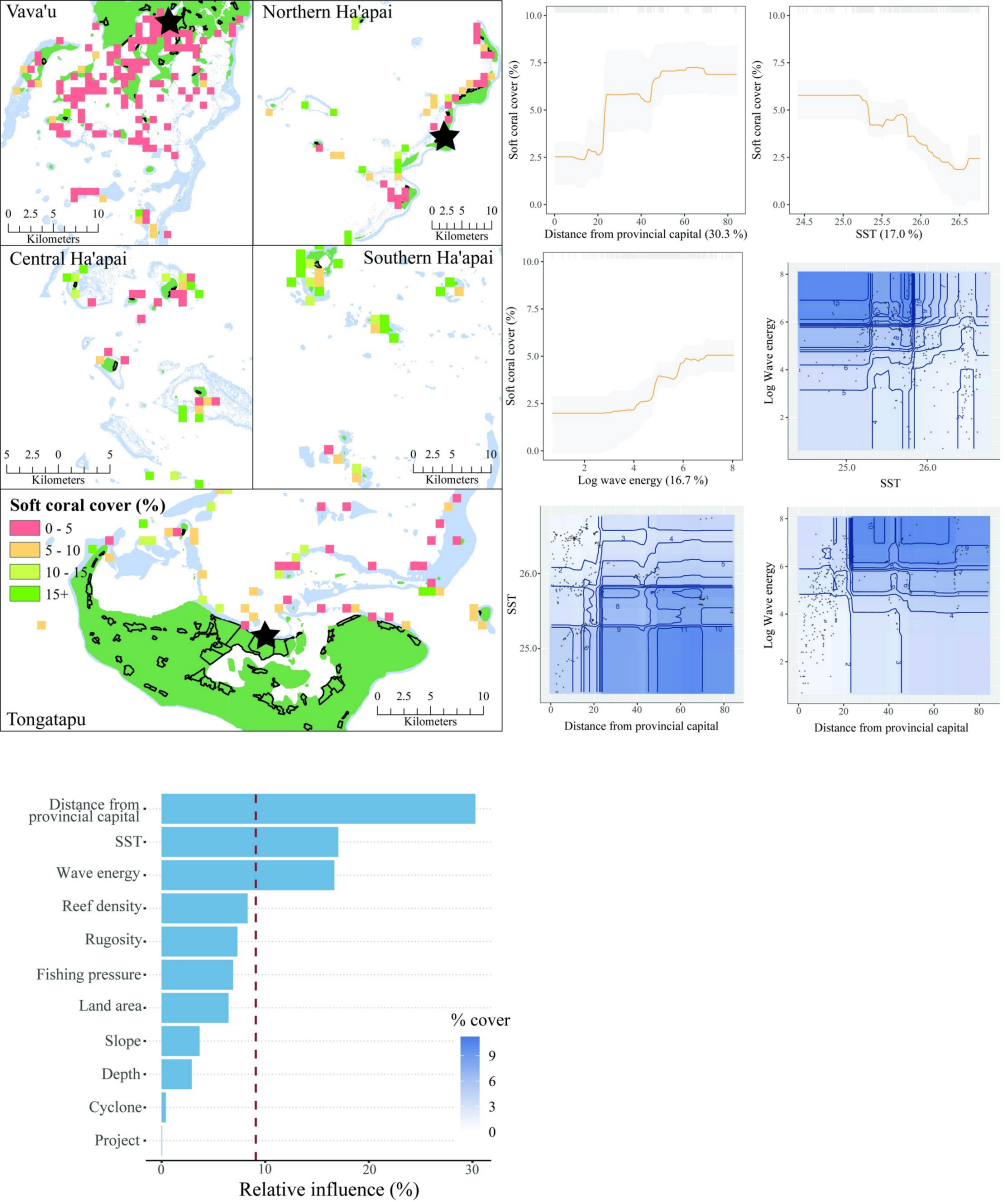
Soft coral cover. **Top left:** Map of soft coral cover at sites sampled across Tonga. Light blue represents reef, green land, and black outlines villages. Each provincial capital is marked by a black star. **Bottom left:** Relative influence of the 11 predictor variables included in the Boosted Regression Tree. The dashed vertical line represents a reference point of relative influence that would be expected if all predictors were equally influential. **Top right:** Partial dependency plots with 95% confidence intervals for the most influential variables predicting soft coral cover. The plots show the effect of each predictor on the repsonse while all other variables were at their mean values. Relative influence of each predictor is reported in parentheses. Grey tick marks across the top of each plot indicate observed data points. **Bottom right:** Plots of the strongest pairwise interactions between influential variables. Contour lines indicate model predictions and points represent observed data. Units are as follows: distance from provincial capital–km, SST−^O^Celcius, log wave energy–Joules per m^2^.

The three most influential predictors of soft coral cover in Tonga were SST, distance from the provincial capital, and wave energy (Fig 6). There was a strong positive relationship between soft coral cover and distance from the provincial capital. As with hard coral, a negative relationship was observed between soft coral cover and SST. Unlike hard coral cover, soft coral cover was positively associated with increased wave energy. There were interactions between all three influential variables. Taken together, the partial plots and interactions indicated that soft coral cover was greatest at remote sites with high wave energy and cooler temperatures. The model explained 58% of the cross-validated deviance.

**Fig 7:**
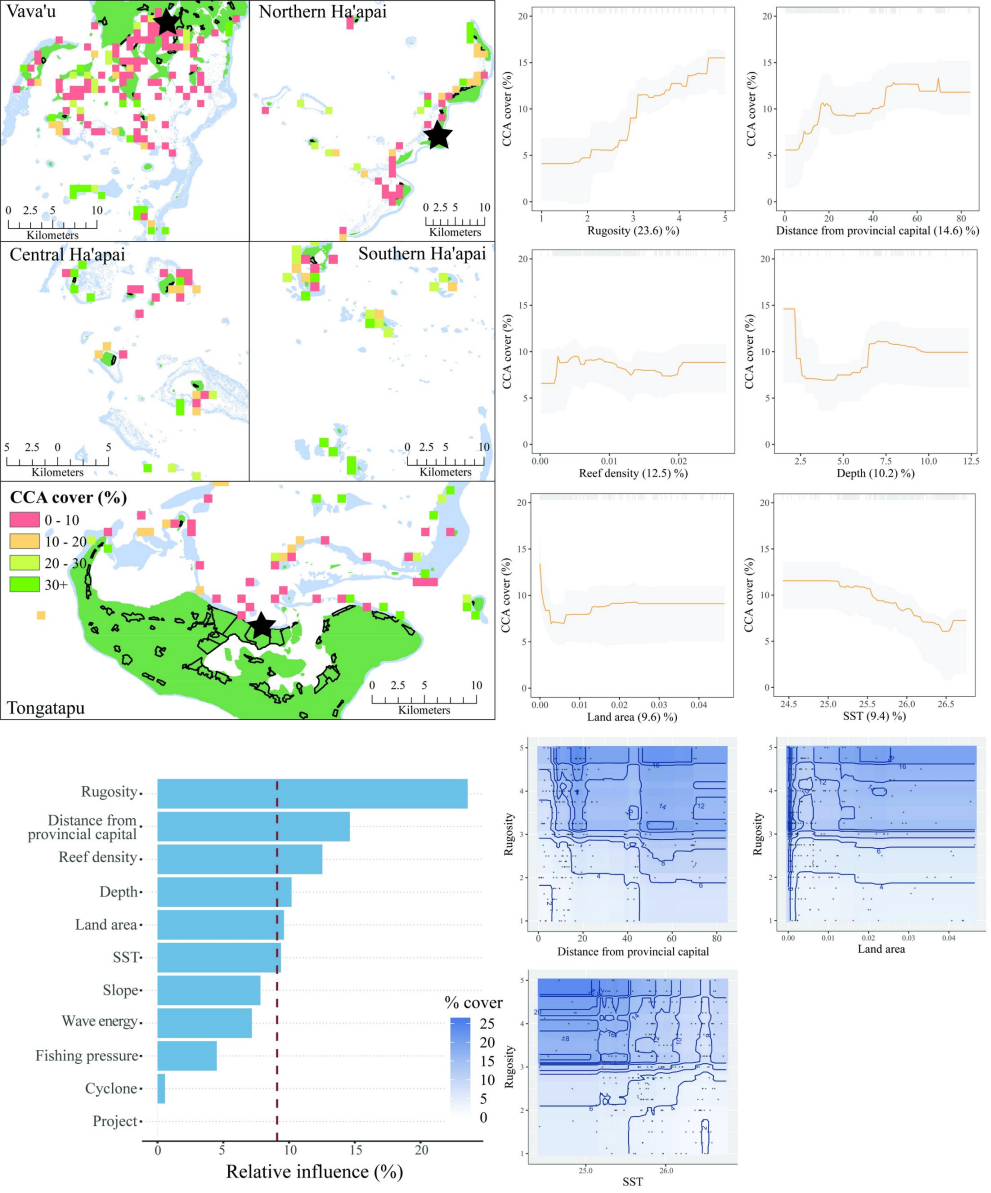
CCA cover. **Top left:** Map of CCA cover at sites sampled across Tonga. Light blue represents reef, green land, and black outlines villages. Each provincial capital is marked by a black star. **Bottom left:** Relative influence of the 11 predictor variables included in the Boosted Regression Tree. The dashed vertical line represents a reference point of relative influence that would be expected if all predictors were equally influential. **Top right:** Partial dependency plots with 95% confidence intervals for the most influential variables predicting CCA cover. The plots show the effect of each predictor on the repsonse while all other variables were at their mean values. Relative influence of each predictor is reported in parentheses. Grey tick marks across the top of each plot indicate observed data points. **Bottom right:** Plots of the strongest pairwise interactions between influential variables. Contour lines indicate model predictions and points represent observed data. Units are as follows: rugosity– 1:5, distance from provincial capital–km, reef density–km^2^, depth–meters, land area–km^2^, SST−^O^Celcius.

The six most influential predictors of CCA in Tonga were habitat rugosity, distance from the provincial capital, reef density, depth, land area, and SST (Fig 7). The two most influential predictors, rugosity and distance from the provincial capital, both had strong positive relationships with CCA. While reef density was influential at predicting CCA cover, there was not a clear pattern in the direction of the relationship. CCA cover was lowest around five meters depth, and increased towards shallower and deeper water. Model predictions suggest that CCA cover has a strong negative relationship with levels of terrestrial influence, but only at very low values of this predictor (<0.05km^2^). This relationship breaks down with greater terrestrial influence. As with coral cover, SST was negatively associated with the percent cover of CCA. Three variables had interactions with habitat rugosity (distance from provincial capital, land area, and SST), all of which predicted greater cover of CCA at higher rugosity levels. The model explained 51% of the cross-validated deviance.

**Fig 8:**
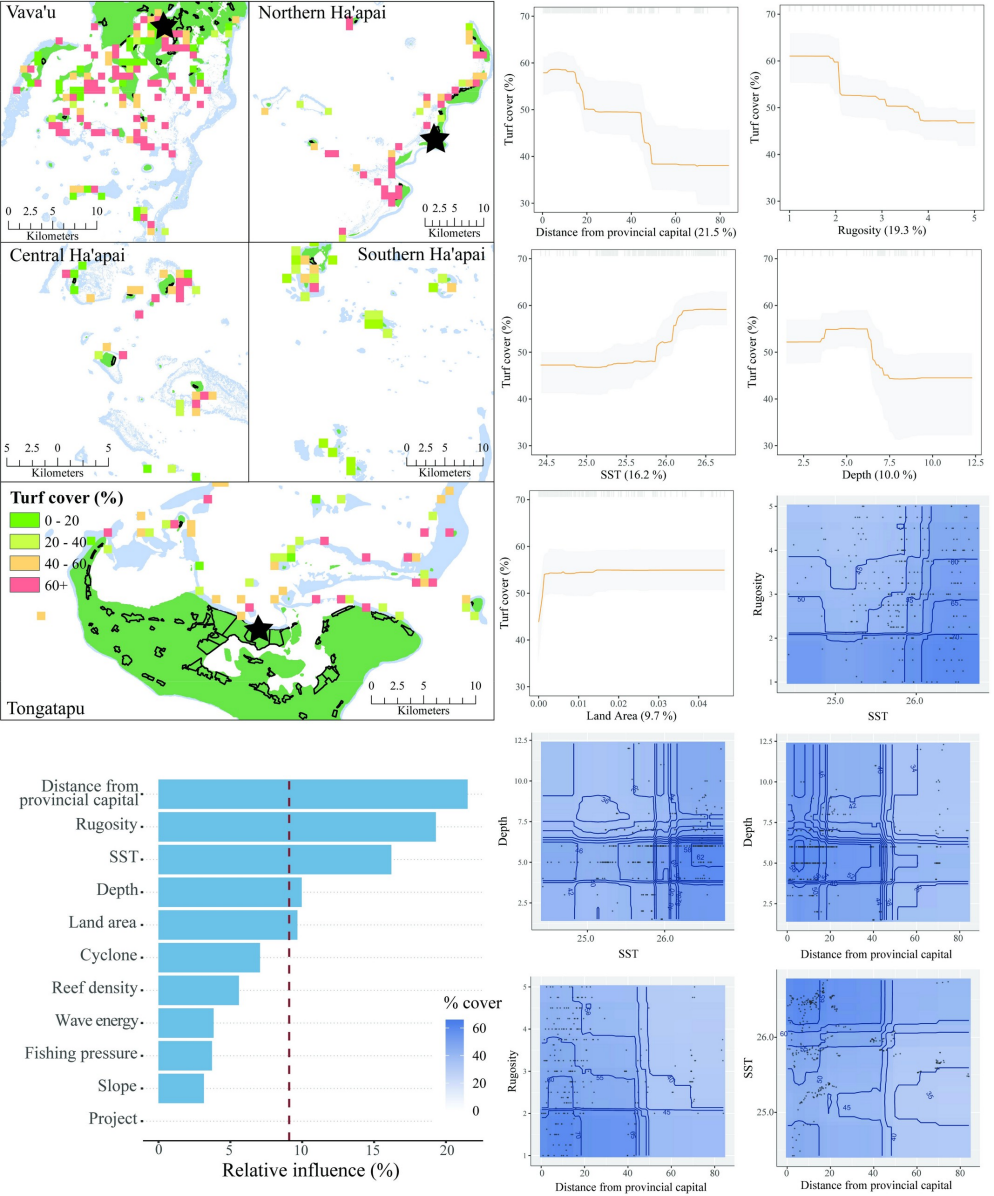
Turf algae cover. **Top left:** Map of turf cover at sites sampled across Tonga. Note the color scale here is the inverse of other variables. Light blue represents reef, green land, and black outlines villages. Each provincial capital is marked by a black star. **Bottom left:** Relative influence of the 11 predictor variables included in the Boosted Regression Tree. The dashed vertical line represents a reference point of relative influence that would be expected if all predictors were equally influential. **Top right:** Partial dependency plots with 95% confidence intervals for the most influential variables predicting turf algae cover. The plots show the effect of each predictor on the repsonse while all other variables were at their mean values. Relative influence of each predictor is reported in parentheses. Grey tick marks across the top of each plot indicate observed data points. **Bottom right:** Plots of the strongest pairwise interactions between influential variables. Contour lines indicate model predictions and points represent observed data. Units are as follows: distance from provincial capital–km, rugosity– 1:5, SST−^O^Celcius, depth–meters, land area–km^2^.

The five most influential predictors of turf algae cover in Tonga were distance from the provincial capital, habitat rugosity, SST, depth, and land area (Fig 8). The relationship between turf algae and the predictor variables was also the opposite compared to other benthic variables. Turf algae coverage was greatest close to each provincial capital and declined with increasing distance from human influence. Likewise, lower levels of rugosity had the greatest cover of turfing algae. SST and land area were both positively associated with turf cover, although for land area, as with CCA, the relationship was greatest at low levels of land area (<0.05 km^2^) and plateaued at levels greater than this. For turf algae, depth displayed the opposite relationship to that for tuCCA, with greatest cover at 5 meters depth and lower levels in shallower and deeper water. There were five interactions that predicted turf cover. Taken together, the results suggested that turf algae was most dominant in shallow, low-complexity reefs that were close to human influence and in warmer waters. The model explained 54% of the cross-validated deviance.

#### Fish variables

**Fig 9:**
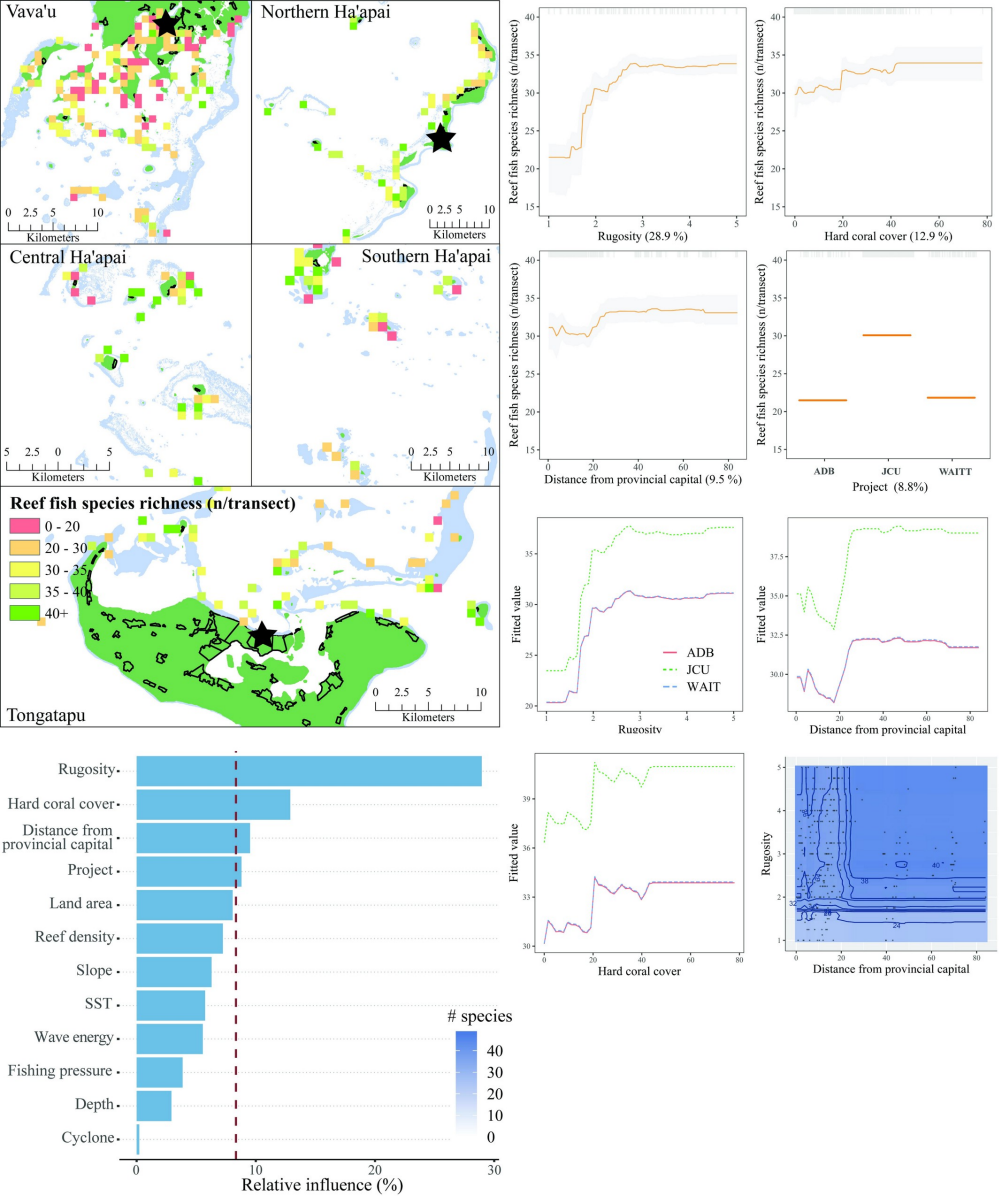
Reef fish species richness. **Top left:** Map of reef fish species richness at sites sampled across Tonga. Light blue represents reef, green land, and black outlines villages. Each provincial capital is marked by a black star. **Bottom left:** Relative influence of the 12 predictor variables included in the Boosted Regression Tree. The dashed vertical line represents a reference point of relative influence that would be expected if all predictors were equally influential. **Top right:** Partial dependency plots with 95% confidence intervals for the most influential variables predicting reef fish species richness. The plots show the effect of each predictor on the repsonse while all other variables were at their mean values. Relative influence of each predictor is reported in parentheses. Grey tick marks across the top of each plot indicate observed data points. **Bottom right:** Plots of the strongest pairwise interactions between influential variables. Contour lines indicate model predictions and points represent observed data. Units are as follows: rugosity– 1:5, hard coral cover—%, distance from provincial capital–km.

The four most influential predictors of reef fish species richness were habitat rugosity, hard coral cover, distance from provincial capital, and project (Fig 9). Species richness increased substantially with rugosity values between one and three, but plateaued above three. The relationships between reef fish species richness and both hard coral cover and distance from provincial capital were similar and positive, with increased richness up to ~40% live coral cover and 30 km from the capital, before it also plateaued. Lastly, surveys conducted under the James Cook University led project consistently recorded a greater number of species than the other projects. Interactions were fitted between project and all three other influential predictor variables, which show that the same pattern was evident across projects despite this inconsistency. There was also an interaction between distance from provincial capital and rugosity, with complex reefs in remote areas having greater species richness. The model explained 68% of the cross-validated deviance.

**Fig 10:**
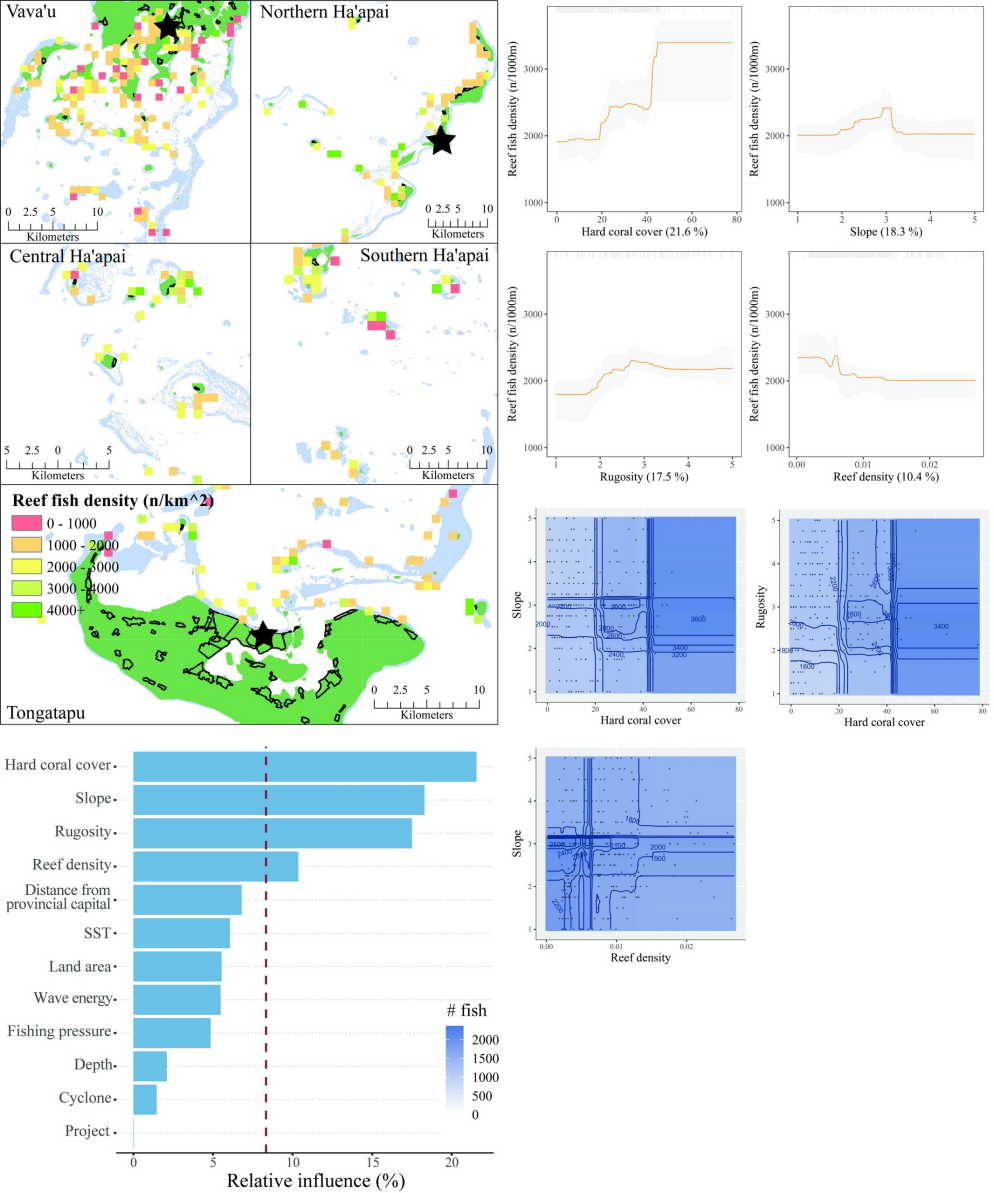
Reef fish density. **Top left:** Map of reef fish density at sites sampled across Tonga. Light blue represents reef, green land, and black outlines villages. Each provincial capital is marked by a black star. **Bottom left:** Relative influence of the 12 predictor variables included in the Boosted Regression Tree. The dashed vertical line represents a reference point of relative influence that would be expected if all predictors were equally influential. **Top right:** Partial dependency plots with 95% confidence intervals for the most influential variables predicting reef fish density. The plots show the effect of each predictor on the repsonse while all other variables were at their mean values. Relative influence of each predictor is reported in parentheses. Grey tick marks across the top of each plot indicate observed data points. **Bottom right:** Plots of the strongest pairwise interactions between influential variables. Contour lines indicate model predictions and points represent observed data. Units are as follows: hard coral cover—%, slope– 1:5, rugosity– 1:5, reef density–km^2^.

The four most influential predictors of reef fish density were hard coral cover, reef slope, habitat rugosity, and reef density (Fig 10). Reef fish density increased substantially at both 20% and 40% hard coral cover. Density was also greatest at mid-levels of reef slope, which correspond to ~45^o^. More complex reefs, with rugosity scores above 3, also had greater densities of reef fish. The relationship between reef fish density and reef density was slightly negative. Three interactions between influential variables were present. Taken together, the partial plots and interactions predicted that reef fish density was greatest with high coral cover and mid-sloped reefs with relatively low reef density nearby. The model explained 30% of the cross-validated deviance.

**Fig 11:**
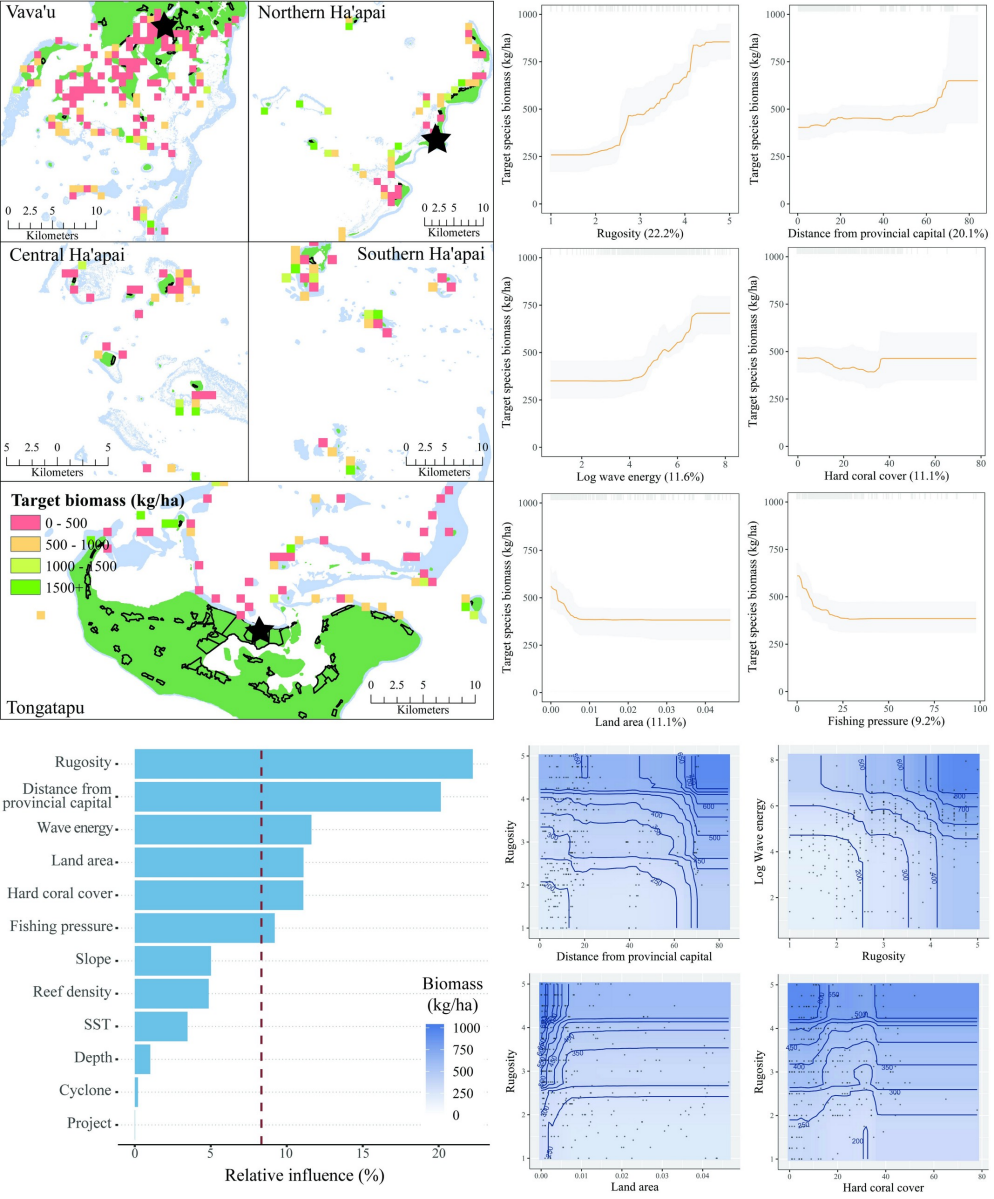
Target fish biomass. **Top left:** Map of target fish biomass at sites sampled across Tonga. Light blue represents reef, green land, and black outlines villages. Each provincial capital is marked by a black star. **Bottom left:** Relative influence of the 12 predictor variables included in the Boosted Regression Tree. The dashed vertical line represents a reference point of relative influence that would be expected if all predictors were equally influential. **Top right:** Partial dependency plots with 95% confidence intervals for the most influential variables target fish biomass. The plots show the effect of each predictor on the repsonse while all other variables were at their mean values. Relative influence of each predictor is reported in parentheses. Grey tick marks across the top of each plot indicate observed data points. **Bottom right:** Plots of the strongest pairwise interactions between influential variables. Contour lines indicate model predictions and points represent observed data. Units are as follows: rugosity– 1:5, distance from provincial capital–km, log wave energy–Joules per m^2^, hard coral cover—%, land area–km^2^, fishing pressure–weighted abundance of fishers scaled to 100.

The six most influential predictors of target species biomass were habitat rugosity, distance from the provincial capital, wave energy, hard coral cover, land area, and fishing pressure (Fig 11). Biomass increased consistently with increasing rugosity. The relationship between biomass and distance from the capital of each island group was positive, although the greatest increase was at distance greater than 60 km away. The relationship between biomass and wave energy was also positive. While hard coral cover was an influential predictor of biomass, the relationship was unclear. Both land area and fishing pressure displayed similar patterns in their relationship with biomass, with strong declines in biomass at low levels of the predictors (land area <0.05 and fishing pressure <25), followed by plateaus. Four interactions between influential variables were present. Taken together, the partial plots and interactions predicted that target fish biomass was greatest in high wave energy, structurally complex reef, far from human pressures. The model explained 46% of the cross-validated deviance.

## Discussion

We provide the first national description of the status of Tonga’s coral reef ecosystem and reef fish fishery. Clear differences exist in the structure of coral reef ecosystems along a latitudinal gradient that corresponds to the different island groups of the country. These differences appear to be well explained by a combination of both natural biophysical variables (e.g. habitat rugosity, wave energy), and anthropogenic influences (e.g. distance from provincial capital, fishing pressure). Overall live coral cover was low (18%) but comparable to other regions throughout the Pacific affected by anthropogenic pressures [7]. Reef fish species richness for Tonga fell within the expected bounds for this part of the Pacific and these types of surveys [66]. Overall mean reef fish biomass values suggest that Tonga’s reef fish fishery can be classified as moderately to heavily exploited, with 64% of sites having less than 500 kg/ha [24, 25, 67]. In the sections that follow we: i) discuss the major relationships between socio-environmental variables and key metrics of reef condition, including several caveats to our findings; and ii) provide further details of the observed patterns within each island group.

### Major relationships between predictive variables and reef condition

The strongest and most common two predictors of reef structure in Tonga were habitat rugosity and distance from the provincial capital. Habitat rugosity is a well-established driver of reef community structure, and is linked to both natural processes and anthropogenic disturbances [15, 68]. Given the near ubiquitous effect of distance from the capital, our findings also suggest that Tonga’s human influence is having a clear and strong impact on the structure of their reefs. The authors are unaware of any natural biophysical variables that correlate strongly with distance from each island group’s main city. However, distance from human population centres is not in itself a disturbance, but only a proxy for the many types of influence that humans can cause. While the clearest is likely to be fishing pressure (in addition to variability not accounted for in the fishing pressure metric), other disturbances could also include pollution or development. Importantly, other metrics related to human influence have also been developed, most notable the Human Gravity metric of Cinner et al. [27], which might be better at predicting patterns of human impact on reef structure. However, for the current analysis, these variables were at too coarse a resolution to be employed.

The negative effects of increased SST on scleractinian corals is well documented [3, 69]. However, coral bleaching is primarily associated with heat stress events, including high variability in SST, or sustained temperatures above the thermal tolerance of coral species (e.g. degree heating weeks [DHW]). Due to the coarse resolution (5 km) of the National Oceanic and Atmospheric Association (NOAA) Coral Reef Watch (CRW) layers for SST variability and heat stress events when compared to the resolution of this analysis, we were unable to include SST variability or DHW in the set of predictor variables (S1 File). Despite this caveat, many reefs in northern Ha’apai and Vava’u did display signs of recent bleaching events. In several instances bleaching of entire reefs had clearly occurred within the past five years, indicated by retained complexity of dead corals. The two-degree difference in mean SST between Tongatapu and Vava’u provides a potential mechanism by which coral cover in Vava’u was reduced, because corals in this island group could be living closer to their thermal bleaching threshold. However, acute thermal anomalies have a greater influence on coral assemblages than long-term means and coral bleaching thresholds are relative to local thermal histories [3]. Therefore, the negative relationship between SST and live coral cover could also be related to differential acute heat exposure among locations due to oceanographic factors or local weather conditions mediating heat stress, or to additional factors that are collinear with SST. For example, the sheltered geography of Vava’u might limit flushing by cooler, oceanic waters, so that when extreme temperature events do occur, they could be more pronounced in duration and extent. It is also possible that local weather conditions (e.g. wind, cloud cover, and rain) led to lower thermal stress in Tongatapu during periods of thermal stress, as was the case on the Great Barrier Reef in 2016 [3]. Given the lack of previous data, it is not possible to accurately determine the frequency or severity of mass bleaching events in Tonga, and further research is necessary to separate the effects of large-scale versus local oceanic conditions.

Patterns of reef fish diversity and density in Tonga appear to be associated primarily with natural biophysical variables, such as reef complexity (i.e. rugosity), slope, and hard coral cover. The importance of both structural complexity and live coral cover as influences on coral reef fish communities is well recognized (e.g. 0; 15) and, although often collinear, it is nonetheless important to distinguish the two. While scleractinian corals are the dominant habitat-forming organisms on reefs, larger-scale structural complexity can be more strongly associated with long-term patterns of reef accretion than the immediate presence of live coral, as well as habitat-forming organisms in addition to corals [70,71]. While project was also an important driver at predicting reef fish species richness, one of the benefits of BRT analysis and partial dependency plots is the ability to account for issues such as these. The relationships between all other predictor variables and reef fish species richness were therefore considered within this context and examined while controlling for variation in project methodologies.

Based on previous calculations of global baselines and benchmarks for fish biomass [24, 25, 67], our results suggest that Tonga’s reef fish fishery can be classified as heavily exploited in Vava’u, moderately exploited in Tongatapu and central Ha’apai, and with lower levels of exploitation in southern and northern Ha’apai. Target species biomass responded strongly to a combination of biophysical and local anthropogenic variables. While there was a sharp decline in biomass with increasing fishing pressure at low levels, the effects plateaued at higher levels of fishing. This also corresponds to the greatest increase in biomass at sites furthest from the provincial capitals. Together these findings support the hypothesis laid out previously from studies across large gradients of human population and fishing density that the highest absolute losses in reef fish can occur with relatively low fishing pressure [25, 72, 73]. However, differences in population density and fishing pressure do not explain why target species biomass in Vava’u was lower than Tongatapu, where human impacts are greatest. Instead, given the importance of both biophysical and anthropogenic variables at predicting biomass in our data, similarities in biomass values between Tongatapu and Vava’u might be best explained by overfishing in Tongatapu and by poor quality habitat (e.g. low rugosity, coral cover and wave energy) in Vava’u.

### Considerations within each island group

#### Vava’u

The most prominent findings from these data are the poor results for Vava’u across all metrics of reef condition. Coral cover in Vava’u was exceptionally low (mean 10.4%), but even this was buffered by several lagoonal sites with high cover of *Porites rus* and *Porites cylindrica*. With these sites removed, mean coral cover for the rest of Vava’u was closer to 5%. Likewise, richness, density and biomass estimates for Vava’u were all lower than other island groups. The geography of Vava’u is unique and very different to reefs in Ha’apai and Tongatapu that are more typical of Pacific reefs. The reefs of Vava’u are generally sheltered, narrow fringing reefs below limestone cliffs and adjacent to deep (60–100 m) water. Most reefs are likely to have very little current flow and are sheltered from the open ocean and prevailing weather conditions. Reefs in Vava’u might therefore be more susceptible to impacts from both coral bleaching and local pollution. When reefs are subjected to heatwaves, coral bleaching in more open areas could be limited by flushing from cool oceanic waters, while the geography of Vava’u would limit flushing and result in pockets of warm water persisting for much longer. Likewise, pollutants from local sources are unlikely to wash away readily given the topography of the islands and instead might persist at greater concentrations. However, limited data are available on current regimes around Vava’u to investigate this hypothesis.

#### Ha’apai

While reefs in southern Ha’apai were generally in the best condition of those assessed, there was also extensive evidence of recent bleaching along the western edge of the northern Ha’apai ribbon reefs. As with Vava’u, part of this problem could be associated with prevailing wind, wave and current conditions, which generally move from east to west. Many of the sites in northern Ha’apai are sheltered from the east by the main islands and therefore could also trap pockets of warm water, exacerbating bleaching at a local scale. Conversely, the reefs of Southern Ha’apai are much more exposed, which might therefore promote flushing by prevailing winds, waves and currents.

An additional point worth noting is that there are increasing numbers of fishers from Tongatapu travelling to Southern Ha’apai to fish, as well as fishers from Ha’apai transporting their catch to Faua Wharf in Tongatapu (personal observation). Both of these factors could potentially confound the influence of local fishing pressure and distance from the provincial capital on target biomass. However, given the strength of these variables, it is still clear that fishing activities within island groups are strong predictors of fish biomass.

#### Tongatapu

The coral reefs around Tongatapu have experienced the greatest human pressures in Tonga, with 70% of the country’s population on this island. The Fanga’uta lagoon is highly polluted [31] and flows directly onto reefs in the Tonga channel. Likewise, the number of fishers in Tongatapu is equal to that in Ha’apai and Vava’u combined [74]. Despite this, the reefs in Tongatapu were overall in better condition than anticipated. Coral cover within the main bay was higher than elsewhere assessed and reef fish richness and density were moderate. These results could be due to the cooler waters in Tongatapu, which might buffer against the large bleaching events which appear to have impacted Vava’u and northern Ha’apai. Only target biomass was low, which is expected, given the clear relationship between human influence and biomass [27]. As explained previously, the similarities in fish biomass between Vava’u and Tongatapu might therefore be best explained by overfishing in Tongatapu and reef condition in Vava’u.

### Additional considerations

Several sites of note were also identified with very poor coral cover (0%) in both Tongatapu and Vava’u, which warrant further investigation. In Tongatapu these were near the mouth of the Fanga’uta lagoon and in Vava’u in many of the inner island areas, particularly near the causeways. At these sites there were often no living corals and instead high densities of *Diadema sp*. sea urchins. These appeared to be scraping away the reef matrix extensively and inadvertently destroying any recruiting corals. It is possible that pollution from Tonga’s lagoonal areas might be causing outbreaks of *Diadema sp*. sea urchins. We recommend this possibility as a critical area for further investigation in Tonga. An additional caveat of this study was the inability to attribute reef condition to cyclone damage, which is known to affect reefs on a large scale. One possible explanation is Tonga’s small geographic extent and the small geographic extent of our surveys compared to the scale of most cyclones. Damaging wind levels in excess of 50 knots frequently affect most of the country during a single cyclone event, and this large proportional coverage may mask patterns of damage to Tonga’s reefs, given that most of the country is affected at the same time. The fine scale of surveys may therefore have been unable to detect impacts at larger spatial scales.

While fisheries management in Tonga has been historically open access, in recent years this has changed with the implementation of the Special Management Area (SMA) program. This locally driven initiative has now grown to include over 50 communities that each have at least one no-take marine protected area as well as an exclusive access zone [75]. Previous studies using statistical matching have demonstrated positive impacts for reef fish biomass, density and species richness for the seven oldest community-based no-take zones in Tonga [49]. While the present study did not test management status per se, differences in fishing pressure due to management were included within the fishing pressure metric, and therefore the positive impacts of management were incorporated into the analysis.

## Conclusions

Our data and analysis deliver critical baseline ecological information for Tonga’s coral reefs that will both aid ongoing management and research and enable accurate reporting to local and international agencies. For example, future reports on Tonga’s coral reefs need no longer classify them as ‘data deficient’, and some degree of accountability should now be expected from governments regarding effective policies and management. There is also now a great deal more information available to support the SMA program, with data from these projects already being used to examine the impact of existing and potential new configurations of no-take reserves [47, 49]. These data have also been compiled into a large national report, available in both English and Tongan, to increase public awareness about reef status and the effects of management [50]. Lastly, we anticipate that this extensive data set can be used as a benchmark for ongoing monitoring and future impact evaluation studies in Tonga.

## Supporting information

S1 File (DOCX)

## References

[1] Bellwood DR, Pratchett MS, Morrison TH, Gurney GG, Hughes TP, Álvarez-Romero JG, et al Coral reef conservation in the Anthropocene: Confronting spatial mismatches and prioritizing functions. Biol Conserv. 2019;236:604–15.

[2] Morrison TH, Hughes TP, Adger WN, Brown K, Barnett J, Lemos MC. Save reefs to rescue all ecosystems. Nature Publishing Group; 2019 10.1038/d41586-019-02737-8 31534250

[3] Hughes TP, Kerry JT, Álvarez-Noriega M, Álvarez-Romero JG, Anderson KD, Baird AH, et al Global warming and recurrent mass bleaching of corals. Nature. 2017;543(7645):373–7. 10.1038/nature21707 28300113

[4] Hughes TP, van de Leemput IA, Morrison TH, Kleypas J, van Nes EH, Barnes ML, et al Coral reefs in the Anthropocene. Nature. 2017;546(7656):82–90. 10.1038/nature22901 28569801

[5] Stone K, Fenner D, LeBlanc D, Vaisey B, Purcell I, Eliason B. Tonga [Internet] Second Edi. World Seas: an Environmental Evaluation. Elsevier Ltd; 2019 661–678 p. Available from: https://linkinghub.elsevier.com/retrieve/pii/B9780081008539000385

[6] Vercelloni J, Liquet B, Kennedy E V, González‐Rivero M, Caley MJ, Peterson EE, et al Forecasting intensifying disturbance effects on coral reefs. Glob Chang Biol. 2020;26(5):2785–97. 10.1111/gcb.15059 32115808

[7] Chin A, DeLoma T, Reytar K, Planes S, Gerhardt K, Clua E, et al Status of Coral Reefs of the Pacific and Outlook: 2011. Publishers Global Coral Reef Monitoring Network. 260 P. 2011.

[8] Cinner JE, Huchery C, MacNeil MA, Graham NAJ, McClanahan TR, Maina J, et al Bright spots among the world’s coral reefs. Nature. 2016;535(7612):416–9. 10.1038/nature18607 27309809

[9] Darling ES, McClanahan TR, Maina J, Gurney GG, Graham NAJ, Januchowski-Hartley F, et al Social–environmental drivers inform strategic management of coral reefs in the Anthropocene. Nat Ecol Evol. 2019;3(9):1341–50. 10.1038/s41559-019-0953-8 31406279

[10] Jouffray J-B, Wedding LM, Norström A V., Donovan MK, Williams GJ, Crowder LB, et al Parsing human and biophysical drivers of coral reef regimes. Proc R Soc B Biol Sci [Internet]. 2019;286(1896):20182544 Available from: 10.1098/rspb.2018.2544 30963937 PMC6408596

[11] Knudby A, Brenning A, LeDrew E. New approaches to modelling fish–habitat relationships. Ecol Modell. 2010;221(3):503–11.

[12] Mellin C, Andréfouët S, Kulbicki M, Dalleau M, Vigliola L. Remote sensing and fish–habitat relationships in coral reef ecosystems: Review and pathways for multi-scale hierarchical research. Mar Pollut Bull. 2009;58(1):11–9. 10.1016/j.marpolbul.2008.10.010 19058817

[13] Jones GP, Syms C. Disturbance, habitat structure and the ecology of fishes on coral reefs. Aust J Ecol. 1998;23(3):287–97.

[14] Alvarez-Filip L, Carricart-Ganivet JP, Horta-Puga G, Iglesias-Prieto R. Shifts in coral-assemblage composition do not ensure persistence of reef functionality. Sci Rep. 2013;3:3486 10.1038/srep03486 24336631 PMC3860008

[15] Graham N, Nash KL. The importance of structural complexity in coral reef ecosystems. 2013;315–26.

[16] Bruno JF, Sweatman H, Precht WF, Selig ER, Schutte VGW. Assessing evidence of phase shifts from coral to macroalgal dominance on coral reefs. Ecology. 2009;90(6):1478–84. 10.1890/08-1781.1 19569362

[17] Bruno JF, Côté IM, Toth LT. Climate change, coral loss, and the curious case of the parrotfish paradigm: Why don’t marine protected areas improve reef resilience? Ann Rev Mar Sci. 2019;11:307–34. 10.1146/annurev-marine-010318-095300 30606097

[18] Ceccarelli D, Evans R, Logan M, Mantel P, Puotinen M, Petus C, et al Long-term dynamics and drivers of coral and macroalgal cover on inshore reefs of the Great Barrier Reef Marine Park. Ecol Appl. 2019;(October). 10.1002/eap.2008 31550393

[19] Fabricius K, De’Ath G. Environmental factors associated with the spatial distribution of crustose coralline algae on the Great Barrier Reef. Coral Reefs. 2001;19(4):303–9.

[20] Roberts CM, McClean CJ, Veron JEN, Hawkins JP, Allen GR, McAllister DE, et al Marine biodiversity hotspots and conservation priorities for tropical reefs. Science (80-). 2002;295(5558):1280–4. 10.1126/science.1067728 11847338

[21] Chapman MR, Kramer DL. Gradients in coral reef fish density and size across the Barbados Marine Reserve boundary: effects of reserve protection and habitat characteristics. Mar Ecol Prog Ser. 1999;181:81–96.

[22] Mcclanahan TR, Maina JM, Graham NAJ. Modeling Reef Fish Biomass, Recovery Potential, and Management Priorities in the Western Indian Ocean. 2016;1–21. Available from: 10.1371/journal.pone.0154585 PMC4858301 27149673

[23] McClanahan TR, Graham NAJ, MacNeil MA, Cinner JE. Biomass‐based targets and the management of multispecies coral reef fisheries. Conserv Biol. 2015;29(2):409–17. 10.1111/cobi.12430 25494592

[24] Mcclanahan TR. Community biomass and life history benchmarks for coral reef fisheries. Fish. 2018;19:471–88.

[25] Mcclanahan TR, Schroeder RE, Friedlander AM, Vigliola L, Wantiez L, Caselle JE, et al Global baselines and benchmarks for fish biomass: comparing remote reefs and fisheries closures. Mar Ecol Prog Ser. 2019;612:167–92.

[26] Darling ES, Mcclanahan TR, Maina J, Gurney GG, Graham NAJ, Januchowski-hartley F, et al Socio-environmental drivers inform strategic management of coral reefs in the Anthropocene. Nat Ecol Evol. 2019; 10.1038/s41559-019-0953-8 31406279

[27] Cinner JE, Maire E, Huchery C, Macneil MA, Graham NAJ, Mora C, et al Gravity of human impacts mediates coral reef conservation gains. 2018;115(27). 10.1073/pnas.1708001115 PMC6142230 29915066

[28] Wedding LM, Lecky J, Gove JM, Walecka HR, Donovan MK, Williams GJ, et al Advancing the integration of spatial data to map human and natural drivers on coral reefs. PLoS One. 2018;13(3):1–29. 10.1371/journal.pone.0189792 29494613 PMC5832214

[29] Visconti P, Di Marco M, Álvarez‐Romero JG, Januchowski‐Hartley SR, Pressey RL, Weeks R, et al Effects of errors and gaps in spatial data sets on assessment of conservation progress. Conserv Biol. 2013;27(5):1000–10. 10.1111/cobi.12095 23869663

[30] Gillett R. A review of special management areas in Tonga. Vol. FAO Fisher. 2017.

[31] Aholahi H, Aleamotu’a P, Butler D, Etika H, Faka’osi T, Hamani S, et al Status of Fanga’uta Lagoon in 2016 Report for United nations development programme (UNDP). Dep Environ MEIDECC, Nukualofa, Tonga 2017;

[32] Anon. Review of Tonga’s National Biodiversity Strategy and action plan-fifth report. Convention on Biological Diversity. 2014.

[33] Programme M of EEC change DMMI and C and S of the PRE Rapid biodiversity assessment of the Vava’u Archipelago, Kingdom of Tonga. SPREP Apia, Samoa 358pp. 2015.

[34] Purkis S, Dempsey A, Carlton R, Samaniego B, Lubarsky K, Renaud PG. Global Reef Expedition: Kingdom of Tonga Final Report. Khaled Bin Sultan Living Oceans Foundation, Annapolis, MD Vol 8. ISBN: 978-0-9975451-2-8. 2020.

[35] Adjeroud M, Briand MJ, Kayal M, Dumas P. Coral assemblages in Tonga: Spatial patterns, replenishment capacities, and implications for conservation strategies. Environ Monit Assess. 2013; 10.1007/s10661-012-2982-5 23203818

[36] Bruckner A. Global Reef Expeditions: Kingdom of Tonga Field report. Khaled bin Sultan Living oceans Foundation, Landover, MD 2014.

[37] Buckley R, Gomez-Buckley M, Stobart B, Stone K. Vava’u coral reef surveys 2017. 2017.

[38] Ceccarelli D. Vava’u SMA Project Baseline Survey Technical Report. 2016.

[39] Friedman K, Pinca S, Kronen M, Boblin P, Chapman L, Magron F, et al Tonga country report: profiles and results from survey work at Ha’atafu, Manuka, Koulo and Lofanga (November and December 2001; March to June 2002; April to June, September and October 2008). 2008.

[40] Holthus P. Coral Reef Survey Vava’u, Kingdom of Tonga. South Pacific Reg Envrionmental Progr. 1996;

[41] Kronen M. Fishing for fortunes? A socio-economic assessment of Tonga’s artisanal fisheries. Fish Res. 2004;70(1):121–34.

[42] Lovell E, Palaki A. National coral reef status report Tonga. Int Ocean Inst—South Pacific, Kiribati Fish Div ICRI), SPREP http//www.sprep.org/att/IRC/eCOPIES/Countries/Tonga/5.pdf. 2000;26.

[43] Malimali S. Socioeconomic and Ecological Implications of Special Management Areas (SMAs) Regime in the Kingdom of Tonga. 2013.

[44] Mayfield AB, Chen CS, Dempsey AC. Biomarker profiling in reef corals of Tonga’s Ha’apai and Vava’u archipelagos. PLoS One. 2017;12(11):1–28. 10.1371/journal.pone.0185857 29091723 PMC5665425

[45] Pakoa K, Friedman K, Damlamian H, Tardy E. The status of the trochus (Trochus niloticus) resource in Tongatapu lagoon and recommendations for management. 2008.

[46] Richardson L. The effect of community managed marine protected areas on the resilience of coral reefs in the Kingdom of Tonga: physical and biological back reef assemblages. Thesis Bangor Univ. 2010;

[47] Smallhorn-West PF, Bridge TCL, Malimali S, Pressey RL, Jones GP. Predicting impact to assess the efficacy of community-based marine reserve design. Conserv Lett. 2019;(August):1–8.

[48] Smallhorn-West PF, Garvin JB, Slayback DA, DeCarlo TM, Gordon SE, Fitzgerald SH, et al Coral reef annihilation, persistence and recovery at Earth’s youngest volcanic island. Coral Reefs. 2019;1–8.

[49] Smallhorn-West P, Sheehan J, Stone K, Ceccarelli D, Malimali S, Halafihi H, et al Co-management yields positive impacts for coastal fisheries resources and biodiversity conservation. Conserv Lett.

[50] Smallhorn-West P. Sheehan J, Rodriguez-Troncoso A. Malimali S, Halafihi T, Mailau S, et al Kingdom of Tonga Special Management Area report 2020. 2020. 10.1038/s41559-019-0953-8 31406279

[51] Stone K, Menergink K, Estep A, Halafihi T, Malimali S, Matoto L. Vava’u Ocean Initiative Marine Expedition Interim Report. 2017.

[52] Vieux C, Aubanel A, Axford J, Chancerelle Y, Fisk D, Holland P, et al A century of change in coral reef status in southeast and central Pacific. 2005.

[53] Vieux C. Coral reef surveys in Vava’u, Kingdom of Tonga, September 26th-October 7th, 2005. 2005.

[54] Kulbicki M, Guillemot N, Amand M. A general approach to length-weight relationships for New Caledonian lagoon fishes. Cybium. 2005;29(3):235–52.

[55] Gurney G, Darling ES. A Global Social-Ecological Systems Monitoring Framework for Coastal Fisheries Management. 2017 63 p.

[56] Beijbom O, Edmunds PJ, Roelfsema C, Smith J, Kline DI, Neal BP, et al Towards automated annotation of benthic survey images: Variability of human experts and operational modes of automation. PLoS One [Internet]. 2015;10(7):1–22. Available from: 10.1371/journal.pone.0130312 26154157 PMC4496057

[57] Beijbom O. Automated Annotation of Coral Reef Survey Images. 2015.

[58] González-Rivero M, Beijbom O, Rodriguez-Ramirez A, Holtrop T, González-Marrero Y, Ganase A, et al Scaling up ecological measurements of coral reefs using semi-automated field image collection and analysis. Remote Sens. 2016;8(1).

[59] Elith J, Leathwick JR, Hastie T. A working guide to boosted regression trees. J Anim Ecol. 2008;77(4):802–13. 10.1111/j.1365-2656.2008.01390.x 18397250

[60] Elith J, Leathwick JR. Boosted Regression Trees for ecological modeling [Internet]. 2017 Available from: 10.1111/brv.12359 28766908

[61] Team RC. A language and environment for statistical computing. R Foundation for Statistical Computing 2017 Vienna, Austria https//wwwR-projectorg/Publ. 2016;

[62] Hastie T, Tibshirani R, Friedman J. The elements of statistical learning: data mining, inference, and prediction. Springer Science & Business Media; 2009.

[63] Ceccarelli DM, Logan M, Purcell SW. Analysis of optimal habitat for captive release of the sea cucumber Holothuria scabra. Mar Ecol Prog Ser. 2018;588:85–100.

[64] Pittman SJ, Brown KA. Multi-Scale Approach for Predicting Fish Species Distributions across Coral Reef Seascapes. 2011;6(5). 10.1371/journal.pone.0020583 PMC3102744 21637787

[65] Buston PM, Elith J. Determinants of reproductive success in dominant pairs of clownfish: a boosted regression tree analysis. J Anim Ecol. 2011;80(3):528–38. 10.1111/j.1365-2656.2011.01803.x 21284624

[66] Randall JE, Williams JT, Smith DG, Kulbicki M, Tham GM, Labrosse P, et al Checklist of the shore and epipelagic fishes of Tonga. 2003.

[67] Macneil MA, Graham NAJ, Cinner JE, Wilson SK, Williams ID, Maina J, et al Recovery potential of the world ‘ s coral reef fishes. Nature. 2015;520. 10.1038/nature14358 25855298

[68] Harborne AR, Mumby PJ, Ferrari R. The effectiveness of different meso-scale rugosity metrics for predicting intra-habitat variation in coral-reef fish assemblages. Environ Biol Fishes. 2012;94(2):431–42.

[69] Hughes TP, Kerry JT, Baird AH, Connolly SR, Chase TJ, Dietzel A, et al Global warming impairs stock–recruitment dynamics of corals. Nature [Internet]. 2019; Available from: http://www.nature.com/articles/s41586-019-1081-y 10.1038/s41586-019-1081-y 30944475

[70] Harborne AR. The Nature Conservancy’s mapping ocean wealth project, and the current and potential standing stocks of coral reef fishes in five jurisdictions of Micronesia. The Nature Conservancy; 2016.

[71] Dustan P, Doherty O, Pardede S. Digital reef rugosity estimates coral reef habitat complexity. PLoS One. 2013;8(2):e57386 10.1371/journal.pone.0057386 23437380 PMC3578865

[72] Dulvy NK, Polunin NV, Mill AC, Graham NA. Size structural change in lightly exploited coral reef fish communities: evidence for weak indirect effects. Can J Fish Aquat Sci. 2004;61(3):466–75.

[73] Bellwood DR, Hoey AS, Hughes TP. Human activity selectively impacts the ecosystem roles of parrotfishes on coral reefs. Proc R Soc B Biol Sci. 2012;279(1733):1621–9. 10.1098/rspb.2011.1906 22090383 PMC3282342

[74] Tonga SD of. Tonga national population and housing census. Nuku’alofa, Tonga Tongan Bureau of Statistics 2016.

[75] Smallhorn‐West PF, Sheehan J, Malimali S, Halafihi T, Bridge TCL, Pressey RL, et al Incentivizing co‐management for impact: mechanisms driving the successful national expansion of Tonga’s Special Management Area program. Conserv Lett. 2020;e12742.

