# Supplementary material for: Biophysical and anthropogenic influences on the status of Tonga’s coral reefs and reef fish fishery
Source: PLoS One. 2020 Nov 17;15(11):e0241146. doi: 10.1371/journal.pone.0241146 (PMC7671563; doi:10.1371/journal.pone.0241146)
Supplement: S1 File — (DOCX) [file pone.0241146.s001.docx]

# Biophysical and anthropogenic influences on the status of Tonga’s coral reefs and reef fish fishery

Patrick Smallhorn-West, Sophie Gordon, Karen Stone, Daniela Ceccarelli, Siola’a Malimali, Tu’ikolongahau Halafihi , Mathew Wyatt, Tom Bridge, Robert Pressey, Geoffrey Jones

**Benthic Composition**

Benthic community composition was estimated using image analysis of ten 1 x 1 m benthic photoquadrats per transect with 15 points randomly overlaid across each image (total 150 points per transect). Given the large number of images and points required for annotation (images = 11020; points = 165300), we used the machine learning software BenthoBox (www.benthobox.com) to assist with the benthic annotations. BenthoBox automatically classifies points into benthic substrate categories from images based on training provided by a human annotator.

A label set of 22 benthic categories from four functional groups was established based on their functional relevance to coral reef ecosystems and their ability to be reliably identified from images by human and automated annotators (Beijbom et al., 2015a; González-Rivero et al., 2016). Four broad functional groups represent the main benthic components of coral reefs in Tonga: “Algae”, “Hard Coral”, “Other Invertebrates” and “Other”. The main algal groups were categorized based on their functional relevance: Crustose Coralline Algae (CCA), Macroalgae and Turf algae. Turf algae are considered a grazed assemblage of algal species up to 1 cm in height (González-Rivero et al., 2016). Hard corals comprise 12 groups classified based on a combination of taxonomy (*i.e.* family) and functional morphology. Fire corals of the family Milleporidae (Class Hydrozoa) were included in the hard coral category because they fulfil a similar functional role i.e. the provision of three-dimensional habitat complexity. Soft corals were divided into Alcyoniidae soft corals (class “Soft Corals”) and Gorgoniidae soft corals (class “Other Soft Corals”).

The aim of the automated annotation method is to learn from human annotations and automatically analyse the remaining images to within an acceptable margin of error (Beijbom, 2015a,b). While automation typically captures similar trends but with higher variability than among human annotators (Beijbom et al. 2015; González-Rivero et al., 2016), the impact of this error on interpretation depends on the relative abundance of organisms, taxonomic resolution and ecological relevance of the variables in question. Typically, the noise around automated annotations may lead to misinterpretations of rare categories (<5 % total cover) for which the average abundance is similar to the error in quantification. However, the impact of automated analysis error on more dominant benthic groups (>5 % total cover) is less pronounced, and usually has marginal effects on derived cover estimates (González-Rivero et al., 2016). For the purposes of this study we therefore included four common benthic categories each with mean cover greater than five percent: Hard Coral, Soft Coral, CCA and Turf Algae.


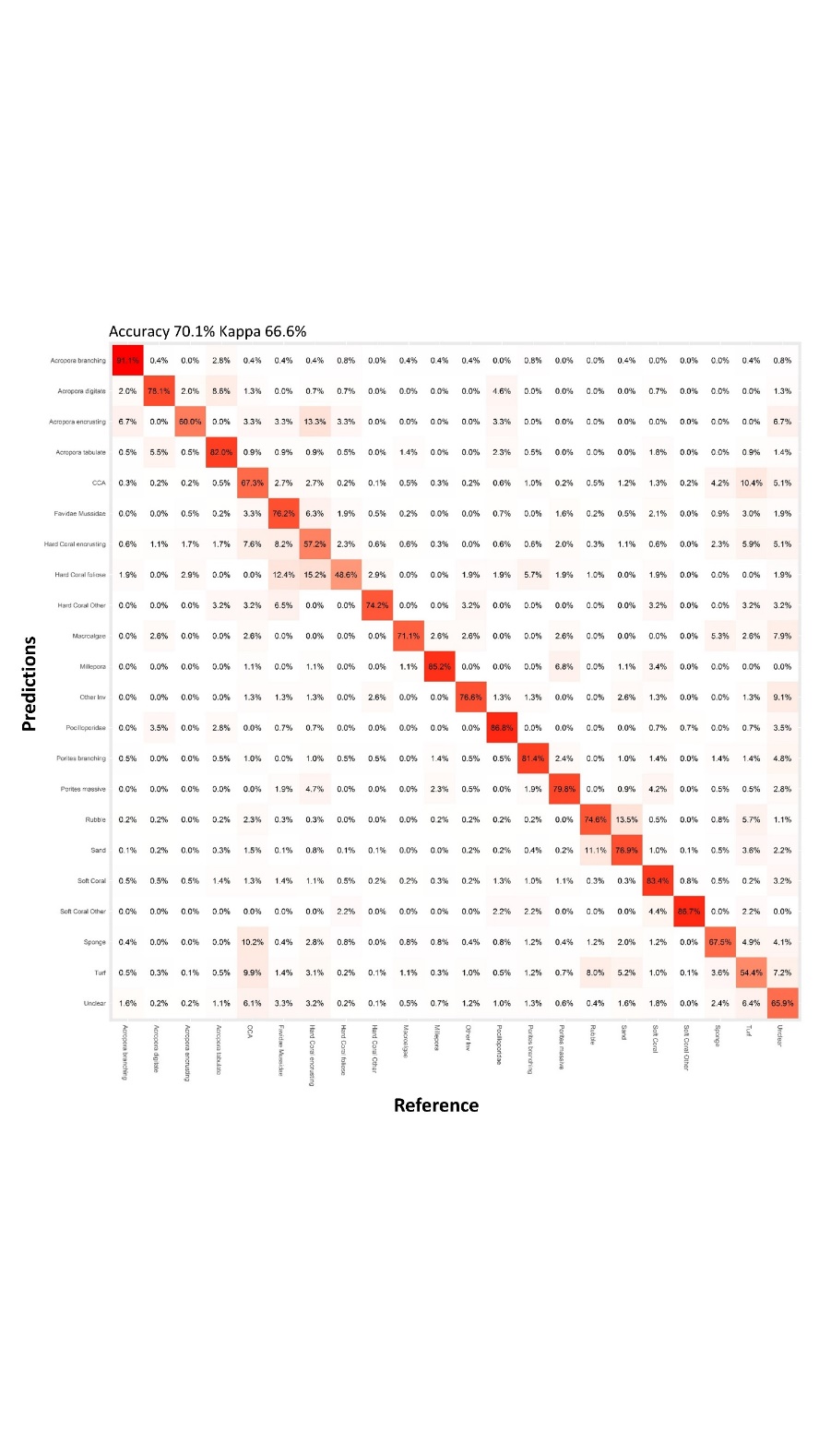
A total of 4880 images were drawn from the overall pool of images and manually annotated to use as training and validation sets for the automated annotator. This consisted of all 3880 images annotated from the Vava’u island group, and an additional 1000 images (10% of total) randomly selected from the Ha’apai and Tongatapu island groups. The Vava’u annotations had been completed as part of a previous study (Smallhorn-West et al. 2019) but were included as extra training data for the model. The validation set consisted of 10,000 random manual annotations withheld from the training dataset and instead used to compare machine predictions with human annotations. The overall error of each benthic category in percent cover was then calculated and used to determine whether the machine’s accuracy fell within acceptable bounds. Variability in the error of percent cover was calculated by randomly subsetting the holdout set into ten subcategories to generate a mean and 95% confidence intervals (Fig S1-S4)

**Fig S1.** Confusion matrix of individual benthic categories used for automated image annotation.


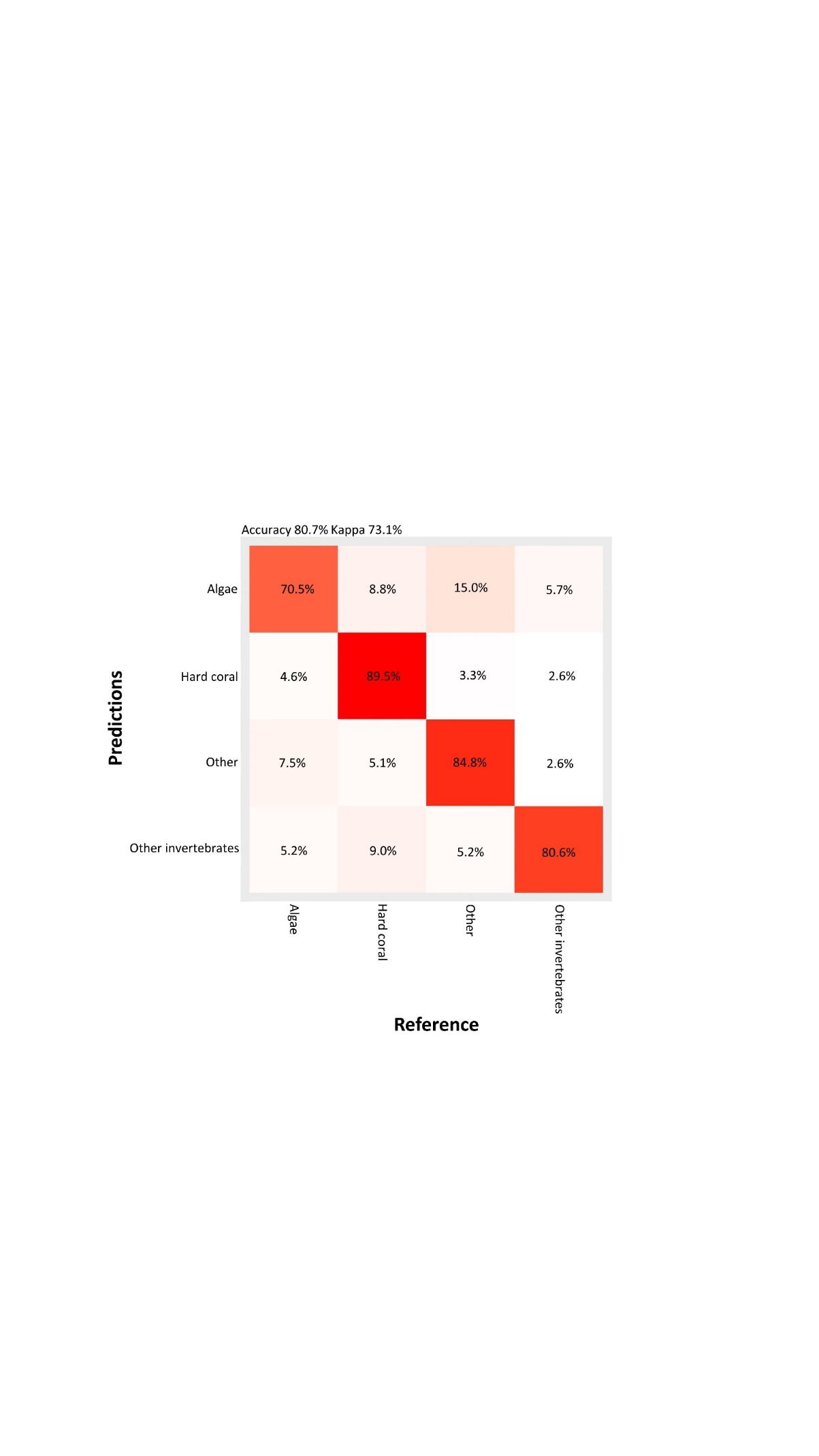


**Fig S2.** Funcitonal level confusion matrix of benthic categories used for automated image annotation.


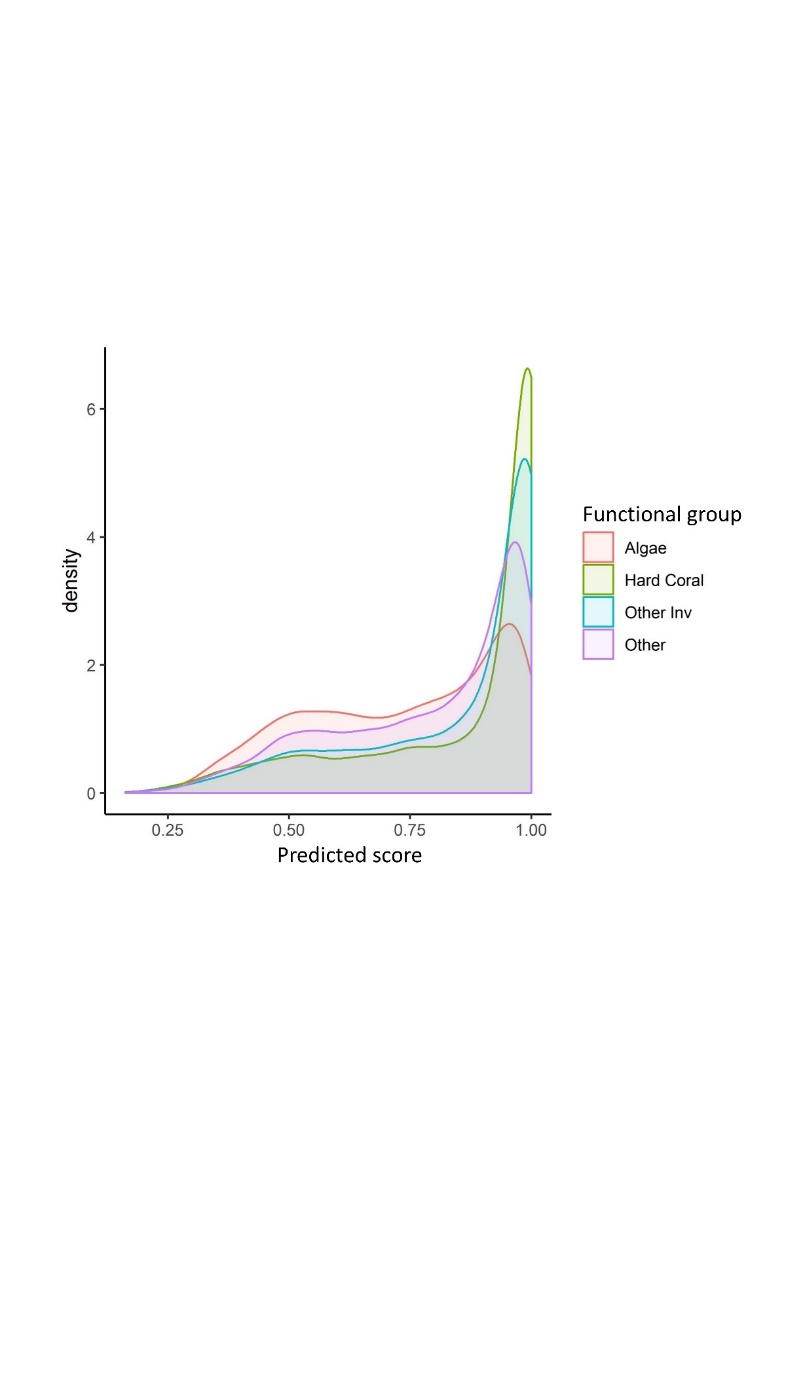


**Fig S3.** Density plot of machine confidence in functional level annotations. X-axis represents the confidence (%) that a given annotation is correct.


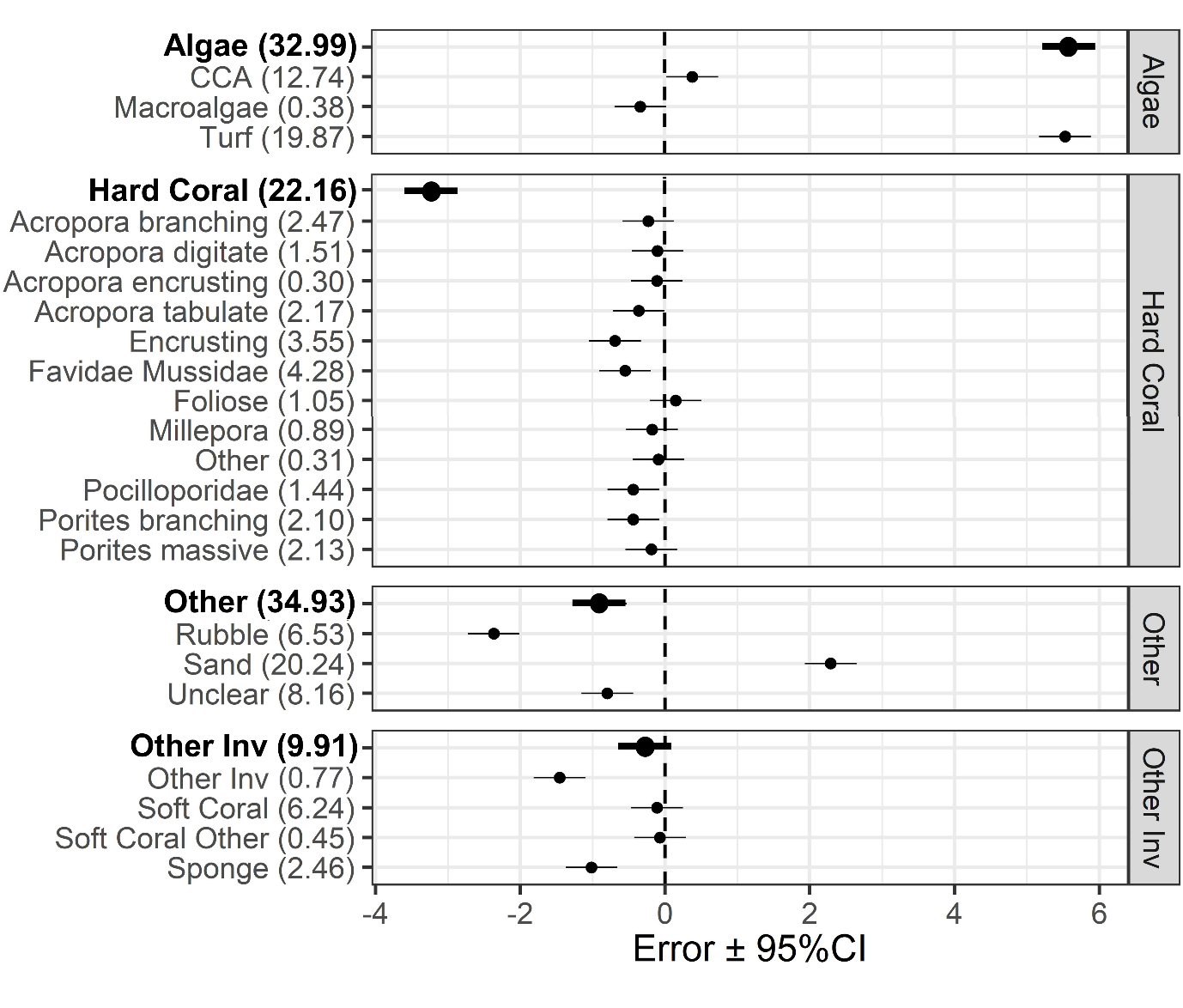


**Fig S4.** Error from automated benthic estimations of benthic cover in Tonga. Errors are presented for each one of the 22 variables and aggregated by functional groups (“Algae”, “Hard Coral”, “Other” and “Other Inv”). Points represent the mean machine error and error bars indicate the 95% confidence limits. Overall machine accuracy and kappa were 70.0% and 66.6% at the specific category level, respectively, and 80.7% and 73.1% at the functional group level, respectively. Value in parenthesis denote overall percent cover of each category.

**Socio-environmental variables**

**Coral reef density**

Coral reef density was calculated as the total area (km^2^) of coral reef habitat within a radius from each 10 m^2^ pixel defined by a buffer distance of 5 km (Fig. S5). This distance were selected because it represents the lower range of larval dispersal distances for most reef fish (Green *et al*. 2015). Coral reef habitat classification by Purkis et al. (2019) consisted of 36 habitat classes at a resolution of 2 m^2^ but was not available for the island groups of Tongatapu or Nomuka (within Ha’apai). For these island groups habitat classification by Andrefouet *et al.* (2006) was used (24 classes, 30 m^2^ resolution). While determining the most accurate degree of connectivity between reefs in Tonga will depend on both biophysical modeling of dispersal patterns and genetic parentage analysis, it was beyond the scope of this study to complete a comprehensive assessment of connectivity at this level within Tonga’s >15,000 km^2^ of reef habitat (Bode *et al.* 2019). These reef density layers therefore represent a first approximation of potential patterns of connectivity.

Reef habitat for Vava’u and Ha’apai was defined from Purkis *et al.* (2019) habitat classification and included the following habitats: shallow fore reef terrace, shallow fore reef slope, reef crest, lagoon pinnacle reefs (massive coral dominated and calcareous red algae conglomerate), lagoon floor bommies, lagoon patch reefs, lagoon fringing reefs, deep forereef slope, back reef pavement, back reef coral framework, and back reef coral bommies. Reef habitat for Tongatapu and Nomuka was defined from Andrefouet *et al.* (2006) habitat classification, and included the following habitats: subtidal reef flat, shallow terrace with constructions, reef flat, forereef on terrace, and fore reef. A raster layer with all included reef layers was generated by assigning a value of 1 to each 10 m^2^ pixel containing reef habitat, and a value of 0 for pixels containing non-reef habitat. The *focal statistic* tool was then used to calculate the sum of the number of pixels within a 5 km radius of each 10 m^2^ pixel of reef area in Tonga. The resulting value was then converted to units of km^2^.

**
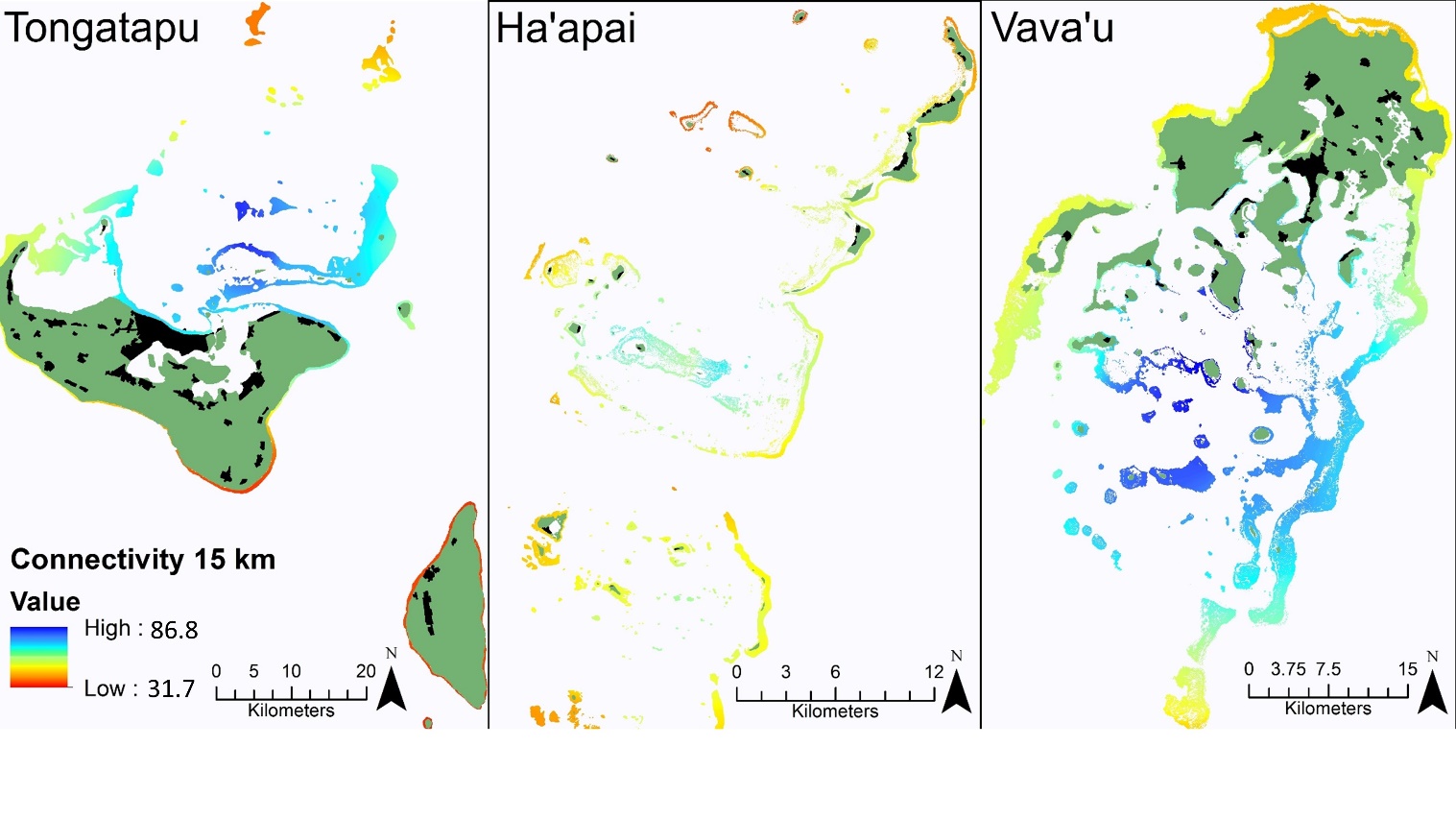

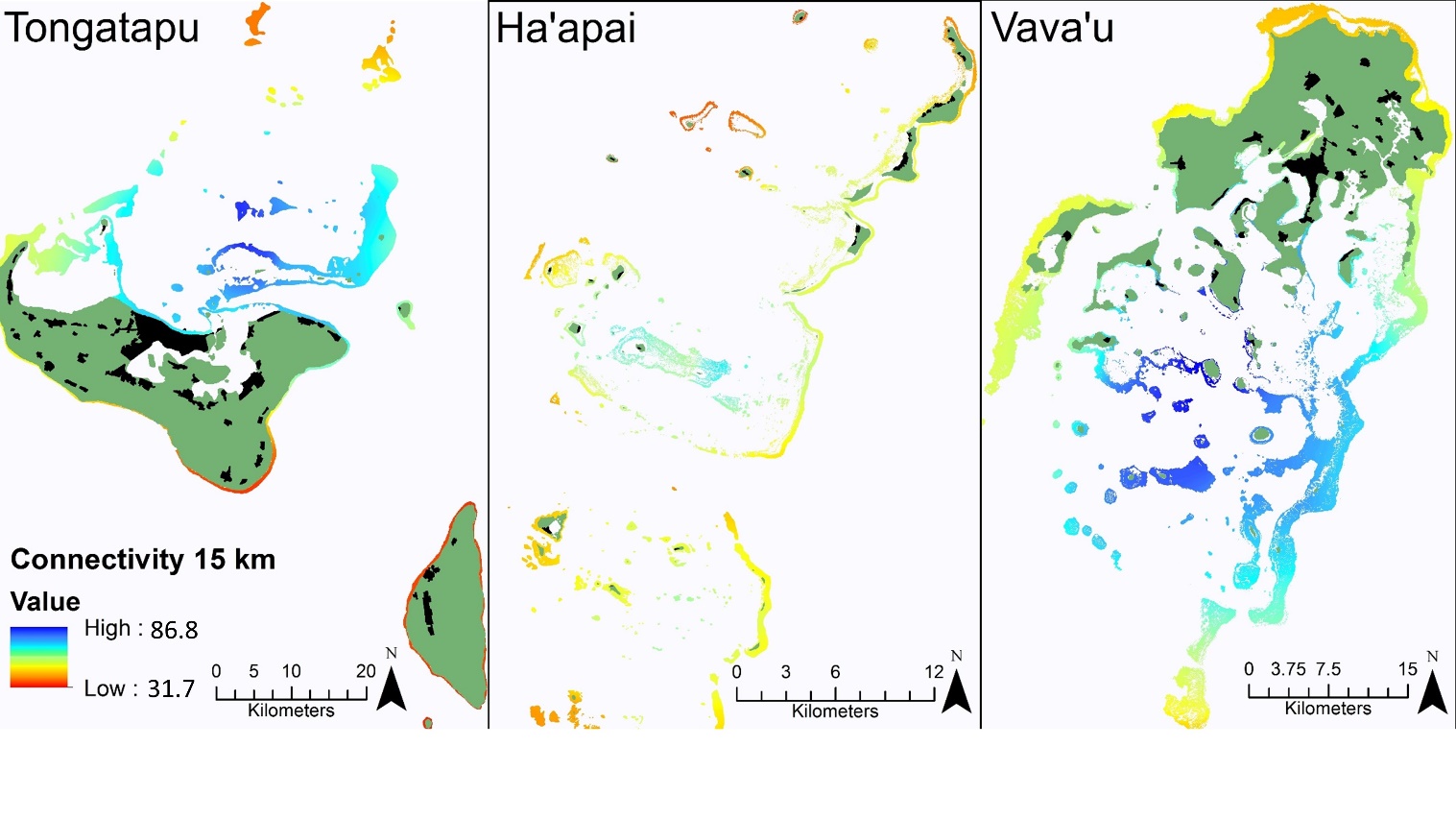
**

**Fig S5.** Coral reef density in Tonga measured as the amount of reef habitat in within a 5 km radius of each 10 m^2^ reef pixel. Green areas represent land and black areas represent villages.

**Cyclone occurrence within the past 18 months**

Tropical cyclones cause significant damage to coral reef ecosystems in the South Pacific (Fabricius et al. 2008; De’eath et al. 2012). Several studies have shown that maximum wind speed is a better predictor of reef damage than cyclone duration (Fabricius et al. 2008, De’ath et al. 2012). Severe damage to reef structure is likely at wind speeds above approximately 50 knots, or 25 m/s (Done, 1992 – 25-33 m/s; Puotinen et al. 2007 – 24 m/s; 28 m/s - Fabricius et al. 2008), which corresponds to the lower extent of a category 2 cyclone on the Australian tropical cyclone intensity scale. De’ath et al. (2012) also demonstrated that the best temporal predictor of reef damage was the occurrence of a cyclone within the past 1.5 years, or 18 months.

We downloaded all cyclone tracks crossing Tonga’s coral reefs from the NOAA IBTrACS websites (<https://www.ncdc.noaa.gov/ibtracs/index.php?name=ib-v4-access>) as point based shapefiles. All points with a wind speed below 50 knots were deleted. The R50 value, which indicates the distance from each point that also received a minimum of 50 knots, was used to create a buffer around each point. The date of the cyclone was then used to determine if any of the survey sites fell within these buffer zones in the 18 months following the cyclone. All of Tongatapu and Southern Ha’apai were hit by cyclone Gita in February 2018, six months prior to the surveys. Parts of Vava’u were also hit by cyclone Ula (January) and Winston (March) in early 2016.

**Distance from provincial capital**

Globally, distance to fish markets has a strong explanatory role in the structure of reef fish biomass (Brewer et al. 2012; Cinner et al. 2013). Likewise, anthropogenic influences also increase towards population centers. Market access can also be a better predictor of the condition of reef fish fisheries than the density of local human populations alone (Cinner and McClanahan 2006). Three main fish markets exist in Tonga, associated with the capital of each island group. The Tongatapu fish market is located at the small boats harbor near the Nuku’alofa wharf. The Vava’u fish market is situated at the main commercial wharf in Neiafu. While not permanent, in Ha’apai most reef fish are sold commercially at the Pangai wharf. The distance from the nearest of these three locations to each 10 m^2^ pixel (marine extent defined by Andrefouet *et al.* (2006)) was therefore calculated using the *Euclidean distance* function (Fig. S6).

**
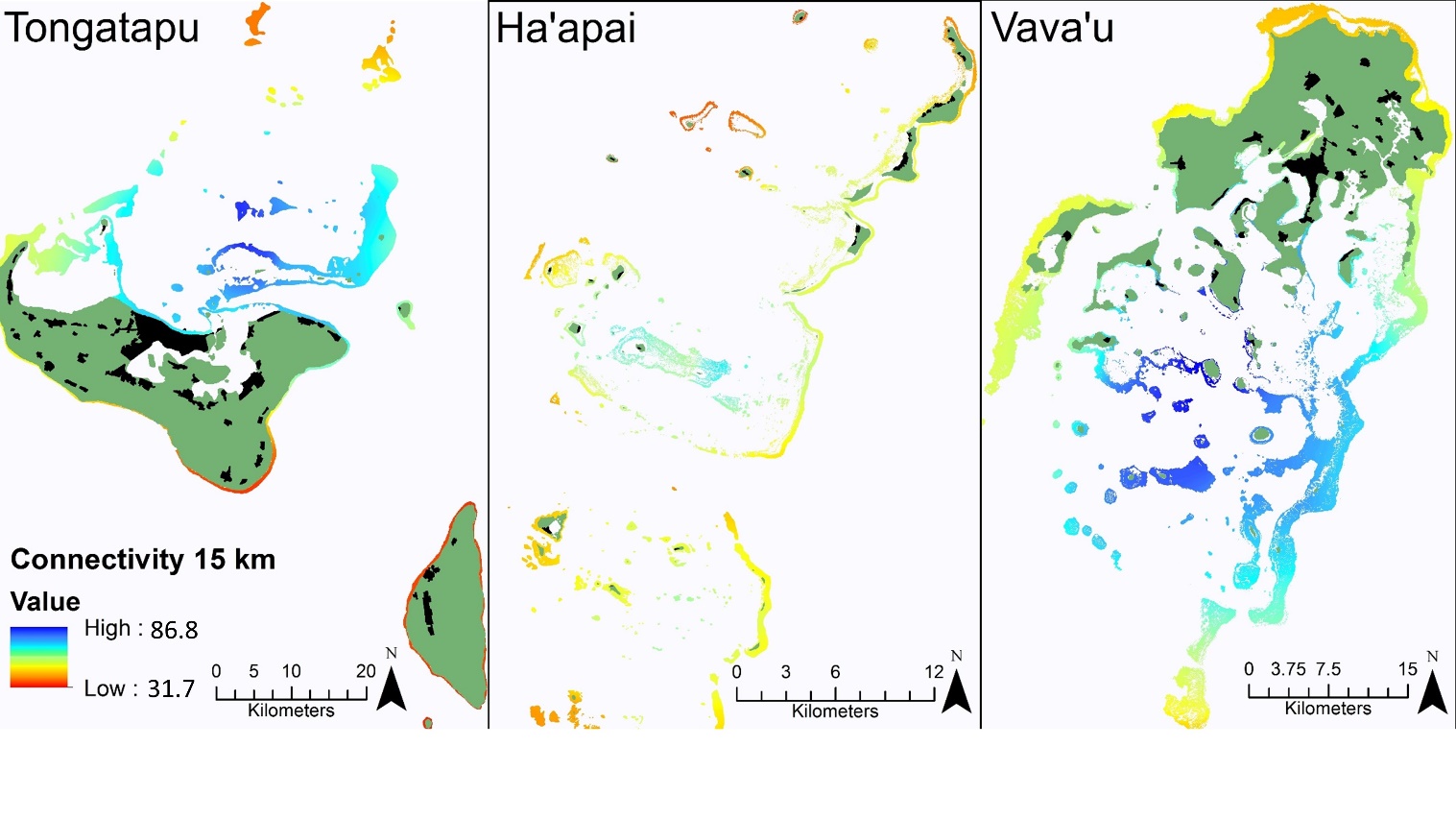
**
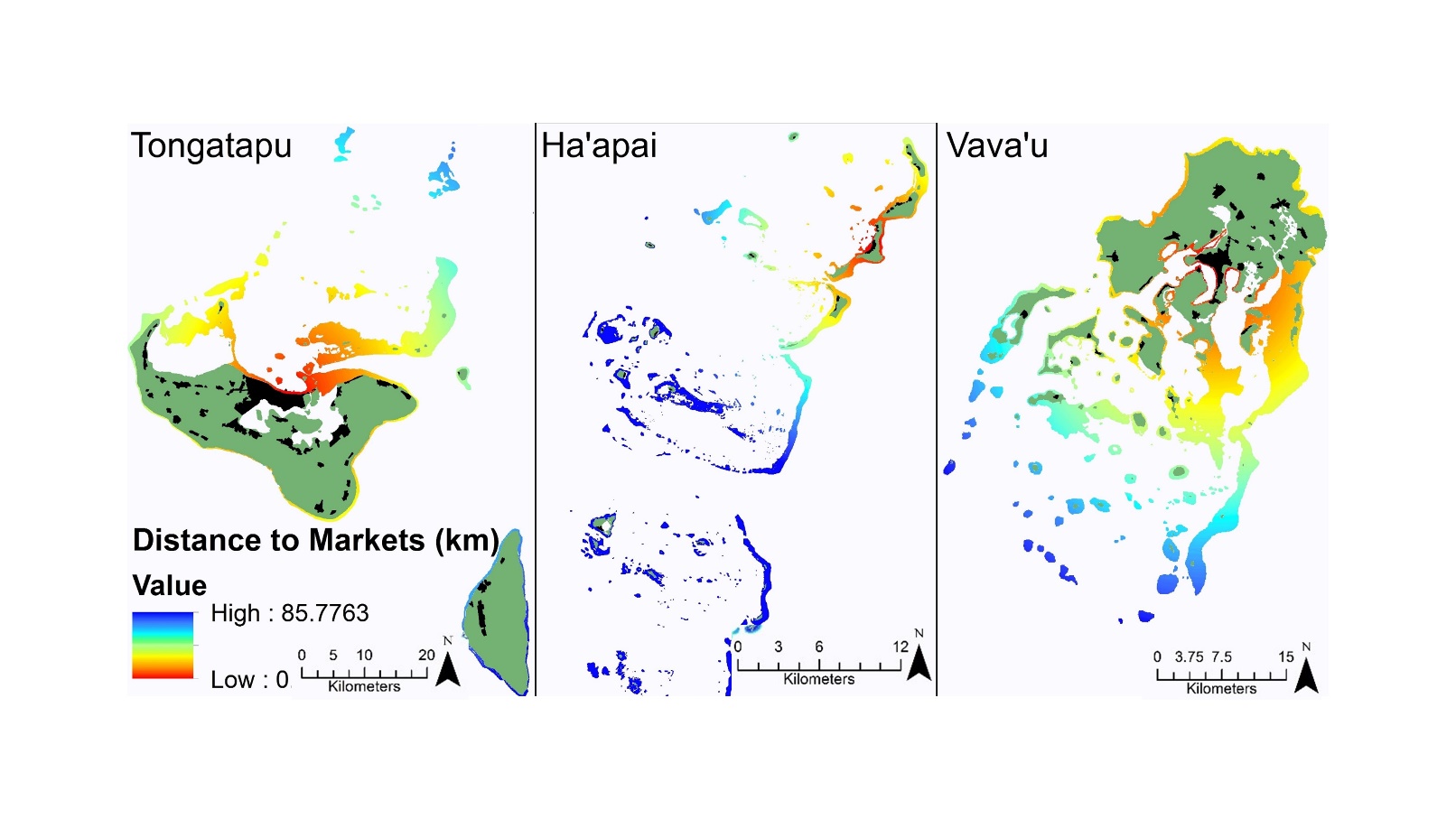


**Fig S6.** Distance to the three main fish markets for each 10 m^2^ pixel of Tonga’s near-shore marine environment. Green areas represent land and black areas represent villages.

**Fishing pressure**

Reef fish fisheries in Polynesia are critical for maintaining livelihoods and food security (Kronen 2004). Fishing pressure is a strong determinant of many metrics of reef health, and one of the most direct ways that humans interact with coral reefs (Cinner et al. 2018). Metrics of fishing pressure are often calculated using fisheries-dependent data (e.g. catch data). However, while some catch data are available from Tonga, they lack the spatial and temporal resolution, wide spread coverage and detail required to build an accurate model of fishing pressure for the entire region. Furthermore, current fishing activities may not be an accurate reflection of long-term trends, as fishers will likely change fishing grounds as stocks become depleted (Ochiewo 2004).The current study therefore used a combination of census data and key informant interviews to build a historical model of relative fishing effort across the reef fish fishing grounds of Tonga (Fig. S7, S8). This model represents a unit-less value of relative fishing effort that assumes fishers minimize travel time and only extend their range as closer stocks become depleted.

The reef fish fishery in Tonga can be broadly divided into commercial and subsistence fishing, each with different patterns of resource use and behavior (Kronen 2004). Key informant interviews were used to ascertain the specific details of both fishing practices, and took place during regular training meetings between Ministry of Fisheries staff and communities implementing new management areas. Interviews were conducted with both Ministry of Fisheries staff as well as local fishers (who classified themselves as either mostly commercial or mostly subsistence fishers). Twelve fishers from four villages agreed to participate in short informal interviews to discuss their fishing practices (Smallhorn-West et al. 2019). Fishers were asked the type of fishing they engage in, the methods employed and if willing, to outline on a map their fishing grounds.

The 2016 national census reported 2301 individuals in Tonga who identify as fishers. Of these, 1868 fish mainly for subsistence, while the remaining 433 reported fishing predominantly for commercial purposes (Statistics Department Tonga, 2016). Commercial fishing in Tonga is an organized profession, in which groups of fishers go out in boats at night time to fish an area of reef (Kronen 2004, Smallhorn-West *et al.* 2019). Following a night of fishing commercial fishers generally travel to the main fish markets and sell their catch to middlemen who run stalls in town and on roadsides. While commercial fishers also often engage in subsistence fishing, it is rare for subsistence fishers to fish commercially (Kronen 2004, Smallhorn-West *et al.* 2019). Subsistence fishing is here defined as ‘fishing mainly for personal consumption or for that of family or gifts.’ In contrast, subsistence fishing is much more opportunistic. Subsistence fishing is generally shore based and practiced close to the villages, with fishers swimming out from shore (Kronen 2004, Smallhorn-West *et al.* 2019).

Census data and key informant interviews were used to build a model of fishing pressure for Tonga, using similar methodology to Smallhorn-West *et al.* (2019). While village level population data was available from the 2016 national census, only district level data was available on fishing practices. Therefore, the village level abundance of commercial and subsistent fishers targeting reef fish was calculated by: 1) dividing the district level population of commercial and subsistence fishers by the population of each village; 2) multiplying the resulting value by the district level proportion of fishers who target reef fish, and 3) multiplying each value by a constant representing the proportional difference in total catch for each type of fishing, to account for differences in total catch between commercial and subsistent fishers.

An economic assessment of fisheries types in Tonga by Kronen (2004, Table S1) suggested that there was no clear economic distinction between commercial and subsistence coastal fisheries, however both national census data and key informant interviews suggested that fishers consistently identify themselves according to these categories. We therefore categorized Kronen (2004) Group 1 individuals as ‘subsistence’ and Group 3 as ‘commercial’ (Table 2). Group 1 individuals are predominantly shore based and align with subsistence practices. Group 3 fishers are exclusively spear fishers, fishing predominantly at night, which align with key informant interview findings of commercial practices. The proportional difference in catch between groups was calculated using total catch week^-1^ (kg) values of 40 and 75 kg respectively (Kronen 2004, Table S2). The abundance of commercial and subsistence fishers in each village was then multiplied by the proportional difference between these values, centered around 1 (1.30 commercial, 0.695 subsistence). The values for each village therefore represent the number of commercial or subsistence fishers who target reef fish, weighted by proportional differences in total catch (kg week^-1^).

**
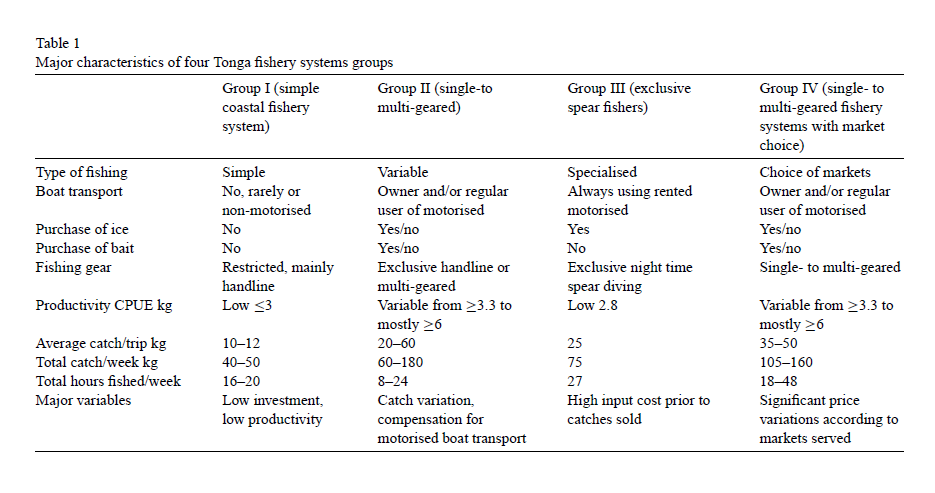
Table S1**. Major characteristics of four Tonga fishery systems groups from Kronen (2004).

To extrapolate fisher abundance across the reef fish fishing grounds of Tonga, polygons of each village (142 total) were created and converted to points. The fishing grounds for reef fish in Tonga are defined as all reef habitat from Andrefouet *et al.* (2006) and Purkis *et al.* (2019). The *heatmap* function (QGIS V.2.14) was then used to create separate decay kernels that extrapolated the weighted abundance of commercial and subsistence fishers across the reef habitat of Tonga. Key informant interviews established that commercial fishers fish every part of their island group, from inner to outer islands. The decay kernel extent was therefore set to 30 km, corresponding to the outer extent of each island group. Subsistence fishing is generally limited to the waters close by each village, and

**
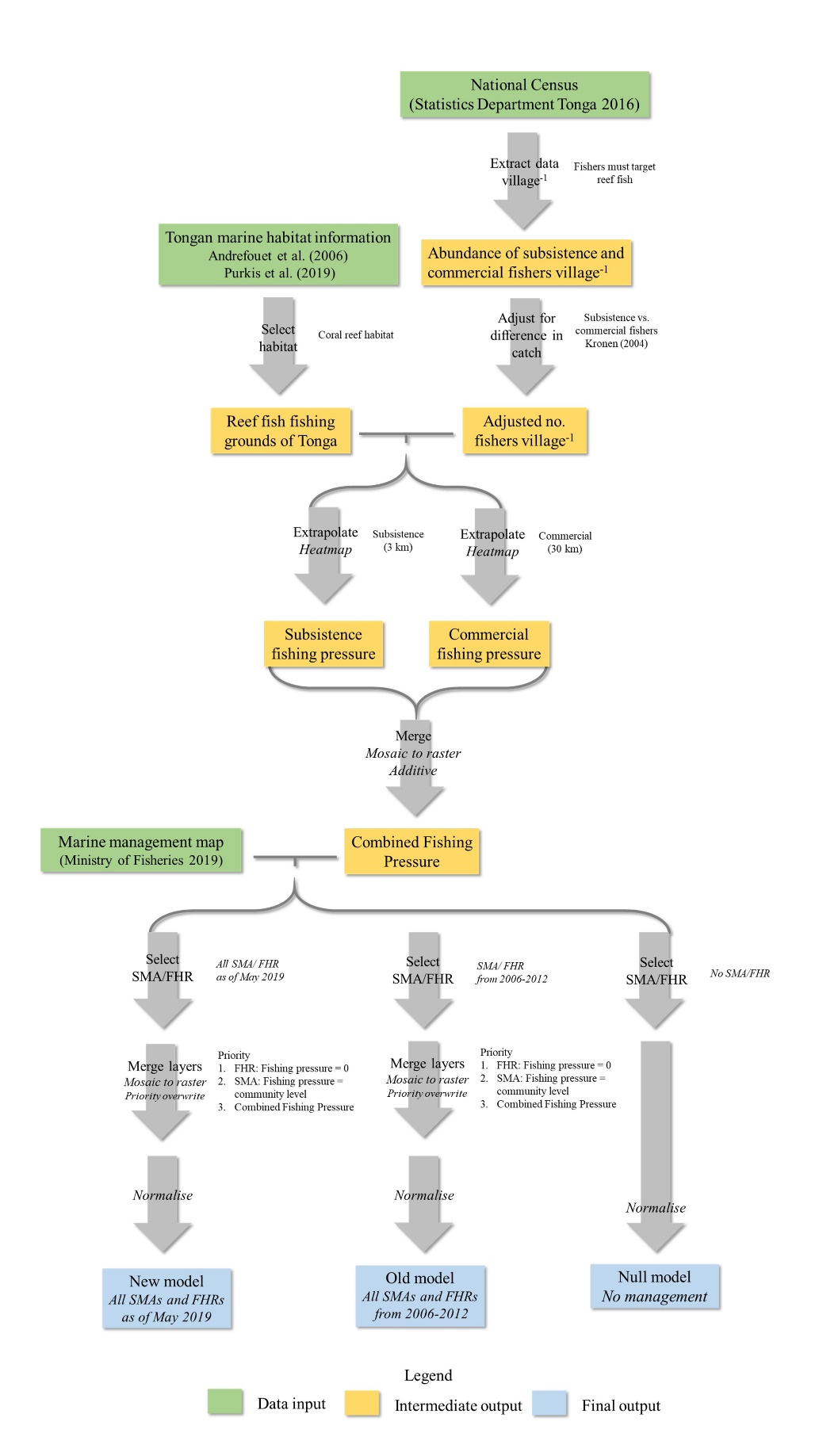
**

**Figure S7.** Flow chart representing the steps used to build three fishing pressure models for Tonga.

therefore the kernel extent was set with a cut-off of 3 km around each village. This distance is based on the maximum distance identified as fishing grounds by subsistence fishers during key informant interviews. All values of fishing pressure in Fish Habitat Reserves (FHRs) were set to 0, and Special Management Areas (SMA) values set to the sum of commercial and subsistence fishers from each corresponding SMA. This model therefore assumes full compliance by fishers. One caveat in this model is that many SMAs and FHRs have only been implemented recently and therefore values created might not represent accurate long term trends in fishing effort. The current study therefore also created two additional fishing pressure layers: 1) raw fishing pressure, values without any adjustments for management practices (Null model), and; 2) a layer only including SMAs/FHRs implemented more than five years previously (Old model).

Commercial and subsistence fishing pressure heatmaps, as well as specific fishing pressure values for each FHR and SMA were merged using the *mosaic to new* *raster* function (ArcMap V10.4.1). This function added commercial and subsistence values together, but overruled them if the area corresponded to an SMA and/or FHR. This raster layer was subsequently clipped by the coral reef habitat of Tonga using the *extract by mask* function. Lastly, these values were normalized to provide values ranging between 0 and 100.


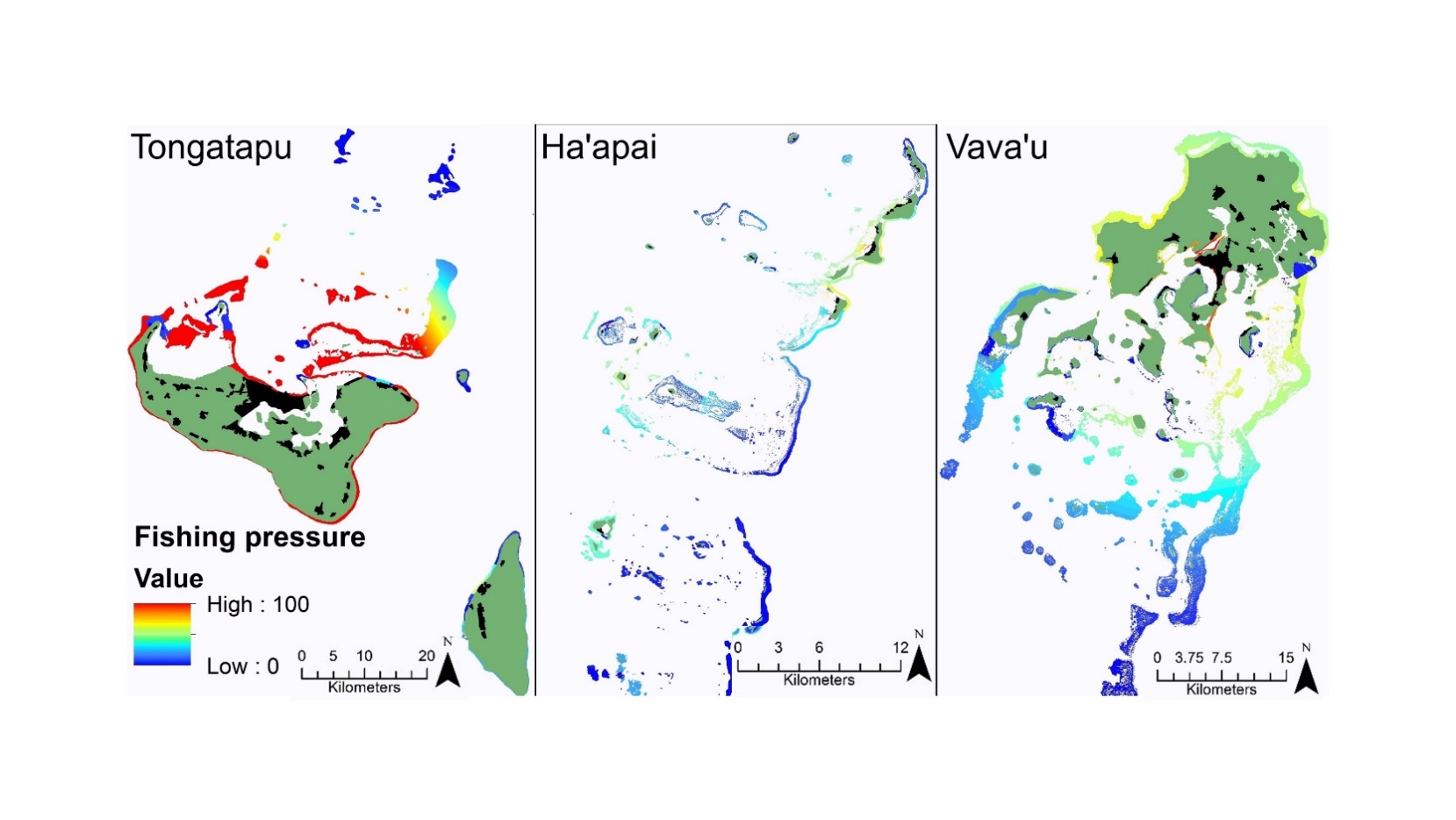
The final fishing pressure metric represents a unit-less value of relative fishing effort throughout the region. This metric assumes that, all else being equal, fishers preferentially select sites closer to home and extend their range as close locations become exhausted. While the model is therefore likely decoupled from current fishing effort, it is nonetheless useful in that it constitutes the historical impact of fishing on reef fish assemblages in Tonga.

**Figure S8.** Relative fishing pressure for Tonga’s coral reef ecosystem measured as the catch adjusted village level abundance of commercial and subsistence fishers extrapolated across the fishing grounds of Tonga. This figure represents the Current model, which includes all SMAs and FHRs as of 2019. Green areas represent land and black areas represent villages.

**Land area**

Local marine community structure and productivity may be influenced by terrestrial nutrients and runoff into the marine ecosystem (Fabricius 2005). Total land area, as well as distance from land may therefore also act as a useful metric for the degree of terrestrial influence on near-shore marine ecosystems. The total land area within a 5 km buffer zone of each 10 m^2^ pixel was calculated as an additional proxy for terrestrial influence. A five kilometre buffer were selected as previous studies found that nutrient inputs from terrestrial sources are commonly detectable in primary producers near to shore (Lapointe and Clark 1992). While Yeager et al. (2017) acknowledge that riverine plumes may affect the marine environment up to 50 km from the coast (Delvin and Brodie 2005), in most cases the effects are limited close to shore (Fabricius 2005). A raster layer was generated by assigning values of 1 for all land pixels and values of 0 for all marine pixels. The *focal statistics* tool was then used to calculate the sum of pixel values within a 5 km radius. Lastly, the *extract by mask* function was used to clip the large resulting layer by the extent of Tonga’s near-shore marine ecosystem (Fig. S9).

**
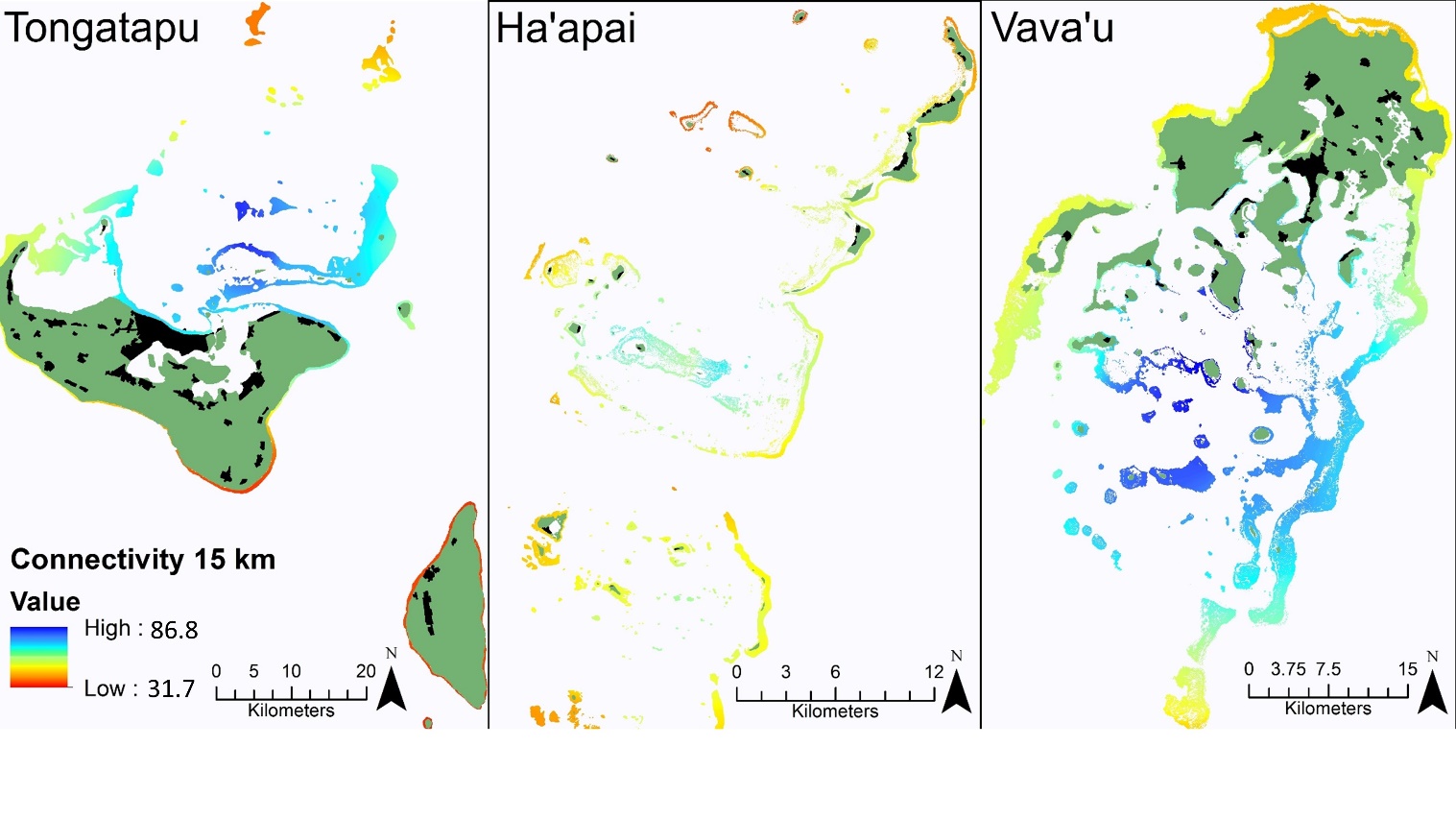
**
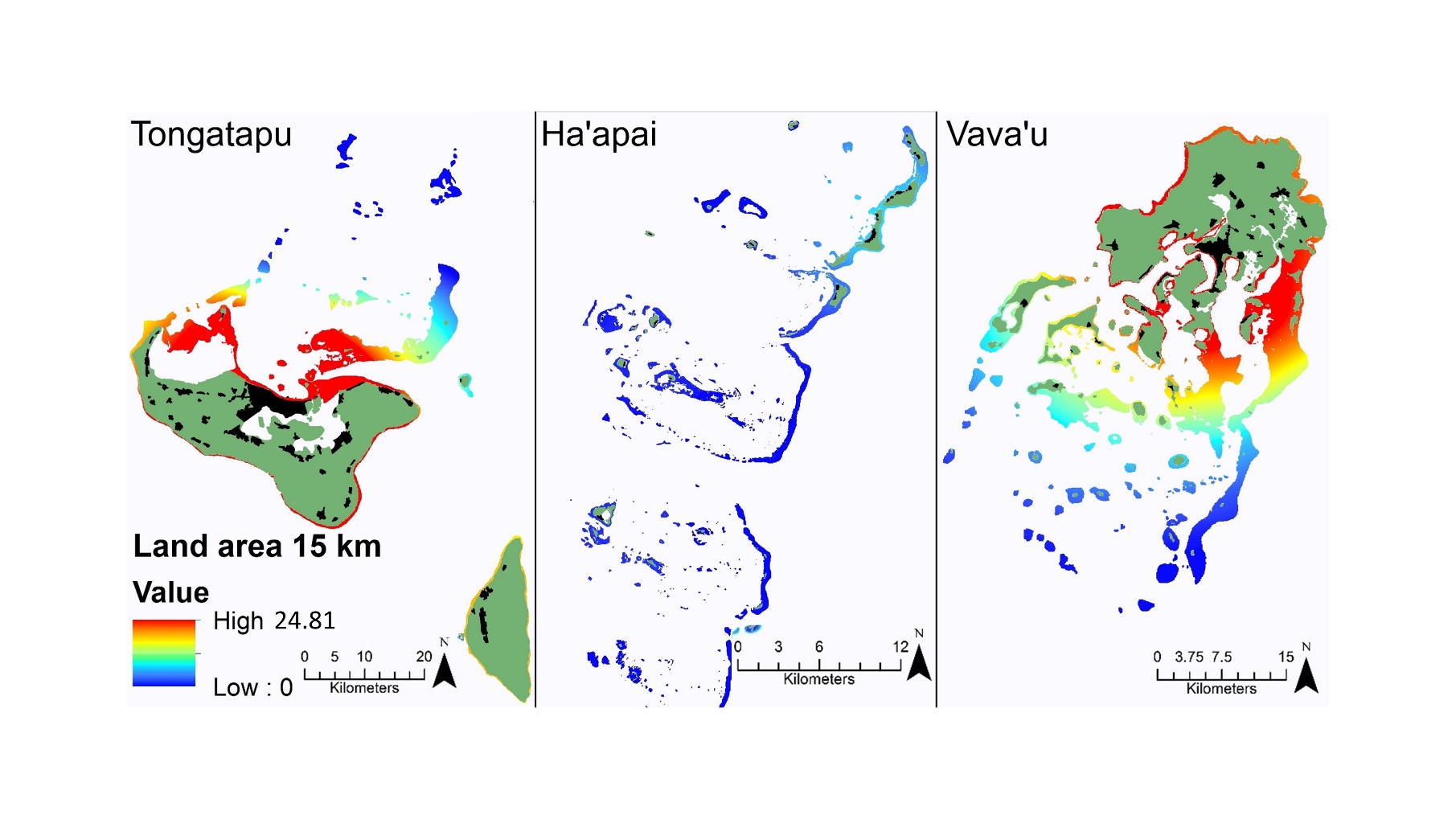


**Fig S9.** Total land area (km^2^) within 5 km of the near-shore marine ecosystem of Tonga. Green areas represent land and black areas represent villages.

**Sea surface temperature**

Temperature is a primary abiotic factor affecting the physiology of marine organisms (Brett 1971; Harborne 2016), including algal productivity (Hatcher 1990) and thus potentially the demographics of herbivorous fishes (Harborne 2016). The recurrent mass bleaching of coral reefs globally is also directly linked to sea surface temperature (SST) (Hughes et al. 2017). Coral bleaching events are primarily associated with variability in sea surface temperature, and the metric Degree Heating Weeks (DHW) is commonly used as a proxy for heat stress events. Nine SST variability layers are available from the NOAA coral reef watch website (<https://coralreefwatch.noaa.gov/product/thermal_history/index.php>), including average time between stress events and number of stress events since 1985, at DHW0, DHW4 and DHW8 respectively. However, the resolution of these layers (5 km) was too coarse for use in the current study. DHW4 and DHW8 layers were included in initial models, but were unable to explain patterns of observed coral bleaching. Given the observations of clear recent bleaching events at many sites, we suspect that patterns of bleaching within Tonga may be too fine scale for these layers to be of use. Consequently, general patterns in SST across Tonga were instead included in this dataset, with the hypothesis that corals living closer to their thermal threshold may be more likely to experienced bleached in the past. (Fig. S10).

Mean annual sea surface temperature (SST) was extracted from Sbrocco and Barber (2013) MARSPEC global ocean layers. Sbrocco and Barber (2013) obtained satellite measurements of SST at 2.5 arc-minute resolution (approximately 4 km^2^) from Aqua-MODIS 4-micron night-time SST level 3 standard mapped image products, downloaded from NASA’s Ocean color website ([*http://oceancolor.gsfc.nasa.gov/*](http://oceancolor.gsfc.nasa.gov/)*).* Monthly climatological means from September 2002 to August 2010 were used to calculate mean annual SST. Global layers were clipped by the extent of Tonga’s nearshore oceanic environment. Temperature is presented in degrees Celsius.


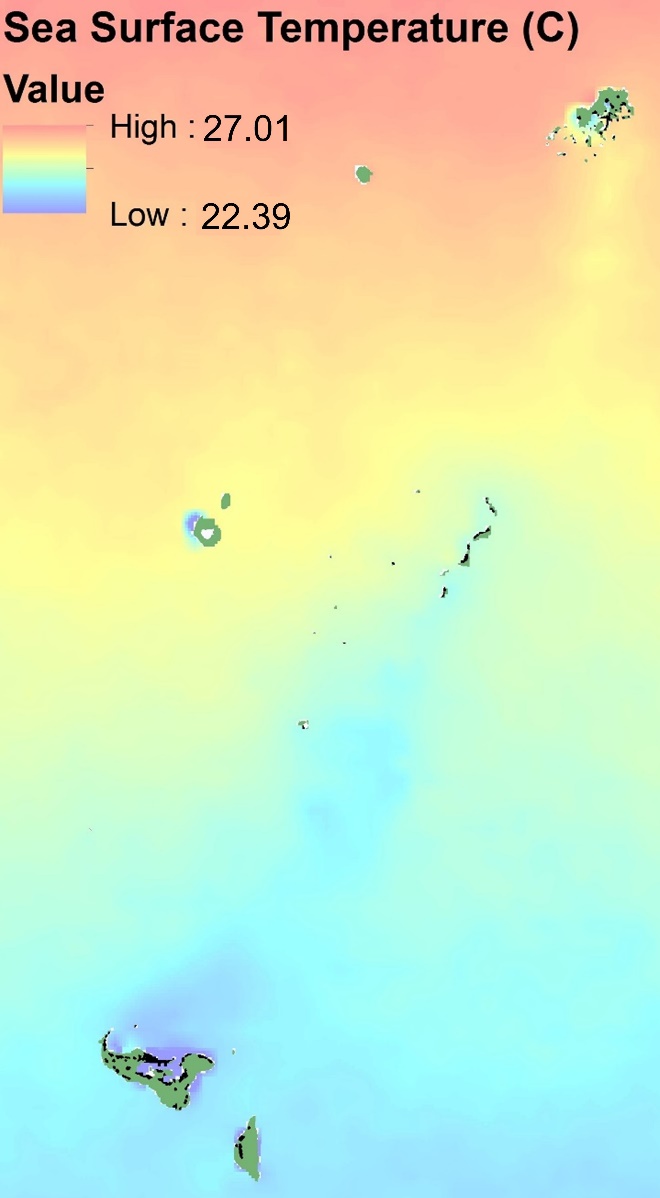


**Fig S10.** Mean annual sea surface temperature (SST) of Tonga’s marine environment in degrees Celsius.

**Wave energy**

Wave exposure is an important variable structuring coral reef communities (Fulton et al. 2005) and can have significant effects on both fish assemblages and benthic habitat types. While it is in itself a well-established natural influence on coral reef community structure, it can also limit access by fishing boats and thereby reduce fishing pressure (Chollett et al., 2014; Taylor et al., 2014). High wave exposure limits the growth and morphology of reef building corals (Chollett and Mumby, 2012), and can also increase the productivity of algae, influencing the density of herbivorous fishes (Mumby et al., 2013).

Mean wave energy, calculated as joules per square meter, was calculated using the University of Guam Marine Lab (UOGML) Wave Energy Tool (Fig. S11). A detailed description of methodology is provided in Jenness and Houk (2014) and Ekebom et al. (2003). Mean wind speed and direction were calculated from weekly wind speed and direction obtained from QuikSCAT satellite scatterometer data. Land and reef flat habitat layers from Andrefoeut *et al.* (2006) were then used to calculate fetch to the nearest landmass, reef flat or reef crest. Mean wave energy was then calculated using wind speed, direction, fetch and linear wave equations (Ekebom et al. 2003). While this data only accounts for surface wave exposure, it is likely to be a good estimate of the exposure experienced in each cell, since this project is designed for use in shallow-water, near-shore habitats. Due to extended processing times, grid cell size was set to 200 m^2^, then outputs smoothed twice using the *filter* function and *resampled* to 10 m with binary weighting to produce a 10 m^2^ resolution.


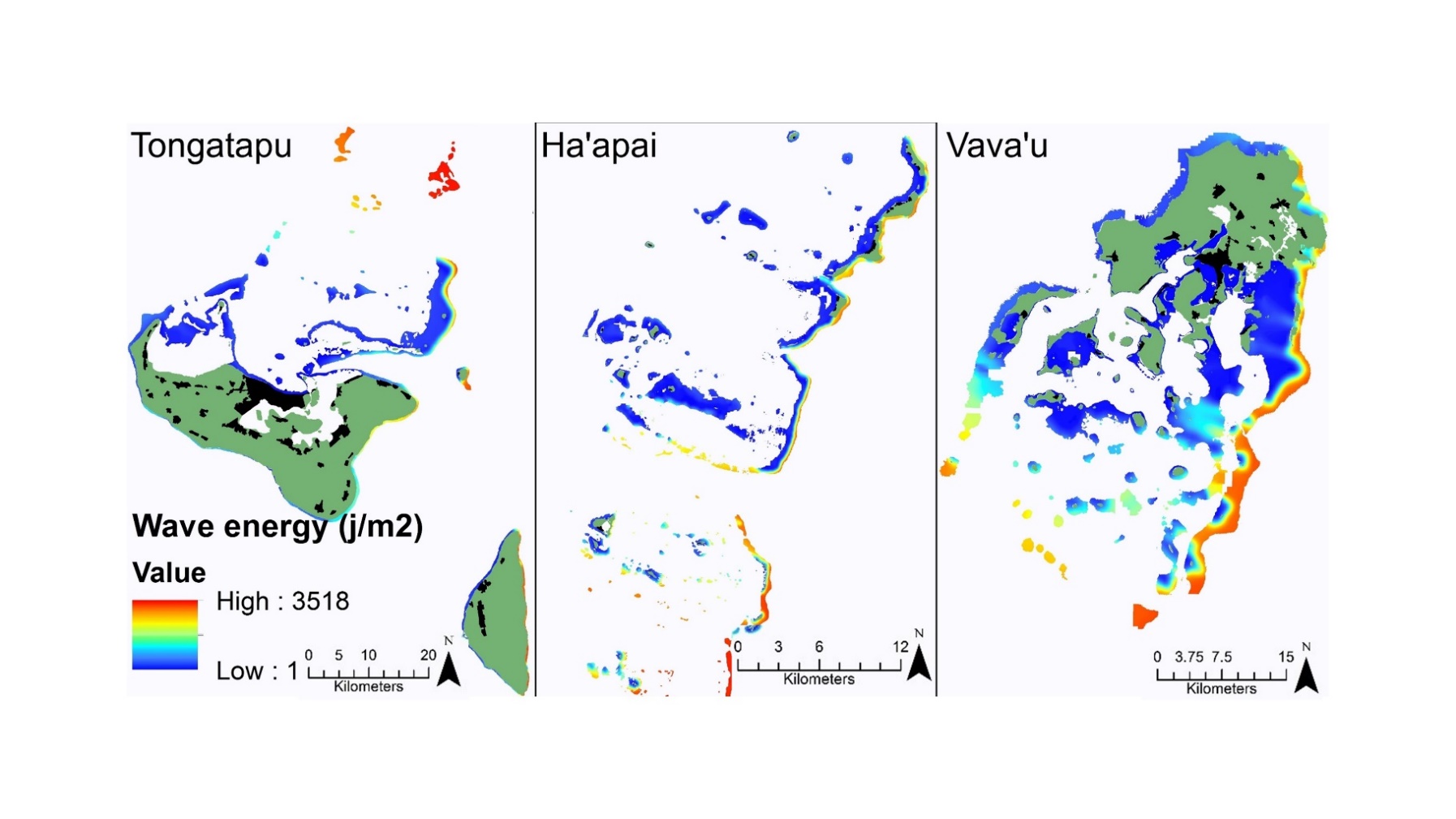


**Fig S11.** Mean wave energy, calculated as joules per m^2^, for each 10 m^2^ pixel of Tonga’s near-shore shallow marine environment. Green areas represent land and black areas represent villages.

**Table S2.** Generalized linear model outputs testing for differences in the percent cover of four key benthic categories between the main island groups of Tonga. Data transformations are listed in italics.

| **Hard coral** *Log(x+1)* |  |  |  |  |
| --- | --- | --- | --- | --- |
| Variable | Estimate | std. error | t value | p value |
| Intercept | 2.65 | 0.07 | 37.99 | <0.05 |
| Northern Ha'apai | 0.04 | 0.95 | 0.45 | 0.65 |
| Southern Ha'apai | 0.64 | 0.10 | 6.35 | <0.05 |
| Tongatapu | 0.39 | 0.09 | 4.27 | <0.05 |
| Vavau | -0.79 | 0.08 | -10.01 | <0.05 |
|  |  |  |  |  |
|  |  |  |  |  |
| **Soft Coral**  *Log(x+1)* |  |  |  |  |
| Variable | Estimate | std. error | t value | p value |
| Intercept | 2.05 | 0.07 | 29.33 | <0.05 |
| Northern Ha'apai | -0.51 | 0.09 | -5.36 | <0.05 |
| Southern Ha'apai | 0.60 | 0.10 | 5.96 | <0.05 |
| Tongatapu | -0.32 | 0.09 | -3.50 | <0.05 |
| Vavau | -1.49 | 0.08 | -18.69 | <0.05 |
|  |  |  |  |  |
|  |  |  |  |  |
| **CCA**  *Negative binomial* |  |  |  |  |
| Variable | Estimate | std. error | t value | p value |
| Intercept | 2.94 | 0.93 | 31.71 | <0.05 |
| Northern Ha'apai | -0.23 | 0.13 | -1.85 | 0.06 |
| Southern Ha'apai | 0.11 | 0.13 | 0.83 | 0.41 |
| Tongatapu | -0.12 | 0.12 | -0.97 | 0.33 |
| Vavau | -0.01 | 0.11 | -0.08 | 0.93 |
|  |  |  |  |  |
|  |  |  |  |  |
| **Turf**  *Log(x+1)* |  |  |  |  |
| Variable | Estimate | std. error | t value | p value |
| Intercept | 45.96 | 1.90 | 24.21 | <0.05 |
| Northern Ha'apai | 10.20 | 2.59 | 3.94 | <0.05 |
| Southern Ha'apai | -16.80 | 2.75 | -6.11 | <0.05 |
| Tongatapu | 1.84 | 2.52 | 0.73 | 0.47 |
| Vavau | 16.19 | 2.17 | 7.45 | <0.05 |

**Table S3.** Generalized linear model outputs testing for differences in key reef fish metrics between the main island groups of Tonga.

| **Richness**  *Raw data* |  |  |  |  |
| --- | --- | --- | --- | --- |
| Variable | Estimate | std. error | t value | p value |
| Intercept | 33.91 | 0.77 | 44.25 | <0.05 |
| Northern Ha'apai | 2.38 | 1.05 | 2.26 | <0.05 |
| Southern Ha'apai | 0.21 | 1.10 | 0.19 | 0.85 |
| Tongatapu | 0.21 | 1.02 | 0.21 | 0.83 |
| Vavau | -9.58 | 0.85 | -11.28 | <0.05 |
|  |  |  |  |  |
|  |  |  |  |  |
| **Density**  *Log(x)* |  |  |  |  |
| Variable | Estimate | std. error | t value | p value |
| Intercept | 7.55 | 0.06 | 128.63 | <0.05 |
| Northern Ha'apai | 0.08 | 0.08 | 0.97 | 0.33 |
| Southern Ha'apai | 0.01 | 0.08 | 0.10 | 0.92 |
| Tongatapu | -0.03 | 0.08 | -0.34 | 0.735 |
| Vavau | -0.44 | 0.07 | -6.73 | <0.05 |
|  |  |  |  |  |
|  |  |  |  |  |
| **Target biomass**  *Negative binomial* |  |  |  |  |
| Variable | Estimate | std. error | t value | p value |
| Intercept | 6.36 | 0.12 | 54.12 | <0.05 |
| Northern Ha'apai | 0.45 | 0.16 | 2.81 | <0.05 |
| Southern Ha'apai | 0.33 | 0.17 | 1.95 | 0.051 |
| Tongatapu | 0.09 | 0.16 | 0.58 | 0.56 |
| Vavau | -0.53 | 0.13 | -4.09 | <0.05 |

**
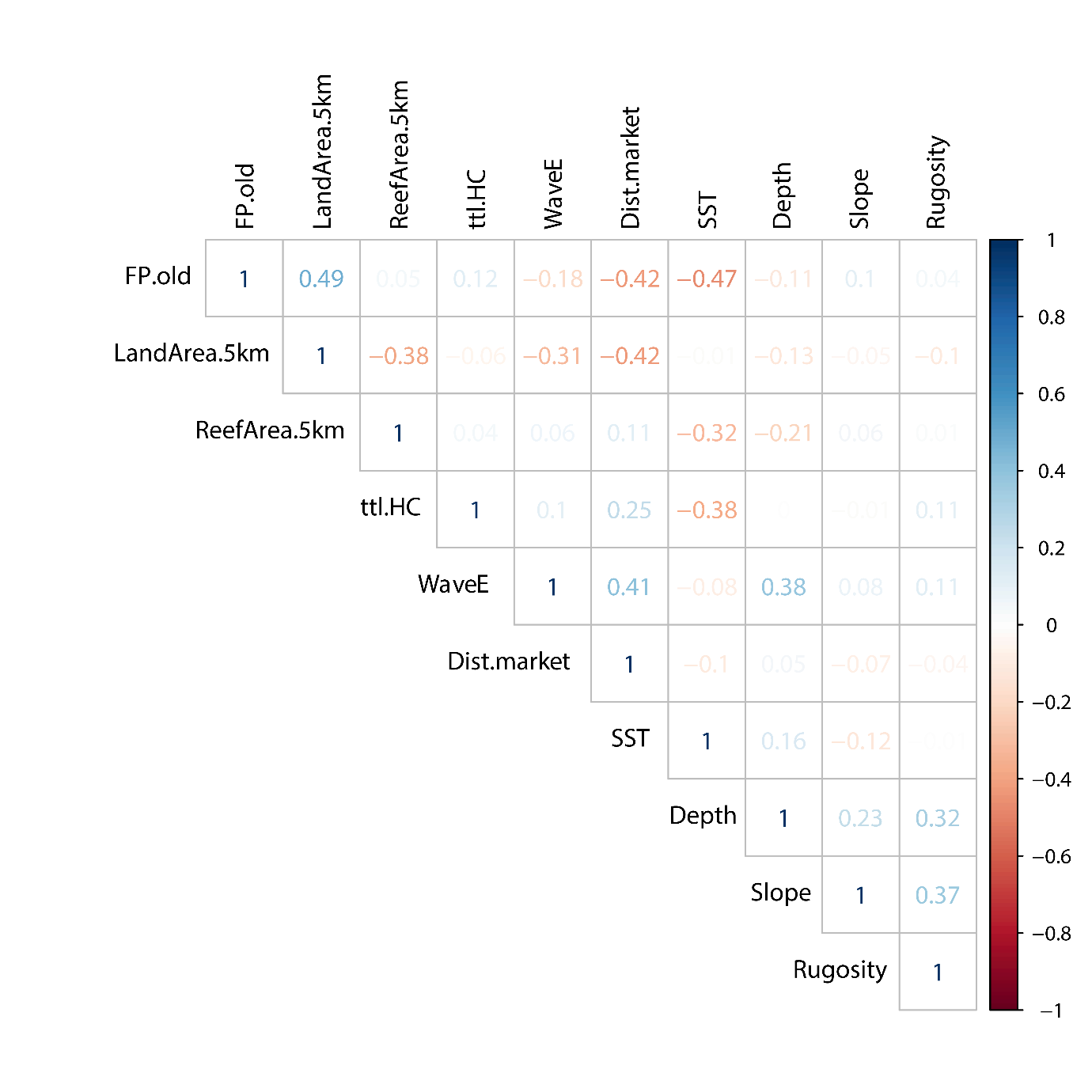
**

**Fig S12.** Correlation matrix of socio-environmental variables used to examine patterns of reef condition across Tonga.

**Table S4.** Boosted regression tree (BRT) parameters used to determine optimal tree complexity, bag fraction and learning rate for each of the four benthic response variables. The model with the greatest explained deviance while containing a minimum of 1000 trees was selected for the subsequent analysis (bold).

| **Variable** | **Tree complexity** | **Learning rate** | **Bag fraction** | **CV % Explained deviance** | **# Trees** |
| --- | --- | --- | --- | --- | --- |
| **Coral cover (poisson distribution)** | 3 | 0.01 | 0.25 | 36.7 | 2050 |
|  | **5** | **0.01** | **0.25** | **39.46** | **1150** |
|  | 7 | 0.01 | 0.25 | 36.45 | 1200 |
|  | 3 | 0.01 | 0.5 | 34.57 | 1100 |
|  | 5 | 0.01 | 0.5 | 36.71 | 600 |
|  | 7 | 0.01 | 0.5 | 36.46 | 650 |
|  | 3 | 0.01 | 0.75 | 34.18 | 1100 |
|  | 5 | 0.01 | 0.75 | 37.35 | 1000 |
|  | 7 | 0.01 | 0.75 | 40.15 | 550 |
|  | 3 | 0.001 | 0.25 | 36.6 | 9350 |
|  | 5 | 0.001 | 0.25 | 34.8 | 9250 |
|  | 7 | 0.001 | 0.25 | 35.76 | 8200 |
|  | 3 | 0.001 | 0.5 | 35.48 | 7750 |
|  | 5 | 0.001 | 0.5 | 39.27 | 6900 |
|  | 7 | 0.001 | 0.5 | 36.64 | 4650 |
|  | 3 | 0.001 | 0.75 | 37.37 | 9450 |
|  | 5 | 0.001 | 0.75 | 38.28 | 7000 |
|  | 7 | 0.001 | 0.75 | 37.09 | 6050 |
| **CCA (poisson distribution)** | 3 | 0.01 | 0.25 | 46.67 | 1850 |
|  | 5 | 0.01 | 0.25 | 49.09 | 1450 |
|  | 7 | 0.01 | 0.25 | 48.92 | 1700 |
|  | 3 | 0.01 | 0.5 | 48.89 | 1900 |
|  | 5 | 0.01 | 0.5 | 51.79 | 1100 |
|  | 7 | 0.01 | 0.5 | 50.02 | 700 |
|  | 3 | 0.01 | 0.75 | 50.68 | 2050 |
|  | **5** | **0.01** | **0.75** | **52.84** | **1250** |
|  | 7 | 0.01 | 0.75 | 48.83 | 700 |
|  | 3 | 0.001 | 0.25 | 48.27 | 9650 |
|  | 5 | 0.001 | 0.25 | 48.28 | 9600 |
|  | 7 | 0.001 | 0.25 | 48.46 | 8750 |
|  | 3 | 0.001 | 0.5 | 46.11 | 8650 |
|  | 5 | 0.001 | 0.5 | 51.06 | 8350 |
|  | 7 | 0.001 | 0.5 | 50.17 | 6450 |
|  | 3 | 0.001 | 0.75 | 48.63 | 9450 |
|  | 5 | 0.001 | 0.75 | 48.47 | 6950 |
|  | 7 | 0.001 | 0.75 | 50.82 | 7650 |
| **Soft coral (poisson distribution)** | 3 | 0.01 | 0.25 | 56.69 | 1350 |
|  | 5 | 0.01 | 0.25 | 56.62 | 600 |
|  | 7 | 0.01 | 0.25 | 53.92 | 550 |
|  | 3 | 0.01 | 0.5 | 57.05 | 1750 |
|  | 5 | 0.01 | 0.5 | 59.1 | 950 |
|  | 7 | 0.01 | 0.5 | 58.32 | 750 |
|  | 3 | 0.01 | 0.75 | 55.04 | 1150 |
|  | 5 | 0.01 | 0.75 | 56.68 | 650 |
|  | 7 | 0.01 | 0.75 | 57.38 | 700 |
|  | 3 | 0.001 | 0.25 | 55.99 | 9450 |
|  | 5 | 0.001 | 0.25 | 55.47 | 6250 |
|  | 7 | 0.001 | 0.25 | 54.07 | 4650 |
|  | 3 | 0.001 | 0.5 | 56.47 | 8600 |
|  | **5** | **0.001** | **0.5** | **58.22** | **7400** |
|  | 7 | 0.001 | 0.5 | 56.91 | 5900 |
|  | 3 | 0.001 | 0.75 | 54.75 | 8450 |
|  | 5 | 0.001 | 0.75 | 56.74 | 6050 |
|  | 7 | 0.001 | 0.75 | 55.55 | 6150 |
| **Turf algae (gaussian distribution)** | 3 | 0.01 | 0.25 | 51.74 | 550 |
|  | 5 | 0.01 | 0.25 | 50.94 | 550 |
|  | 7 | 0.01 | 0.25 | 51.13 | 400 |
|  | 3 | 0.01 | 0.5 | 52.73 | 550 |
|  | 5 | 0.01 | 0.5 | 51.51 | 400 |
|  | 7 | 0.01 | 0.5 | 51.83 | 350 |
|  | **3** | **0.01** | **0.75** | **53.84** | **1000** |
|  | 5 | 0.01 | 0.75 | 51.99 | 600 |
|  | 7 | 0.01 | 0.75 | 50.09 | 350 |
|  | 3 | 0.001 | 0.25 | 51.93 | 5450 |
|  | 5 | 0.001 | 0.25 | 51.85 | 5000 |
|  | 7 | 0.001 | 0.25 | 52.42 | 4650 |
|  | 3 | 0.001 | 0.5 | 50.55 | 4650 |
|  | 5 | 0.001 | 0.5 | 52.53 | 4300 |
|  | 7 | 0.001 | 0.5 | 51.79 | 3600 |
|  | 3 | 0.001 | 0.75 | 51.19 | 4750 |
|  | 5 | 0.001 | 0.75 | 51.73 | 4900 |
|  | 7 | 0.001 | 0.75 | 49.6 | 3350 |

**Table S5.** Boosted regression tree (BRT) parameters used to determine optimal tree complexity, bag fraction and learning rate for each of the three fish response variables. The model with the greatest explained deviance while containing a minimum of 1000 trees was selected for the subsequent analysis (bold).

| **Variable** | **Tree complexity** | **Learning rate** | **Bag fraction** | **CV % Explained deviance** | **# Trees** |
| --- | --- | --- | --- | --- | --- |
| **Reef fish density (poisson distribution)** | 3 | 0.01 | 0.25 | 27.41 | 900 |
|  | 5 | 0.01 | 0.25 | 24.42 | 500 |
|  | 7 | 0.01 | 0.25 | 25.59 | 700 |
|  | 3 | 0.01 | 0.5 | 27.47 | 650 |
|  | 5 | 0.01 | 0.5 | 29.86 | 450 |
|  | 7 | 0.01 | 0.5 | 27.92 | 350 |
|  | 3 | 0.01 | 0.75 | 27.93 | 1859 |
|  | 5 | 0.01 | 0.75 | 29.17 | 600 |
|  | 7 | 0.01 | 0.75 | 29.57 | 450 |
|  | 3 | 0.001 | 0.25 | 25.43 | 5450 |
|  | 5 | 0.001 | 0.25 | 26.29 | 4100 |
|  | 7 | 0.001 | 0.25 | 23.73 | 3800 |
|  | 3 | 0.001 | 0.5 | 25.19 | 5550 |
|  | 5 | 0.001 | 0.5 | 29.15 | 4150 |
|  | 7 | 0.001 | 0.5 | 29.22 | 3450 |
|  | 3 | 0.001 | 0.75 | 28.22 | 5800 |
|  | 5 | 0.001 | 0.75 | 27.17 | 3300 |
|  | **7** | **0.001** | **0.75** | **29.55** | **3650** |
| **Reef fish species richness (gaussian distribution)** | 3 | 0.01 | 0.25 | 66.98 | 1500 |
|  | 5 | 0.01 | 0.25 | 66.86 | 1900 |
|  | 7 | 0.01 | 0.25 | 67.82 | 1600 |
|  | 3 | 0.01 | 0.5 | 68.17 | 2300 |
|  | 5 | 0.01 | 0.5 | 69.2 | 1350 |
|  | **7** | **0.01** | **0.5** | **69.46** | **1100** |
|  | 3 | 0.01 | 0.75 | 67.13 | 1750 |
|  | 5 | 0.01 | 0.75 | 68.44 | 1250 |
|  | 7 | 0.01 | 0.75 | 68.88 | 900 |
|  | 3 | 0.001 | 0.25 | Did not converge | 10000+ |
|  | 5 | 0.001 | 0.25 | 66.9 | 7700 |
|  | 7 | 0.001 | 0.25 | 66.84 | 7350 |
|  | 3 | 0.001 | 0.5 | 66.25 | 9150 |
|  | 5 | 0.001 | 0.5 | 67.41 | 7300 |
|  | 7 | 0.001 | 0.5 | 68.97 | 6500 |
|  | 3 | 0.001 | 0.75 | Did not converge | 10000+ |
|  | 5 | 0.001 | 0.75 | 68.39 | 7850 |
|  | 7 | 0.001 | 0.75 | 67.36 | 6050 |
| **Target biomass (poisson distribution)** | 3 | 0.01 | 0.25 | 43.1 | 450 |
|  | 5 | 0.01 | 0.25 | 45.13 | 700 |
|  | 7 | 0.01 | 0.25 | 47.35 | 650 |
|  | 3 | 0.01 | 0.5 | 44.13 | 1750 |
|  | 5 | 0.01 | 0.5 | 44.23 | 400 |
|  | 7 | 0.01 | 0.5 | 45.83 | 500 |
|  | 3 | 0.01 | 0.75 | 41.04 | 1150 |
|  | 5 | 0.01 | 0.75 | 41.12 | 350 |
|  | 7 | 0.01 | 0.75 | 42.53 | 300 |
|  | 3 | 0.001 | 0.25 | 43.73 | 4400 |
|  | **5** | **0.001** | **0.25** | **46.27** | **4400** |
|  | 7 | 0.001 | 0.25 | 45.17 | 3950 |
|  | 3 | 0.001 | 0.5 | 44.58 | 7000 |
|  | 5 | 0.001 | 0.5 | 44.26 | 4050 |
|  | 7 | 0.001 | 0.5 | 44.71 | 3250 |
|  | 3 | 0.001 | 0.75 | 40.41 | 4150 |
|  | 5 | 0.001 | 0.75 | 43.35 | 4650 |
|  | 7 | 0.001 | 0.75 | 42.44 | 3600 |

**Table S6**. Summary of BRT model performance and spatial autocorrelation. As in Jouffray et al. (2019), model performances assessed on training data (used for model fitting) were higher than when assessed on the left out data (cross-validated). The cross-validated performance indicated how good the model is at predicting new data (Buston and Elith 2011). Moran’s I measures spatial autocorrelation and ranges from -1 to 1, with values close to 0 indicating no spatial autocorrelation.

|  | **Coral** | **CCA** | **Soft** | **Turf** | **Dens** | **Rich** | **BM** |
| --- | --- | --- | --- | --- | --- | --- | --- |
| Total.Deviance | 11.31 | 16.73 | 9.25 | 674.28 | 766.88 | 102.91 | 651.64 |
| Residual.Deviance | 2.89 | 3.03 | 1.89 | 206.10 | 281.71 | 7.31 | 231.01 |
| Correlation | 0.87 | 0.94 | 0.93 | 0.84 | 0.77 | 0.97 | 0.71 |
| Per.Expl | 74.47 | 81.92 | 79.55 | 69.43 | 63.27 | 92.90 | 64.55 |
| cvDeviance | 7.12 | 8.19 | 3.90 | 332.07 | 540.29 | 32.57 | 353.61 |
| cvCorrelation | 0.58 | 0.69 | 0.78 | 0.71 | 0.56 | 0.84 | 0.63 |
| cvPer.Expl | 37.04 | 51.06 | 57.83 | 53.84 | 29.55 | 68.35 | 45.73 |
| Morans I | 0.01 | -0.01 | 0.07 | -0.01 | 0.01 | -0.04 | 0.01 |

**Table S7.** Reef fish species list for Tonga from the 375 sites surveyed between 2016 and 2019. Species in bold were included as target species in the analysis based on Parks et al. (2017)

| Abudefduf sexfasciatus | Apogon hyallosoma | **Cephalopholis argus** | **Chlorurus sordidus** |
| --- | --- | --- | --- |
| Abudefduf sordidus | Apogon leptacanthus | **Cephalopholis leopardus** | Choerodon jordani |
| Abudefduf vaigiensis | Apogon luteus | **Cephalopholis miniata** | Chromis acares |
| **Acanthurus achilles** | Apogon nigrofasciatus | **Cephalopholis sonnerati** | Chromis agilis |
| **Acanthurus albipectoralis** | **Aprion virescens** | **Cephalopholis urodeta** | Chromis alpha |
| **Acanthurus blochii** | Arothron caeruleopunctatus | **Cetoscarus bicolor** | Chromis amboinensis |
| **Acanthurus grammoptilus** | Arothron hispidus | **Cetoscarus ocellatus** | Chromis analis |
| **Acanthurus guttatus** | Arothron manilensis | Chaetodon auriga | Chromis atripectoralis |
| **Acanthurus lineatus** | Arothron meleagris | Chaetodon baronessa | Chromis atripes |
| **Acanthurus maculiceps** | Arothron nigropunctatus | Chaetodon bennetti | Chromis bami |
| **Acanthurus nigricans** | Arothron stellatus | Chaetodon citrinellus | Chromis chrysura |
| **Acanthurus nigricauda** | Aspidontus taeniatus | Chaetodon ephippium | Chromis flavapicis |
| **Acanthurus nigrofuscus** | Asterropteryx semipunctata | Chaetodon flavirostris | Chromis flavomaculata |
| **Acanthurus nigroris** | Aulostomus chinensis | Chaetodon kleinii | Chromis iomelas |
| **Acanthurus olivaceus** | **Balistapus undulatus** | Chaetodon lineolatus | Chromis lepidolepis |
| **Acanthurus pyroferus** | **Balistoides conspiculum** | Chaetodon lunula | Chromis margaritifer |
| **Acanthurus thompsoni** | **Balistoides viridescens** | Chaetodon lunulatus | Chromis opercularis |
| **Acanthurus triostegus** | Blenniella chrysospilos | Chaetodon melanotus | Chromis retrofasciata |
| **Acanthurus xanthopterus** | Bodianus axillaris | Chaetodon mertensii | Chromis ternatensis |
| **Acanthurus dussumieri** | Bodianus dictynna | Chaetodon meyeri | Chromis tricincta |
| Aetobatus ocellatus | Bodianus loxozonus | Chaetodon ornatissimus | Chromis vanderbilti |
| Aluterus scriptus | Bodianus mesothorax | Chaetodon pelewensis | Chromis viridis |
| Amanses scopas | **Bothus mancus** | Chaetodon plebeius | Chromis weberi |
| Amblyeleotris fasciata | Bryaninops yongei | Chaetodon quadrimaculatus | Chromis xanthura |
| Amblyeleotris guttata | **Caesio caerulaurea** | Chaetodon rafflesi | Chrysiptera biocellata |
| Amblyeleotris periophthalma | **Caesio lunaris** | Chaetodon reticulatus | Chrysiptera brownriggii |
| Amblyeleotris steinitzi | **Caesio teres** | Chaetodon semeion | Chrysiptera rollandi |
| Amblyglyphidodon aureus | **Calostomus coralinus** | Chaetodon trifascialis | Chrysiptera starcki |
| Amblyglyphidodon melanopterus | Cantherhines dumerilii | Chaetodon ulietensis | Chrysiptera talboti |
| Amblygobius nocturnus | Cantherhines fronticinctus | Chaetodon unimaculatus | Chrysiptera taupou |
| Amblygobius phalaena | Cantherhines pardalis | Chaetodon vagabundus | Chrysiptera unimaculata |
| Amblygobius rainfordi | Canthigaster ambionensis | **Cheilinus chlorourus** | Cirrhilabrus punctatus |
| Amphiprion chrysopterus | Canthigaster axiologa | **Cheilinus fasciatus** | Cirrhitichthys falco |
| Amphiprion clarkii | Canthigaster bennetti | **Cheilinus oxycephalus** | Cirripectes chelomatus |
| Amphiprion melanopus | Canthigaster solandri | **Cheilinus trilobatus** | Cirripectes fuscoguttatus |
| Amphiprion pacificus | Canthigaster valentini | **Cheilinus undulatus** | Cirripectes polyzona |
| Amphiprion perideraion | Caracanthus maculatus | Cheilio inermis | Cirripectes stigmaticus |
| Anampses caeruleopunctatus | **Carangoides ferdau** | Cheiliodipterus artus | Coris aygula |
| Anampses geographicus | **Caranx ignobilis** | Cheilodipterus artus | Coris batuensis |
| Anampses melanurus | **Caranx melampygus** | Cheilodipterus isostigmus | Coris dorsomacula |
| Anampses meleagrides | **Caranx sexfasciatus** | Cheilodipterus macrodon | Coris gaimard |
| Anampses neoguinaicus | **Carcharhinus amblyrhynchos** | Cheilodipterus quinquelineatus | Corythoichthys intestinalis |
| Anampses twistii | Centropyge bicolor | **Chlorurus bleekeri** | **Ctenochaetus binotatus** |
| **Anyperodon leucogrammicus** | Centropyge bispinosus | **Chlorurus frontalis** | **Ctenochaetus cyanocheilus** |
| **Aphareus furca** | Centropyge flavissima | **Chlorurus japanensis** | **Ctenochaetus flavicauda** |
| Apogon cyanosoma | Centropyge heraldi | **Chlorurus microrhinus** | **Ctenochaetus hawaiiensis** |

| **Ctenochaetus striatus** | Halichoeres chrysus | **Lutjanus monostigma** | Ostorhinchus aureus |
| --- | --- | --- | --- |
| Ctenogobiops aurocingulus | Halichoeres hortulanus | **Lutjanus quinquelineatus** | Ostorhinchus cookii |
| Dascyllus aruanus | Halichoeres margaritaceus | **Macolor macularis** | Ostorhinchus cyanosoma |
| Dascyllus reticulatus | Halichoeres marginatus | **Macolor niger** | Ostorhinchus aureus |
| Dascyllus trimaculatus | Halichoeres melanochir | Macropharyngodon meleagris | Ostracion cubicus |
| **Dasyatis kuhlii** | Halichoeres melanurus | Macropharyngodon negrosensis | Ostracion meleagris |
| Diodon holocanthus | Halichoeres nebulosus | Malacanthus brevirostris | Oxycheilinus arenatus |
| Diodon hystrix | Halichoeres ornatissimus | Malacanthus latovittatus | Oxycheilinus celebicus |
| Echidna nebulosa | Halichoeres prosopeion | Meiacanthus atrodorsalis | **Oxycheilinus digramma** |
| Ecsenius bicolor | Halichoeres trimaculatus | Meiacanthus bundoon | Oxycheilinus nigromarginatus |
| Ecsenius flavus | **Hemigymnus fasciatus** | Meiacanthus ditrema | Oxycheilinus orientalis |
| Ecsenius midas | **Hemigymnus melapterus** | Meiacanthus procne | Oxycheilinus rhodochrous |
| **Elagatis bipinnulata** | Hemitaurichthys polylepis | Meiacanthus tongaensis | Oxycheilinus unifasciatus |
| Epibulus insidiator | Heniochus acuminatus | **Melichthys niger** | Oxymonacanthus longirostris |
| **Epinephelus fasciatus** | Heniochus chrysostomus | **Melichthys vidua** | **Paracanthurus hepatus** |
| **Epinephelus hexagonatus** | Heniochus monoceros | **Monotaxis grandoculis** | Paracirrhites arcatus |
| **Epinephelus howlandi** | Heniochus singularis | **Monotaxis heterodon** | Paracirrhites forsteri |
| **Epinephelus macrospilos** | Heniochus varius | **Mulloidichthys flavolineatus** | Paracirrhites hemistictus |
| **Epinephelus maculatus** | **Hipposcarus longiceps** | **Mulloidichthys pflugeri** | Paraluteres prionurus |
| **Epinephelus malabaricus** | Hologymnosus annulatus | **Mulloidichthys vanicolensis** | Parapercis australis |
| **Epinephelus merra** | Hologymnosus doliatus | **Myripristis adusta** | Parapercis clathrata |
| **Epinephelus ongus** | Istigobius goldmanni | **Myripristis berndti** | Parapercis cylindrica |
| **Epinephelus pasciatus** | Istigobius rigilius | **Myripristis kuntee** | Parapercis hexophthalma |
| **Epinephelus polyphekadion** | Koumansetta rainfordi | **Myripristis murdjan** | Parapercis millepunctata |
| **Epinephelus spilotoceps** | **Kyphosus cinerascens** | **Myripristis violacea** | **Parupeneus barberinoides** |
| Exallias brevis | **Kyphosus vaigiensis** | **Myripristis vittata** | **Parupeneus barberinus** |
| Exyrias bellisimus | Labrichthys unilineatus | **Naso brachycentron** | **Parupeneus bifasciatus** |
| Fistularia commersonii | Labroides bicolor | **Naso brevirostris** | **Parupeneus ciliatus** |
| Forcipiger flavissimus | Labroides dimidiatus | **Naso caesius** | **Parupeneus crassilabris** |
| Forcipiger longirostris | Labroides pectoralis | **Naso hexacanthus** | **Parupeneus cyclostomus** |
| Fusigobius signipinnis | Labroides rubrolabiatus | **Naso lituratus** | **Parupeneus indicus** |
| **Gnathanodon speciosus** | Labropsis australis | **Naso lopezi** | **Parupeneus multifasciatus** |
| **Gnathodentex aureolineatus** | **Leptoscarus vaigiensis** | **Naso tonganus** | **Parupeneus pleurostigma** |
| Gobiodon citrinus | **Lethrinus harak** | **Naso unicornis** | **Parupeneus spilurus** |
| Gomphosus varius | **Lethrinus nebulosus** | Nectamia fusca | Pempheris oualensis |
| **Grammistes sexlineatus** | **Lethrinus obsoletus** | Nemateleotris magnifica | Pervagor alternans |
| **Gymnocranius euanus** | **Lethrinus olivaceus** | Neocirrhitus armatus | Pervagor aspricaudus |
| **Gymnocranius microdon** | **Lutjanus biguttatus** | Neoglyphidodon carlsoni | Pervagor janthinosoma |
| **Gymnosarda unicolor** | **Lutjanus bohar** | **Neoniphon argenteus** | Pervagor melanocephalus |
| Gymnothorax buroensis | **Lutjanus carponotatus** | **Neoniphon opercularis** | Petroscirtes mitratus |
| Gymnothorax flavimarginatus | **Lutjanus ehrenbergii** | **Neoniphon sammara** | Plagiotremus flavus |
| Gymnothorax javanicus | **Lutjanus fulviflamma** | Neopomacentrus azyron | Plagiotremus laudandus |
| Gymnothorax meleagris | **Lutjanus fulvus** | Neopomacentrus metalicus | Plagiotremus rhinorhynchos |
| Gymnothorax nudivomer | **Lutjanus gibbus** | Novaculichthys taeniourus | Plagiotremus tapeinosoma |
| Halichoeres argus | **Lutjanus kasmira** | Novaculoides macrolepidotus | **Platax boersii** |
| Halichoeres biocellatus | **Lutjanus malabaricus** | Odonus niger | **Platax teira** |

| **Plectorhinchus chaetodonoides** | Pteragogus cryptus | **Scomberoides lysan** |
| --- | --- | --- |
| **Plectorhinchus lineatus** | Ptereleotris evides | Scorpaenopsis macrochir |
| **Plectorhinchus picus** | Ptereleotris hanae | Sebastapistes cyanostigma |
| Plectroglyphidodon dickii | Ptereleotris heteroptera | Siderea thyrsoidea |
| Plectroglyphidodon imparipennis | Ptereleotris microlepis | **Siganus argenteus** |
| Plectroglyphidodon johnstonianus | Ptereleotris monoptera | **Siganus doliatus** |
| Plectroglyphidodon lacrymatus | **Pterocaesio digramma** | **Siganus niger** |
| Plectroglyphidodon leucozonus | **Pterocaesio marri** | **Siganus punctatus** |
| Plectropomus laevis | **Pterocaesio tile** | **Siganus spinus** |
| Plectropomus leopardus | **Pterocaesio trilineata** | **Siganus stellatus** |
| Plotosus lineatus | Pterois radiata | **Siganus vulpinus** |
| **Pomacanthus imperator** | Pterois volitans | Siphamia jebbi |
| Pomacentrus adelus | **Pygoplites diacanthus** | **Sphyraena barracuda** |
| Pomacentrus amboinensis | **Rastrelliger kanagurta** | **Sphyraena helleri** |
| Pomacentrus brachialis | **Rhinecanthus aculeatus** | Stegastes albifasciatus |
| Pomacentrus callainus | **Rhinecanthus rectangulus** | Stegastes fasciolatus |
| Pomacentrus chrysurus | Salarias fasciatus | Stegastes lividus |
| Pomacentrus coelestis | Salarias nigrocinctus | Stegastes nigricans |
| Pomacentrus imitator | **Sargocentron caudimaculatum** | Stegastes punctatus |
| Pomacentrus maafu | **Sargocentron diadema** | Stethojulis bandanensis |
| Pomacentrus margaritifer | **Sargocentron ittodai** | Stethojulis notialis |
| Pomacentrus microspilus | **Sargocentron melanospilos** | Stethojulis strigiventer |
| Pomacentrus moluccensis | **Sargocentron spiniferum** | Sufflamen bursa |
| Pomacentrus pavo | **Sargocentron tiere** | Sufflamen chrysopterum |
| Pomacentrus philippinus | **Sargocentron violaceum** | Synchiropus splendidus |
| Pomacentrus spilotoceps | Saurida gracillis | Synodus binotatus |
| Pomacentrus vaiuli | **Scarus altipinnis** | Synodus dermatogenys |
| Pomacentrus wardi | **Scarus chamaeleon** | Synodus variegatus |
| Pomachromis richardsoni | **Scarus dimidiatus** | Taeniamia fucata |
| **Priacanthus arenatus** | **Scarus flavipectoralis** | Thalassoma amblycephalum |
| **Priacanthus blochii** | **Scarus forsteni** | Thalassoma hardwicke |
| **Priacanthus hamrur** | **Scarus frenatus** | Thalassoma jansenii |
| Pristiapogon exostigma | **Scarus ghobban** | Thalassoma lunare |
| Pristiapogon fraenatus | **Scarus globiceps** | Thalassoma lutescens |
| Pristiapogon kallopterus | **Scarus longipinnis** | Thalassoma nigrofasciatum |
| Pseudanthias dispar | **Scarus niger** | Thalassoma quinquevittatum |
| Pseudanthias pleurotaenia | **Scarus oviceps** | Thalossoma purpureum |
| Pseudanthias squamipinnis | **Scarus psittacus** | **Triaenodon obesus** |
| **Pseudobalistes flavimarginatus** | **Scarus rivulatus** | Valenciennea immaculata |
| Pseudobalistes fuscus | **Scarus rubrovialaceous** | Valenciennea parva |
| Pseudocheilinus evanidus | **Scarus schlegeli** | Valenciennea puellaris |
| Pseudocheilinus hexataenia | **Scarus spinus** | Valenciennea sexguttata |
| Pseudocheilinus octotaenia | **Scarus tricolor** | Valenciennea strigata |
| Pseudocoris heteroptera | Scolopsis bilineatus | **Variola louti** |
| Pseudocoris yamashiroi | Scolopsis lineatus | Zanclus cornutus |
| Pseudojuloides cerasinus | Scolopsis trilineata | **Zebrasoma scopas** |
|  |  | **Zebrasoma veliferum** |
|  |  | Zoramia fragilis |
|  |  | Zoramia leptacantha |
|  |  | Zoramia viridiventer |

**References:**

Andrefouet, S., Muller-Karger, F.E., Robinson, J.A., Kranenburg, C.J., Torres-Pulliza, D., Spraggins, S.A. and Murch, B., 2006. Global assessment of modern coral reef extent and diversity for regional science and management applications: a view from space. In *Proceedings of the 10th International Coral Reef Symposium* (Vol. 2, pp. 1732-1745). Japanese Coral Reef Society Okinawa, Japan.

Bode, M., Leis, J.M., Mason, L.B., Williamson, D.H., Harrison, H.B., Choukroun, S. and Jones, G.P., 2019. Successful validation of a larval dispersal model using genetic parentage data. *PLoS biology*, *17*(7), p.e3000380.

Brett, J. R. 1971. Energetic responses of salmon to temperature. A study of some thermal relations in physiology and freshwater ecology of sockeye salmon (Oncorhynchus nerka). *American Zoologist* 11:99-118.

Brewer, T. D., Cinner, J. E., Fisher, R., Green, A., & Wilson, S. K., 2012. Market access, population density, and socioeconomic development explain diversity and functional group biomass of coral reef fish assemblages. *Global Environmental Change*, *22*(2), 399-406.

Buston, P.M. and Elith, J., 2011. Determinants of reproductive success in dominant pairs of clownfish: a boosted regression tree analysis. Journal of Animal Ecology, 80(3), pp.528-538.

Cinner, J., & McClanahan, T. R., 2006. Socioeconomic factors that lead to overfishing in small-scale coral reef fisheries of Papua New Guinea. *Environmental Conservation*, *33*(1), 73-80.

Cinner, J. E., Graham, N. A., Huchery, C., & MacNeil, M. A., 2013. Global effects of local human population density and distance to markets on the condition of coral reef fisheries. *Conservation Biology*, *27*(3), 453-458.

Cinner, J. E., Maire, E., Huchery, C., MacNeil, M. A., Graham, N. A., Mora, C., ... & D’agata, S., 2018. Gravity of human impacts mediates coral reef conservation gains. *Proceedings of the National Academy of Sciences*, *115*(27), E6116-E6125.

De’ath, G., Fabricius, K. E., Sweatman, H., & Puotinen, M. (2012). The 27–year decline of coral cover on the Great Barrier Reef and its causes. *Proceedings of the National Academy of Sciences*, *109*(44), 17995-17999.

Devlin, M. J. and Brodie, J. 2005. Terrestrial discharge into the Great Barrier Reef Lagoon: nutrient behavior in coastal waters. *Marine Pollution Bulletin* **51**: 9-22.

Done, T. J. (1992). Effects of tropical cyclone waves on ecological and geomorphological structures on the Great Barrier Reef. *Continental Shelf Research*, *12*(7-8), 859-872.

Ekebom, J., P. Laihonen, and T. Suominen. 2003. A GIS-based step-wise procedure for assessing physical exposure in fragmented archipelagos. *Estuarine Coastal and Shelf Science* 57:887-898

Fabricius, K. E. 2005. Effects of terrestrial runoff on the ecology of corals and coral reefs: review and synthesis. *Marine pollution bulletin* **50**:125-146.

Fabricius, K. E., De'Ath, G., Puotinen, M. L., Done, T., Cooper, T. F., & Burgess, S. C. (2008). Disturbance gradients on inshore and offshore coral reefs caused by a severe tropical cyclone. *Limnology and Oceanography*, *53*(2), 690-704.

Fulton, C. J., Bellwood, D.R. and Wainwright, P.C.. 2005. Wave energy and swimming performance shape coral reef fish assemblages*. Proceedings Of The Royal Society B-Biological Science*s 272:827-832.

Green, A. L., Maypa, A. P., Almany, G. R., Rhodes, K. L., Weeks, R., Abesamis, R. A., ... & White, A. T. (2015). Larval dispersal and movement patterns of coral reef fishes, and implications for marine reserve network design. *Biological Reviews*, *90*(4), 1215-1247.

Harborne, A. R., 2016. The Nature Conservancy’s mapping ocean wealth project and the current and potential standing stock of coral reef fishes in five juristictions of Micronesia. Final Technical Report

Hughes, T. P., Kerry, J. T., Álvarez-Noriega, M., Álvarez-Romero, J. G., Anderson, K. D., Baird, A. H., ... & Wilson, S. K., 2017. Global warming and recurrent mass bleaching of corals. *Nature*, *543*(7645), 373.

Jenness, J.S. and Houk, P.. 2014. UOGML Wave Energy ArcGIS Extension. University of Guam Marine Laboratory.

Jouffray, J. B., Wedding, L. M., Norström, A. V., Donovan, M. K., Williams, G. J., Crowder, L. B., ... & Kappel, C. V. (2019). Parsing human and biophysical drivers of coral reef regimes. *Proceedings of the Royal Society B*, *286*(1896), 20182544.

Kronen, Mecki, 2004. “Fishing for Fortunes? A Socio-Economic Assessment of Tonga’s Artisanal Fisheries.” *Fisheries Research* 70(1):121–34.

Lapointe, B. E. and Clark, M. W., 1992. Nutrient inputs from the watershed and coastal eutrophication in the Florida Keys. *Estuaries* **15**:465-476.

Ochiewo, J., 2004. Changing fisheries practices and their socioeconomic implications in South Coast Kenya. *Ocean & Coastal Management*, *47*(7-8), 389-408.

Parks, J., (2017). Baseline Socioeconomic Survey of the Vava‘u Special Management Areas (SMA) As a Component of the Tonga Climate Resilience Sector Project MAFFF/MEIDECC Kingdom of Tonga.

Puotinen, M. L. (2007). Modelling the risk of cyclone wave damage to coral reefs using GIS: a case study of the Great Barrier Reef, 1969–2003. *International Journal of Geographical Information Science*, *21*(1), 97-120.

Purkis SJ 2018. Remote sensing tropical coral reefs: The view from above. *Annual Review of Marine Science 10:149-168*

Sbrocco, Elizabeth J. and Paul H. Barber., 2013. “MARSPEC: Ocean Climate Layers for Marine Spatial Ecology.” *Ecology* 94(4):979–979.

Smallhorn‐West, P. F., Bridge, T. C., Malimali, S. A., Pressey, R. L., & Jones, G. P., 2019. Predicting impact to assess the efficacy of community‐based marine reserve design. *Conservation Letters*, *12*(1), e12602.

Statistics Department of Tonga, 2016. *Tonga national population and housing census*. Nuku'alofa, Tonga Tongan Bureau of Statistics.

Yeager, L.A., Marchand, P. Gill, D.A., Baum, J.K. and McPherson, J.M., 2017. “Marine Socio-Environmental Covariates: Queryable Global Layers of Environmental and Anthropogenic Variables for Marine Ecosystem Studies.” *Ecology* 98(7):1976.
